# Matrix coating assisted by an electric field (MCAEF) for enhanced tissue imaging by MALDI-MS†
†Electronic supplementary information (ESI) available: Additional experimental details and supporting data. See DOI: 10.1039/c4sc01850h



**DOI:** 10.1039/c4sc01850h

**Published:** 2014-09-16

**Authors:** Xiaodong Wang, Jun Han, Juncong Yang, Jingxi Pan, Christoph H. Borchers

**Affiliations:** a University of Victoria – Genome British Columbia Proteomics Centre , Vancouver Island Technology Park, #3101-4464 Markham St. , Victoria , BC V8Z 7X8 , Canada . Email: christoph@proteincentre.com ; Fax: +1-250-483-3238 ; Tel: +1-250-483-3221; b Department of Biochemistry and Microbiology , University of Victoria , Petch Building Room 207, 3800 Finnerty Rd. , Victoria , BC V8P 5C2 , Canada

## Abstract

A novel technique, termed matrix coating assisted by an electric field (MCAEF), for enhancing tissue imaging by matrix-assisted laser desorption/ionization mass spectrometry (MALDI-MS) was developed in this study. In this technique a static and uniform electric field is applied to sliced tissue sections during matrix spray-coating, resulting in the enrichment of positively or negatively chargeable analytes in the MALDI matrix layer. Experimental results show that MCAEF not only increased the sensitivity of lipid and protein detection across the board in the subsequent MALDI-MS analyses, but also resulted in successful imaging of a larger number of analytes. MALDI imaging enhancement with MCAEF was observed for various tissues (rat liver, rat brain, and porcine adrenal gland) and with different MALDI matrices (*e.g.*, quercetin, 2-mercaptobenzothiazole, dithranol, 9-aminoacridine, and sinapinic acid) and the sensitivity increases were independent of the solvent compositions and pH values of the matrix solutions. Taking rat brain as an example, MCAEF led to the on-tissue detection and imaging of 648 identified lipids by combining positive and negative ion detection by MALDI-Fourier transform ion cyclotron resonance MS and with quercetin as the matrix, as compared to only 344 lipids without MCAEF. For protein imaging, up to 232 protein signals were successfully detected in rat brain tissue sections by MALDI-time-of-flight MS within a mass range of 3500 to 37 000 Da, as compared to 119 without MCAEF. MCAEF also enabled the detection of higher molecular-weight proteins. These results demonstrate the advantages of MCAEF for overall performance improvements in MALDI imaging and we believe that this technique has the potential to become a standard practice for MALDI tissue imaging.

## Abstract 2

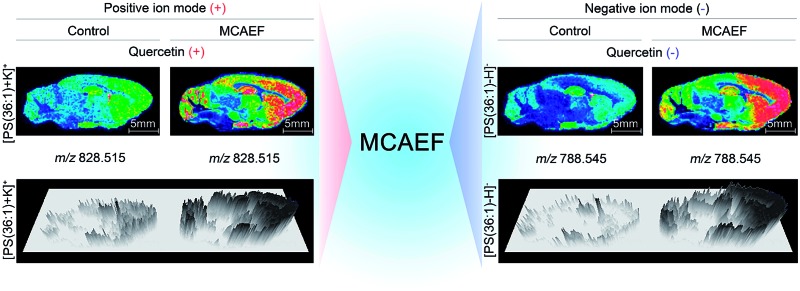
A novel technique, termed matrix coating assisted by an electric field (MCAEF) for enhancing tissue imaging by matrix-assisted laser desorption/ionization mass spectrometry (MALDI-MS) was developed in this study.

## Introduction

Tissue imaging by matrix-assisted laser desorption/ionization mass spectrometry (MALDI-MS) is a well-established technology to simultaneously explore and characterize the spatial distributions and relative abundances of endogenous compounds directly from the surface of a thinly-cut tissue slice.^1–7^ It capitalizes on the high sensitivity, high specificity, and high throughput of state-of-the-art mass spectrometers to produce visual images of various ionized species within tissue samples, including lipids and proteins. The locations and abundances of specific biomolecules reflect the pathophysiology of the imaged tissue specimens,^1,8,9^ so MALDI imaging has great potential for human disease biomarker discovery.^5,10^

There are several thousand lipids in a cell or a tissue,^11,12^ and MS/MS spectra of 119 200 lipid compounds have already been entered into the LipidBlast library.^13^ Using LC-MS/MS methods, 2800 proteins can be detected in human colon adenoma tissue.^14^ Despite the enormous progress has been made in MALDI tissue imaging, most imaging studies report relatively small numbers of analytes in their analyses, for example, 212 lipids were reportedly detected in an imaging study of rat brain,^15^ 544 lipids in porcine adrenal gland,^16^ 92 proteins in mouse lung, and 105 proteins in mouse kidney.^17,18^ From the comparison of these numbers, it is clear that the MALDI imaging is not reaching its full potential. Many MS imaging studies have focused on screening new MALDI matrices in order to enhance the detection of analytes on tissue sections.^1,2,19–21^ MALDI matrices that have been proven to be useful for imaging low molecular weight compounds include 1,5-diaminonapthalene (DAN),^22^ 2-mercapto-benzothiazole (2-MBT),^23^ 2,5-dihydroxybenzoic acid (DHB),^24^ 2,6-dihydroxyacetophenone (DHA),^25–28^ 4-*para*-nitroaniline (*p*NA),^29,30^ 9-aminoacridine (9-AA),^31^ dithranol,^32^ curcumin,^33^ and hydroxyflavones (*e.g.*, quercetin and morin).^15,16^ For proteomic imaging, sinapinic acid (SA),^34–36^ DHB,^34–36^ and α-cyano-4-hydroxycinnamic acid (CHCA)^34,35^ are the most commonly used MALDI matrices. Currently, SA is regarded as the best matrix for MALDI-MS of larger proteins while DHB and CHCA are preferred for the analysis of peptides and small proteins.^34^ In addition, some ionic matrices have also been proposed for tissue imaging by MALDI-MS in order to overcome problems associated with low stability under vacuum and/or crystal inhomogeneity. Some examples of ionic matrices are DHA/aniline,^37^ CHCA/aniline,^38^ DHB/aniline,^39^ DHB/3-acetylpyridine,^39^ DHB/pyridine,^39^ and DHB or CHCA/butylamine.^40^

In addition to the screening of new matrices, a few studies have turned their attention to improved sample preparation, which is another critical aspect for achieving high sensitivity, reproducibility, and improved visualization.^41^ Many methods have been developed for the optimization of sample preparation to improve endogenous compound imaging,^24,42–44^ such as matrix sublimation,^22,45–47^ matrix vapor deposition/recrystallization,^48^ matrix pre-coating,^49^ solvent-free matrix dry-coating,^50^ matrix microspotting,^51^ automated inkjet matrix printing,^52^ and tissue pre-washing before matrix coating.^34,35,53^ Most of these techniques have been shown to be suitable for the detection and imaging of low molecular-weight compounds (*e.g.*, lipids) or proteins in the positive ion mode. Even with these new techniques, spray coating is still the predominant approach for matrix deposition in the MALDI-MS imaging practice. A technique for overall improvement of the performance of all of these MALDI imaging studies for both small and large biomolecules is needed.

Our hypothesis was that placing a charged plate above the tissue-mounted MALDI target during the matrix spray coating would result in oppositely charged analytes being pulled into the matrix layer, which would result in improved MS detection and imaging. In this technique, which we call matrix coating assisted by an electric field (MCAEF), a static electric field is applied to tissue sections during the process of matrix deposition *via* spray coating. Our experimental results show that MCAEF significantly enhances MALDI-MS imaging of both lipids and proteins in tissue sections.

## Results and discussion

### Design and optimization of MCAEF

**Fig. 1:**
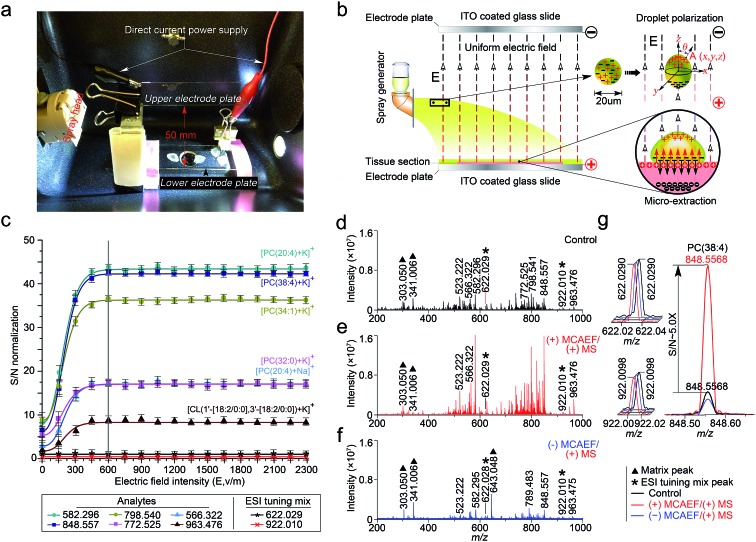
Design and optimization of MCAEF. (a) A photograph of the MCAEF set-up inside an electronic matrix sprayer. (b) Schematic diagram of MCAEF mechanism. (c) Effect of electric field intensity (*E*) on *S*/*N*s of six lipids detected by positive-ion (+) MALDI-FTICR MS. The error bars reflect nine mass spectra acquired from triplicate tissue slides that were mounted on three ITO-coated microscopic glass slides. (d) (+) MALDI-FTICR mass spectra of lipids detected on rat liver tissue sections without MCAEF. (e) (+) MALDI-FTICR mass spectra of lipids detected from rat liver tissue sections with (+) MCAEF. (f) (+) MALDI-FTICR mass spectra of lipids detected from rat liver tissue sections with (–) MCAEF. Two ESI generated ions from the Agilent “ESI tuning mix” solution (*i.e.*, *m*/*z* 622.029 and *m*/*z* 922.010), were used as the controls and are labelled with black asterisks. The *m*/*z* 922.010 ion was used for peak intensity normalization. The matrix-related ions are labeled “▲”, including *m*/*z* 303.050 [quercetin + H]^+^, *m*/*z* 341.006 [quercetin + K]^+^, and *m*/*z* 643.048 [2quercetin + K]^+^. (g) Overlay of mass spectra of the “ESI tuning mix” peaks of *m*/*z* 622.029 and *m*/*z* 922.010, and PC(38 : 4) of *m*/*z* 848.557 with and without MCAEF.

This study was designed to determine if matrix deposition assisted by an electric field would improve the performance of on-tissue detection and imaging by MALDI-MS. We used a Bruker ImagePrep electronic sprayer to coat MALDI matrices onto mammalian tissue sections (the use of the animal organs involved in this study was in accordance with current requirements of the Canadian Council on Animal Care and was approved by the Ethics Committee of the University of Victoria). During the entire matrix coating process using the electronic sprayer, a uniform electric field was applied onto the tissue sections that were mounted on the conductive side of ITO-coated microscopic glass slides. A photograph of the apparatus is shown in Fig. 1a. In this design, the tissue-mounted conductive glass slide acted as a positive or negative electrode plate, while a blank slide of the same type was placed in parallel to the tissue-mounted glass slide inside the sprayer chamber as an opposite-polarity electrode plate. The distance between the two slides was set at 50 mm. The conductive sides of the two slides were placed face-to-face. A direct current (DC) power supply was used to apply a static voltage to the two slides so as to form a uniform electric field between the two electrode plates. The polarity on each electrode plate was dependent on the subsequent MS detection mode. For positive-ion detection, a DC voltage was applied to the tissue mounted slide, as indicated in the diagram of Fig. 1b. For negative-ion detection mode, the electric field direction would be reversed.

Under an appropriate electric field intensity (*E*), the fine matrix droplets, *ca.* 20 μm in diameter according to the ImagePrep user's manual, that are generated by the electronic matrix sprayer would be polarized (Fig. 1b). According to the literature,^54^ the charge density (*ρ*_A_) at a point A (*x*, *y*, *z*) on the surface of a droplet (a perfectly conducting sphere) in a uniform electric field can be calculated as

1 *ρ*_A_ = 3*ε*_0_*ε*_r_*E* cos *θ*

where *ε*_0_ is the vacuum permittivity, *ε*_0_ = 8.8542 × 10^–12^ F m^–1^ *ε*_r_ is the relative permittivity (permittivity being defined as a measure of the response of a substance to an electric field). In this study, *ε*_r_ is the relative permittivity of nitrogen (N_2_) since the spray was performed in a nitrogen atmosphere, and *ε*_r_(N_2_) = 1.00058 (20 °C); *E* is the electric field intensity; and *θ* is the angle between *R*_A_ (A radius) and the electric field direction (Fig. 1b). Thus, the electric field force of point A (*F*_A_) can be calculated according to the following equation:

2 *F*_A_ = *ρ*_A_*E*Δ*S*_A_ = 3*ε*_0_*ε*_r_*E*^2^Δ*S*_A_ cos *θ*

where Δ*S*_A_ is the unit area occupied by point A. According to eqn (1) and (2), the different *F*_A_ values applied to different positions of a spherical droplet will result in inhomogeneous charge distribution on the droplet surface, which leads to droplet elliptical deformation. The maximum charge density appears at both ends of the polar axis (parallel to *E*) of a droplet (*i.e.*, *θ* = 0° and 180°), but with opposite net charges.

As illustrated in Fig. 1b, when the direction of the applied *E* is from the tissue-mounted glass slide toward the blank glass slide, the electric potential of the upper part of a matrix droplet is higher than that of the lower part of the droplet. The polarized matrix solution droplets on the surface of a thinly-cut tissue section cause positively chargeable analytes to enter the matrix layer, as opposed to negatively chargeable analytes. This process can be regarded as an electric field-driven micro-extraction that will ultimately enrich the matrix layer in positively chargeable analytes, in accordance with Coulomb's law which describes the electrostatic interaction between electrically charged particles. It was hypothesized that this matrix coating process, named MCAEF, would generate higher concentrations of the positively chargeable analytes per unit volume of matrix and would therefore enhance the detection of these analytes using positive ion MALDI. When the electric field direction is reversed during the matrix coating, it would result in enrichment of negatively chargeable analytes in the matrix layer coated on the surface of a tissue section, which would enhance MALDI-MS of these analytes in the negative ion mode.

To test this hypothesis, we used a series of 12 μm thick tissue sections prepared from a same rat liver and coated them with quercetin (a useful MALDI matrix for lipidomic MALDI imaging^15,16^). During the matrix coating, different DC voltages, ranging from 0 to +115 V (equivalent to *E* = 0 to 2300 V m^–1^), were applied to the tissue-mounted slides. According to previous studies,^55,56^ the relatively low DC voltages (<120 V) will not generate a voltage *E* strong enough to split a neutral matrix solution droplet into two smaller charged droplets.

The quercetin matrix solution was at its optimal concentration of 2.6 mg mL^–1^ prepared in 80 : 20 : 0.1 (v/v) methanol–water–NH_4_OH.^15^ After matrix coating using the same procedure as previously described,^15^ these tissue sections were subjected to positive-ion MALDI-FTICR MS using the same set of instrumental operation parameters. Six randomly selected lipids with different ion intensities, which were detected on all the tissue sections, including five phosphatidylcholines (PCs) and one cardiolipin (CL), *i.e.*, [PC(20 : 4) + Na]^+^ (*m*/*z* 566.322), [PC(20 : 4) + K]^+^ (*m*/*z* 582.296), [PC(32 : 0) + K]^+^ (*m*/*z* 772.525), [PC(34 : 1) + K]^+^ (*m*/*z* 798.541), [PC(38 : 4) + K]^+^ (*m*/*z* 848.557), and [CL(1′-[18 : 2/0 : 0],3′-[18 : 2/0 : 0]) + K]^+^ (*m*/*z* 963.476), were selected as the representatives for calculation of the signal-to-noise ratios (*S*/*N*s) in order to compare and optimize the applied *E*. Two ions (at *m*/*z* 622.029 and 922.010), generated by infusing the Agilent “ESI tuning mix” solution from the electrospray (ESI) side of the ion source during the MALDI acquisitions, were used as the MALDI-process independent internal standards, and the ion at *m*/*z* 922.010 was also used for peak intensity normalization. In order to evaluate the reproducibility of MCAEF, three replicates were carried out for each experiment.

Fig. 1c shows that the normalized *S*/*N*s of the 6 lipid ions were significantly increased when an electric field was applied, compared to the electric field-free (*i.e.*, *E* = 0) matrix coating. In addition, the observed *S*/*N*s were directly proportional to the applied DC voltages and reached a plateau when *E* was +600 and until +2300 V m^–1^. To determine the possible reason for the plateau when *E* was 600 V cm^–1^ and above, we coated two rat liver tissue sections with quercetin, with and without the use of MCAEF during matrix coating. After MALDI-FTMS analysis, the coated quercetin was removed from surfaces of the two tissue sections by manually spraying a minimum amount of methanol. The two tissue sections were then recoated with the same matrix solution again with ImagePrep under the optimal matrix coating conditions followed by MALDI-FTMS. As shown in Fig. S1,† on the tissue section for which MCAEF was applied during the first matrix coating, no analyte signals were observed except for several matrix-related signals. For the other tissue section, where no MCAEF was applied during the first matrix coating, many lipid signals were observed. A UPLC-MS experiment was also performed to evaluate if the plateau effect resulted from a higher extent of extraction of the MALDI-amenable analytes from the tissue sections. To do this, the tissues from two rat brain tissue sections where MCAEF was used during the matrix coating and from two other tissue sections where MCAEF was not used, were scraped off. The lipids were then extracted with methanol : chloroform : water (3 : 1 : 1, v : v : v), and were chromatographed by UPLC-(+)ESI-MS on a reversed-phase C4 column with 0.01% formic acid in water (solvent A) and 0.01% formic acid in acetonitrile (solvent B) as the mobile phase for gradient elution. As shown in the supplementary information (Fig. S2†), the MALDI-imaged lipids were detected by UPLC-(+)ESI-MS with much higher intensities for the tissue sections where MCAEF had not been used during matrix coating. In contrast, for the tissue sections for which MCAEF had been used during matrix coating, the same lipids were also detected, but at much lower intensities, as represented by the six randomly selected lipids shown in Fig. S2b.† These comparisons indicate that MCAEF induced fairly complete extraction of the MALDI-amenable analytes from the surface of the tissue section into the matrix layers, which might account for the plateau of *E* = 600 V cm^–1^ and above. No higher *E* was tested because the maximum of the allowable output voltage of the DC power supply was only 120 V. The mass spectra acquired in positive ion MALDI-FTICR MS from two rat liver sections at *E* = 0 (control) and 600 V m^–1^, respectively, are shown in Fig. 1d and e.

As described above, our hypothesis was that the electric field-driven enrichment of the chargeable analytes from the tissue surface into the thin matrix layer was the major reason for the improved MALDI detection. To confirm this, we reversed the direction of electric field, *i.e.* different negative DC voltages were applied to the tissue mounted glass slides. This was expected to induce migration of the negatively chargeable analytes from the tissue surface into the thin matrix layer which would lower the detectability of positively charged analytes by positive-ion MALDI-MS. As expected, poorer detection of the analytes (dominantly lipids) on these tissue sections was observed in the positive ion mode, as compared to that from the electric field-free tissue section. Fig. 1f shows the mass spectrum acquired from the tissue section with an applied electric field at *E* = –600 V m^–1^. The matrix-related signals dominate this mass spectrum and much weaker lipid signals are observed than those in the mass spectrum acquired with *E* = 0. At *E* = +600 V m^–1^, signals from the detected compounds showed an overall increase in ion intensity, as compared to the control mass spectrum. Taking the [PC(38 : 4) + K]^+^ (*m*/*z* 848.557) ion as an example, a *ca.* 5-fold *S*/*N* increase (Fig. 1g) was observed.

### Effect of electric field during different steps of the matrix spray coating

In this study, matrix coating was carried using the Bruker ImagePrep electronic sprayer with the optimal number of spray cycles to coat a thinly-cut tissue section with each matrix. Each spray cycle, as described in the experiment section in the supplementary information,† was composed of a 3 s spray step, a 60 s incubation step, and a 90 s drying step. As shown in Fig. S3,† 30 spray cycles was shown to be optimal for the matrix coating.

**Fig. 2:**
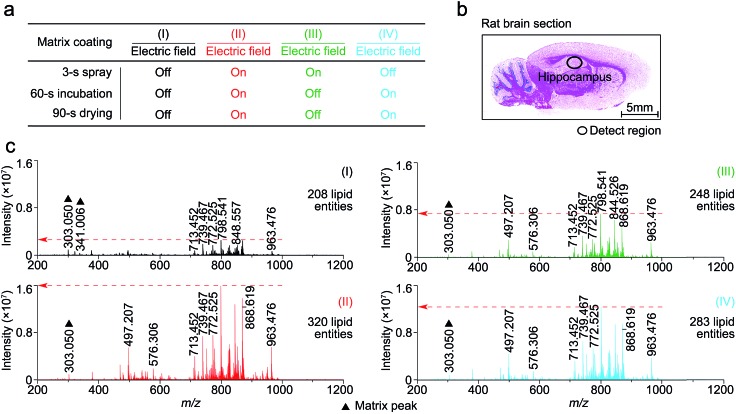
The effect on the MALDI spectra of an applied electric field applied at three different stages of the matrix spray coating process. (a) Experiment design of electric field applied at different steps of the matrix spray coating. (b) H&E stained image of a representative rat brain tissue section. (c) Four mass spectra corresponding to experiments I to IV (a), acquired from the same hippocampus region of the four rat brain tissue sections and using a completely same set of MS operating and data acquisition parameters. The matrix-related ions are labeled “▲”.

To determine at which step(s) in the procedure a static electric field applied during the spray deposition led to enhanced MALDI-MS detection, four experiments, as indicated in Fig. 2a as I to IV, were performed on four consecutive 12 μm rat brain tissue sections (represented by Fig. 2b) sliced from the same rat brain, with and without the electric field applied during the three different steps of each matrix spray cycle. After the matrix coating with quercetin, on-tissue detection was performed by MALDI-FTICR MS using an identical set of MS operating and data acquisition parameters. Fig. 2c shows the four mass spectra, corresponding to the four experiments (I to IV), which were acquired from the same hippocampal region of the four tissue sections. In the supplementary information, Table S1† lists the detected and identified lipid entities and the observed *S*/*N* ± standard derivation for each of the identified lipid entities. In summary, 320, 248, and 283 lipid entities were detected from spectra II to IV, respectively, as compared to only 208 lipid entities detected from spectrum I where the experiment was performed without the use of an electric field during the entire matrix coating process. As can be seen from Table S1,† the *S*/*N*s of all the detected lipids in spectra II to IV were clearly higher than those in spectrum I. Comparison of the four mass spectra indicates that the signal enhancement that results from the application of an electric field during the matrix coating process occurs not only during the spray step but also during the matrix incubation and drying steps.

### MCAEF for rat brain lipid detection in both ion detection modes

**Fig. 3:**
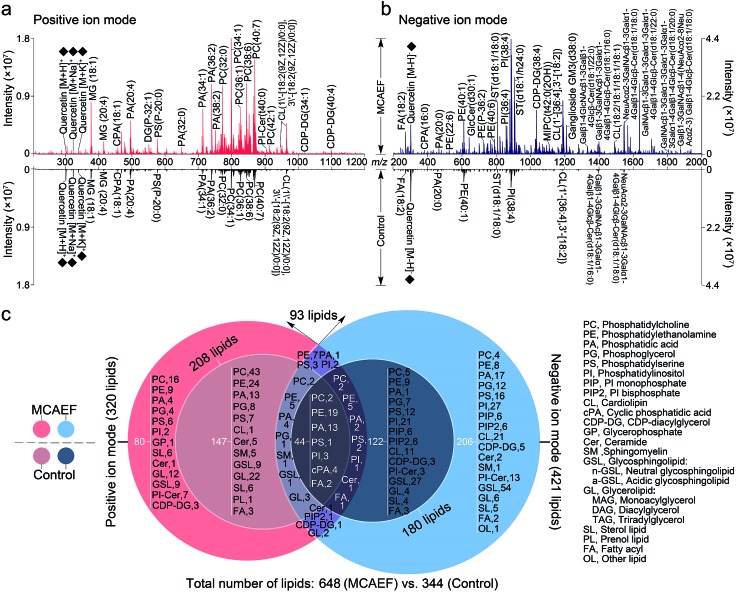
Comparison of lipid detection in rat brain by MALDI-FTICR MS, with and without MCAEF, using quercetin as the MALDI matrix. (a) Positive-ion MALDI mass spectra. (b) Negative-ion MALDI mass spectra. The matrix-related ions are labeled “♦”. (c) Venn diagram showing the classification of identified lipids with positive (red) and negative (blue) ion detection.

We next evaluated whether the applied electric field could also be used for improved compound detection on other tissues and with both positive and negative ion detection by MALDI-MS. Mass spectra acquired from rat brain tissue sections in the positive and negative ion modes, with quercetin as the matrix and FTICR MS detection, with and without MCAEF, are shown in Fig. 3a and b. As shown, MCAEF significantly increased the lipid ion intensities not only in the positive ion mode but also in the negative ion mode. An *E* of 600 V m^–1^ produced a plateau in the normalized *S*/*N*s for rat brain lipid detection in both ion modes, above which no further increase was observed. An average of nearly 5.0- and 3.5-fold ion *S*/*N* increases were observed in the positive and negative ion detection modes, respectively, by comparing the upper (*E* = 600 V m^–1^) and lower (*E* = 0) mass spectra of Fig. 3a and b. Similar to a previous study on porcine adrenal gland imaging by MALDI-FTICR MS,^16^ lipids detected from rat brains in the positive ion mode were mainly observed in a relatively narrow mass range of *m*/*z* 300 to 1000, while the predominant mass range in the negative ion mode for lipid detection was from *m*/*z* 200 to 1800.

In many studies, the identification of some of the imaged lipid species was done by *in situ* MALDI-MS/MS using collision-induced dissociation (CID).^2,31,57^ However, due to the relatively low sensitivity of MALDI-CID on the FTICR instrument used in this study,^15,16,32^ structural confirmation of many of the imaged lipids in tissue sections was performed by LC-MS/MS analysis of the lipid extracts. Other compounds were identified by querying the literature as well as the metabolome database, using the measured accurate masses from the MALDI-FTMS spectra. For compounds assigned on the basis of the metabolome database search only, when more than one candidate was matched, the identities are reported as one of these candidates “or its isomers” (as in the supplementary information, Tables S2 and S3†). It should be mentioned that the assignments of the molecular species in the MALDI-FTMS images by using LC-MS/MS are only tentative, because identical elemental compositions could possibly result from different lipid molecular species. The use of higher-sensitivity MALDI-MS/MS instruments may help to at least partially solve this problem. A total of 648 lipid entities were successfully identified from the mass spectra displayed in the upper part of Fig. 3a and b. The identification was made by querying the metabolome databases based on the accurate MW determination or by using LC-MS/MS using the same procedures as described in our previous studies.^15,16,32^ The identities of these lipids are listed in the supplementary information (Tables S2 and S3†). Fig. 3c shows more detailed information on the classification of these identified lipids. Of the identified lipids, 320 were detected in the positive ion mode and 421 were detected in the negative ion mode. In contrast, only 344 lipids were detected and identified from the mass spectra which were acquired from the tissue sections without MCAEF, shown in the lower parts of Fig. 3a and b. Of the 344 lipids, 208 and 180 lipid entities were identified in the positive and negative ion modes, respectively. Importantly, the total number of lipids that were detected on the rat brain tissue sections showed that MCAEF resulted in an approximately 88% increase in the number of the detected lipids. MCAEF produced a nearly 54% increase (320/208) in the number of detected lipids in the positive ion mode and a 133% increase (421/180) in the negative ion mode. Among these detected lipids, 80 and 206 lipid entities, which respectively belonged to 13 and 18 lipid classes as summarized in Fig. 3c, were *only* detectable in the positive and negative ion modes, respectively, when the electric field (*E* = 600 V m^–1^) was applied during matrix coating. To the best of our knowledge, the use of MCAEF in this experiment has resulted in the largest number of lipids detected by MALDI-MS on rat brain tissue sections reported thus far. As shown in Fig. S4,† PE-Cers and PI-Cers were found to be the most preferentially extracted lipid class, and showed an 8-fold increase in the number of species detected by MALDI when MCAEF was used. The next highest fold-change was for the CDP-DGs, with a 4.3-fold increase in the number of species.

### MCAEF for lipid imaging

To determine whether MCAEF would improve MALDI tissue imaging with the use of different MALDI matrices for the matrix coating, rat brain tissue sections were coated with four different MALDI matrices (quercetin, 2-MBT, dithranol, and 9-AA) which solutions were prepared in different solvents and under different pH values as described in the supplementary information.† The laser raster step size (spatial resolution) was 200 μm (the minimum possible for the 355 nm solid-state Smartbeam Nd:YAG UV laser source) with the laser spot diameters of about 80 μm for lipid imaging.

**Fig. 4:**
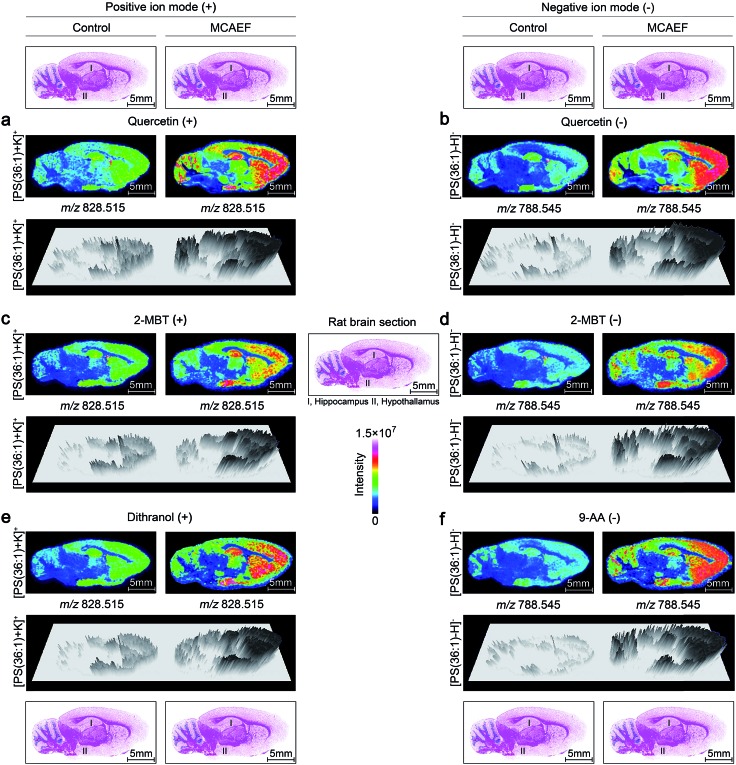
Comparison of lipid signals across sagittal tissue sections of a rat brain, as detected by MALDI-FTICR MS, with and without MCAEF. Four matrices, including quercetin (a and b), 2-MBT (c and d), dithranol (e), and 9-AA (f), were employed for lipid imaging so as to assess the performance of MCAEF. (a, c, and e) Positive-ion lipid MALDI imaging. (b, d, and f) Negative-ion lipid MALDI imaging. For each lipid ion, the left image was from matrix coating without an electric field applied and the right image was from matrix coating with MCAEF. The three-dimensional maps of these lipid ions are included.

Fig. 4a, c and e show the paired images for the lipid [PS(36 : 1) + K]^+^ (*m*/*z* 828.515) using three different MALDI matrices (*i.e.*, quercetin, 2-MBT, and dithranol), with and without the use of an electric field (*E* = 600 V m^–1^) during the matrix coating. Fig. 4b, d and f show the paired images of the same lipid [PS(36 : 1) – H]^–^ (*m*/*z* 788.545) in the negative-ion mode, using three different MALDI matrices (*i.e.*, quercetin, 2-MBT, and 9-AA), with and without the use of an electric field (*E* = 600 V m^–1^) during the matrix coating. As can be seen from this figure, the lipid ion images obtained with MCAEF show higher contrast due to the increased peak intensities, as compared to the corresponding control images, obtained without MCAEF. Taking the regions of hippocampus and hypothalamus of the rat brain as examples, both ions of PS(36 : 1) show distributions with finer visualization with MCAEF. As shown in Fig. S5,† the ion of PI(40 : 8), which was detected mainly in the white matter of the rat brain cerebellum, also shows better images with MCAEF.

**Fig. 5:**
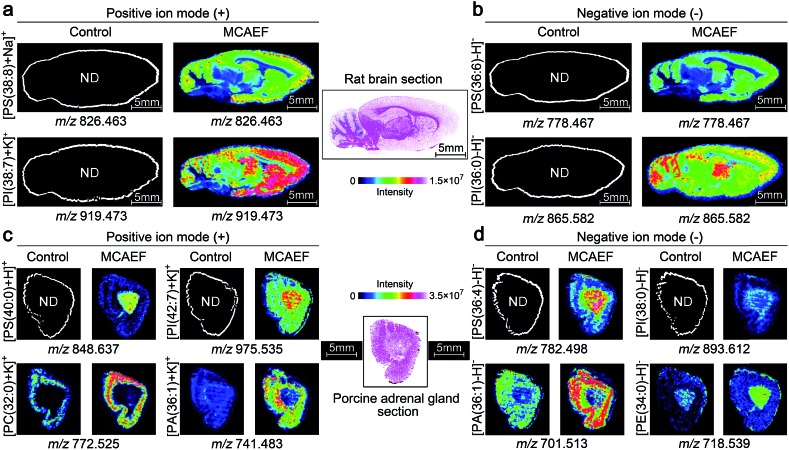
Performance evaluation of MCAEF for lipid MALDI imaging on tissue sections of different mammalian tissues. Comparison of lipid detection on tissue sections of a rat brain (a and b) and a porcine adrenal gland (c and d) with and without MCAEF. “ND” means the molecules were not detected.

Fig. 5a and b show the ionic images of four lipids, including two positive ion detected species, [PS(38 : 8) + Na]^+^ (*m*/*z* 826.463) and [PI(38 : 7) + K]^+^ (*m*/*z* 919.473), and two negative ion detected species, [PS(36 : 6) – H]^–^ (*m*/*z* 778.467) and [PI(36 : 0) – H]^–^ (*m*/*z* 865.582). These four lipids were not detectable on the rat brain tissue sections by MALDI-MS if no MCAEF was applied but were clearly detected after the use of MCAEF. The successful detection of these lipids allowed MALDI imaging of these molecules in the tissue.

We next investigated whether MCAEF could also improve MALDI imaging on tissue sections other than rat brain. 12 μm thick sections of porcine adrenal gland were used for imaging in both ion modes by MALDI-FTICR MS using quercetin as the matrix. Similarly, four lipids, *i.e.*, *m*/*z* 848.637 [PS(40 : 0) + H]^+^ and *m*/*z* 975.535 [PI(42 : 7) + K]^+^, *m*/*z* 782.498 [PS(36 : 4) – H]^–^, and *m*/*z* 893.612 [PI(38 : 0) – H]^–^, which were not detectable in the control (*E* = 0) mass spectrum, were detected in the positive and negative ion mode, respectively, with MCAEF (Fig. 5c and d). Moreover, for those weakly detected lipids in the control spectrum, including *m*/*z* 772.525 [PC(32 : 0) + K]^+^ and *m*/*z* 741.483 [PA(36 : 1) + K]^+^, and *m*/*z* 701.513 [PA(36 : 1) – H]^–^ and *m*/*z* 718.539 [PE(34 : 0) – H]^–^, the image quality of these lipids was significantly improved because of the use of MCAEF which resulted in their finer-visualization patterns in the porcine adrenal gland, as shown in Fig. 5c and d.

These results illustrated that the use of MCAEF resulted in a remarkable enhancement of tissue imaging of lipids in the rat brain and in porcine adrenal glands in both positive and negative ion modes, and was also compatible with the use of different matrices. Considering the different solvents and the different pH values of the four matrix solutions, the improvements of tissue imaging with MCAEF seems to be independent of the composition of the matrix solutions.

### MCAEF for protein imaging

**Fig. 6:**
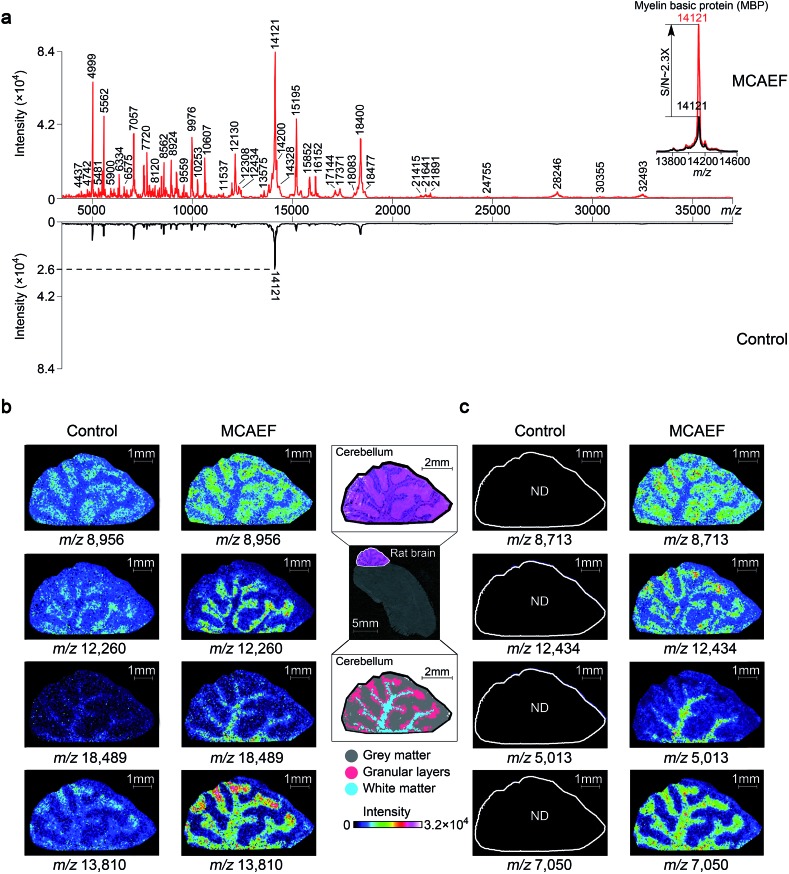
Enhancement of protein imaging using MCAEF. (a) Comparison of MALDI-TOF mass spectra acquired on a rat brain tissue section with (red) and without (black) MCAEF. Sinapinic acid was used as the matrix. (b and c) Comparison of protein images with and without MCAEF. The left control images were from matrix coating without MCAEF and the right images were obtained with MCAEF. “ND” means the molecules were not detected.

To determine if MCAEF also enhanced on-tissue detection and imaging of proteins, we used SA as the matrix to coat 12 μm rat brain tissue sections, with and without MCAEF, for MALDI-TOF MS imaging, with a laser raster step size of 50 μm and a laser spot diameter of *ca.* 50 μm. Fig. 6a shows that the previously optimized *E* (600 V m^–1^) was also suitable for enhanced protein detection in the positive ion mode, and also shows that the intensities and *S*/*N*s of the detected proteins on the mass spectra were greatly increased when MCAEF was used. On average, the use of MCAEF increased the *S*/*N*s of the detected proteins on the tissue sections by a factor of 2 to 4. Taking myelin basic protein at *m*/*z* 12 121 as an example,^58^ MCAEF produced MALDI-TOF MS *S*/*N*s (inset) which increased 2.3 fold. As was the case for lipids, the significantly increased detection sensitivity resulted in a larger number of proteins that were able to be detected in the rat tissue. With MCAEF (*E* = 600 V m^–1^), 232 protein signals were observed from the mass spectra acquired with MCAEF, while only 119 protein signals were detected in the control spectra without MCAEF. The increased detection sensitivity enabled imaging of peptides and proteins across the whole mass detection range, including many higher MW proteins. In the supplementary information, Table S4† lists the observed putative protein signals, although the identities of most of these protein signals remain unknown. Identification of the MALDI imaged proteins may be possible by combining protein extraction, tryptic digestion, and LC-MS/MS, as has been done for other tissues.^14^ However, this was beyond the scope of the current study.

Fig. 6b and c show the effect of MCAEF on the images of proteins detected on the rat brain sections. Four proteins (at *m*/*z* 8956, *m*/*z* 12 260, *m*/*z* 18 489 and *m*/*z* 13 810), which were detectable under both the control (*E* = 0) and the MCAEF (*E* = 600 V m^–1^) conditions, showed finer image visualization with the use of MCAEF. Spatial distributions of these proteins in the grey matter, white matter, and granular layer of the rat brain cerebellum region were more clearly observed because of the higher *S*/*N*s. Fig. 6c shows the images of four small protein signals (*m*/*z* 8713, *m*/*z* 12 434, *m*/*z* 5013, and *m*/*z* 7050). These four proteins were only detectable with the use of MCAEF and were not observable in the control experiment. The images of these eight proteins show distinct distributions in the histological structure of the cerebellum – *i.e.*, these protein species showed different localization in the cerebellum. Proteins represented by *m*/*z* 8956 and *m*/*z* 8713 were observed with higher abundance within the grey matter while the proteins of *m*/*z* 12 260 and *m*/*z* 12 434, and proteins of *m*/*z* 18 489 and *m*/*z* 5013 were uniquely observed in the granular layer and the white matter of the rat brain cerebellum, respectively. Proteins of *m*/*z* 13 810 and *m*/*z* 7050 were found mainly distributed in white matter and granular layers of the cerebellum, while the protein of *m*/*z* 13 810 shows a higher abundance distribution at the end of the white matter and in the granular layers in the rat brain. This experiment showed that MCAEF not only enhances protein detection on tissue by MALDI-MS, but also provides the opportunity to successfully image some proteins that were not previously observable in the MALDI tissue imaging experiments.

## Conclusion

The experimental results presented in this study demonstrate that MCAEF results in increased *S*/*N*s and higher numbers of lipids and proteins detected on tissue by MALDI-MS. MCAEF showed good compatibility not only with different tissue samples but also with different MALDI matrices which were prepared in different solvents with different pH values. The electric field-induced matrix droplet polarization and subsequent on-tissue micro-extraction of the chargeable analytes into the matrix layers is thought to be the mechanism which accounts for MCAEF's improved MALDI-MS detection and imaging. These results indicated that MCAEF has the potential to become a standard practice for enhanced tissue imaging by MALDI-MS because the instrumental set-up is straightforward and it is easy to install and use.

## Conflict of interest

The authors declare no competing financial interests.
