# Supplementary material for: Matrix coating assisted by an electric field (MCAEF) for enhanced tissue imaging by MALDI-MS
Source: Chem Sci. 2014 Sep 16;6(1):729–38. doi: 10.1039/c4sc01850h (PMC5494562; doi:10.1039/c4sc01850h)
Supplement: Supplementary file 1 [file SC-006-C4SC01850H-s001.pdf]

Electronic Supplementary Material (ESI) for Chemical Science.
This journal is © The Royal Society of Chemistry 2014

Electronic Supplementary Information:

Matrix Coating Assisted by an Electric Field (MCAEF) for enhanced tissue imaging by MALDI-MS

Xiaodong Wang,[†] Jun Han,[†] Juncong Yang,[†] Jingxi Pan,[†] and Christoph H. Borchers[*,†,§]

[†]University of Victoria - Genome British Columbia Proteomics Centre, Vancouver Island Technology Park, #3101-4464 Markham St., Victoria, BC V8Z 7X8, Canada

[§]Department of Biochemistry and Microbiology, University of Victoria, Petch Building Room 207, 3800 Finnerty Rd., Victoria, BC V8P 5C2, Canada

***Corresponding author:**

Christoph H. Borchers, Ph.D.

Email: christoph@proteincentre.com

Tel.: (250) 483-3221; **Fax:** (250) 483-3238

EXPERIMENTAL SECTION

Materials and Reagents. Unless otherwise noted, chemical reagents were purchased from Sigma-Aldrich (St. Louris, MO). The "ESI tuning mix" solution was purchased from Agilent Technologies (Santa Clara, CA). Rat liver, rat brain, and porcine adrenal gland specimens were purchased from Pel-Freez Biologicals (Rogers, AR). According to the accompanying sample information sheet, after harvesting, all of the tissue specimens were flash-frozen by slow immersion in liquid nitrogen to avoid shattering. The use of the animal organs involved in this study was in accordance with current requirements of the Canadian Council on Animal Care and was approved by the Ethics Committee of the University of Victoria.

Tissue Sectioning. The frozen tissue samples were sectioned to 12-μm slices in a Microm HM500 cryostat (Waldorf, Germany) at -20 ^{o}C and thaw mounted onto 25 mm x 75 mm conductive ITO coated glass slides obtained from Bruker Daltonics (Bremen, Germany). The slides were then placed under a vacuum of 0.1 psi for 20 min before matrix coating. For protein analysis, the tissue sections were washed in Petri dish twice with 70% ethanol for 30 seconds followed by another wash with 95% ethanol for 15 seconds to remove lipids before vacuum drying and matrix coating.

Histological Staining. Hematoxylin and eosin (H&E) staining was performed based on a previously reported procedure [1] to obtain histological optical images.

Matrix Coating Assisted by an Electric Field (MCAEF). MALDI matrix was coated inside a Bruker Daltonics ImagePrep matrix sprayer (Bremen, Germany) with an electronic sprayer. To apply a static electric field to a tissue section during matrix coating, the ITO-coated conductive slide (where the tissue section was mounted) was used as a positive or negative electrode plate.

Another ITO-coated blank slide was used as the negative or positive electrode plate, and was placed parallel to and above the tissue-mounted ITO slide, 50 mm apart. The conductive sides of the two electrode plates were placed face-to-face. A voltage-adjustable power supply (Model 1672, B&K Precision Corp., Yorba Linda, CA) was used to apply DC voltages to the paired electrode plates through fine metal wires, which were connected to one edge of the conductive side for each of the two slides. The polarity of the tissue-coated slide was dependent on the ion detection mode of the subsequent MALDI-MS analysis. For positive-ion MS detection, the tissue mounted slide was used as the positive electrode plate during matrix coating, while for negative-ion MS detection the tissue mounted slide was the negative electrode plate during matrix coating.

For matrix coating, quercetin was prepared at a concentration of 2.6 mg/mL in 80:20 methanol:water, both containing 0.1% NH_4OH. Dithranol was dissolved in 70:30 acetonitrile (ACN):water, both containing 0.01% trifluoroacetic acid (TFA) to form a saturated matrix solution. 2-mercaptobenzothiazole (2-MBT) was prepared at a concentration of 20 mg/mL in 80:20 methanol:water, both containing 2% formic acid (FA). 9-aminoacridine (9-AA) was prepared at 20 mg/mL in 70:30 ethanol:water (with 0.2% TFA in the final mixture). Sinapinic acid (SA) was prepared at a concentration of 25 mg/mL in 80:20 ACN:water (with 0.2% TFA in the final mixture). The matrix coatings for each of the matrices were composed of a 3-s spray, a 60-s incubation, and a 90-s drying per spray cycle, and thirty cycles were applied to the tissue. The Epson Perfection 4490 Photo Scanner was used for optical images of the tissue section capturing.

MALDI-MS. All lipids were determined using an Apex-Qe 12-Tesla hybrid quadrupole-Fourier transform ion cyclotron resonance (FTICR) mass spectrometer (Bruker Daltonics, Billerica, MA)

equipped with an Apollo dual-mode electrospray ionization (ESI)/matrix-assisted laser desorption/ionization (MALDI) ion source used in previous experiments.[2, 3] The laser source was a 355 nm solid-state Smartbeam Nd:YAG UV laser (Azura Laser AG, Berlin, Germany) operating at 200 Hz. A 1:200 diluted Agilent "ESI tuning mix" solution prepared in 60:40 isopropyl alcohol:water (with 0.1% FA in the final mixture) was used for tuning and calibration of the FTICR instrument by infusing from the ESI side of the ion source at a flow rate of 2 μL/min, so that each MALDI mass spectrum contained the reference mass peaks for internal mass calibration. Mass spectra were acquired over the mass range from 150 to 2000 Da in both the positive and negative ion modes, with broadband detection and a data acquisition size of 1,024 kilobytes per second. MALDI mass spectra were recorded by accumulating ten scans at 100 laser shots per scan in MALDI-MS profiling experiments. For tissue imaging, a 200-μm laser raster step size (the minimum possible for the laser source) was used, and four scans (100 laser shots per scan) were summed per array position (*i.e.*, per pixel). For protein profiling and imaging, the mass spectra were collected on an Ultraflex III MALDI time-of-fight (TOF)/TOF mass spectrometer (Bruker Daltonics, Billerica, MA), which were equipped with a SmartBeam laser and operated at 200 Hz in the positive and linear mode over a mass range of m/z 3000 to 40000. A laser spot diameter of 50 μm and a raster step size of 50 μm were used for protein imaging. Teaching points were generated to ensure the correct positioning of the laser for spectral acquisition by the use of *FlexImaging* 2.1 software (Bruker Daltonics, Billerica, MA). Based on a previous study,[4] the collected mass spectra were baseline corrected and intensity normalized by total ion current. A protein standard mixture in the mass range of *m/z* 5000 to 25000 was used for MALDI-TOF/TOF instrument external calibration, including insulin

([M+H]$^+$, *m/z* 5734.52), ubiquitin I ([M+H]$^+$, *m/z* 8565.76), cytochrome c ([M+H]$^+$, *m/z* 12360.97), myoglobin ([M+H]$^+$, *m/z* 16953.31), trypsinogen ([M+H]$^+$, *m/z* 23982.00).

Data Analysis. Lipid profiling data were viewed and processed using the Bruker *DataAnalysis* 4.0 software. According to previous studies, a customized VBA script was used for batch internal mass calibration, peak de-isotoping, monoisotopic "peak picking", and peak alignment.[2, 3, 5] METLIN[6] and LIPID MAPS[7, 8] metabolome databases were used for match the measured *m/z* values to possible metabolite entities, within an allowable mass error of ±1 ppm. Three ion forms ([M+H]$^+$, [M+Na]$^+$, and [M+K]$^+$) were allowed during database searching in the positive-ion mode; the [M-H]$^-$, [M+Na-2H]$^-$, [M+K-2H]$^-$, and [M+Cl]$^-$ ion forms were allowed during database searching in the negative-ion mode data processing. For protein data analysis, the Bruker *FlexAnalysis* 3.4 software was employed for protein spectra processing and viewing. A mass window of 0.3% and a signal to noise (S/N) ratio of 3 were selected for peak detection. The Bruker *FlexImaging* 2.1 software was used to reconstruct the ion maps of both detected lipids and proteins. The *PDQuest 2-D Analysis* 8.0.1 software (Bio-Rad, Hercules, CA) was used to generate 3D maps.

Lipid Extraction and LC/MS/MS. Total lipids from the same rat brain, which have been subjected to MALDI profiling or imaging, were extracted according to a previously described protocol.[2, 3] Briefly, the rat brain tissue (*ca.* 20 mg) was homogenized in 200 μL of water by a Retsch MM400 mixer mill (Haan, Germany) with the aid of two 5-mm stainless steel balls for 30 s x 2 at a vibration frequency of 30 Hz. Next, 800 μL of a mixed chloroform-methanol (1:3, v/v) solvent was added, followed by another 30-s homogenization step. Then, the tube was centrifuged at 4000 x g and 4 ^{o}C for 20 minutes. The supernatants were collected and mixed with 250 μL of chloroform and 100 µL of water. After a short vortex mixing (~15 s) and re-

centrifugation at 10600 x g for 5 min, the lower organic phase in each tube was carefully transferred to a new tube using a 200-μL gel loading pipette tip, and then dried in a Savant SPD1010 speed-vacuum concentrator (Thermo Electron Corporation, Waltham, MA) and stored at -80 ^{o}C until used.

A Waters ACQUITY UPLC system coupled to a Waters Synapt HDMS quadrupole-TOF (Q-TOF) mass spectrometer (Beverly, MA) was used as a complementary technique for structural confirmation of most of the detected mass-matched lipid compounds. Briefly, the dried lipid extract residues were re-dissolved in 100 μL of chloroform and 8 μL aliquots were injected onto a Waters Atlantis® HILIC silica column (3 μm particle size, 4.6 mm i.d. x150 mm; Beverly, MA) for different lipid specie separations based on their head groups.[9-11] LC/MS data were collected in both positive and negative ESI modes, with respective injections. MS/MS experiments were conducted using collision-induced dissociation (CID) applied to the trapping collision cell of the Q-TOF instrument. The optimal collision voltages were selected to obtain abundant product ions. UPLC-MS data were processed by the Waters *MassLynx* software (version 4.1) suite. Lipid identities were assigned by combining mass-matched metabolome database searching against the METLIN database with MS/MS spectral searching against the standard MS/MS libraries in the METLIN,[6, 12] HMDB,[13, 14] or LIPID MAPS[15, 16] databases.

Supplementary Information -- Figures and Figure Legends

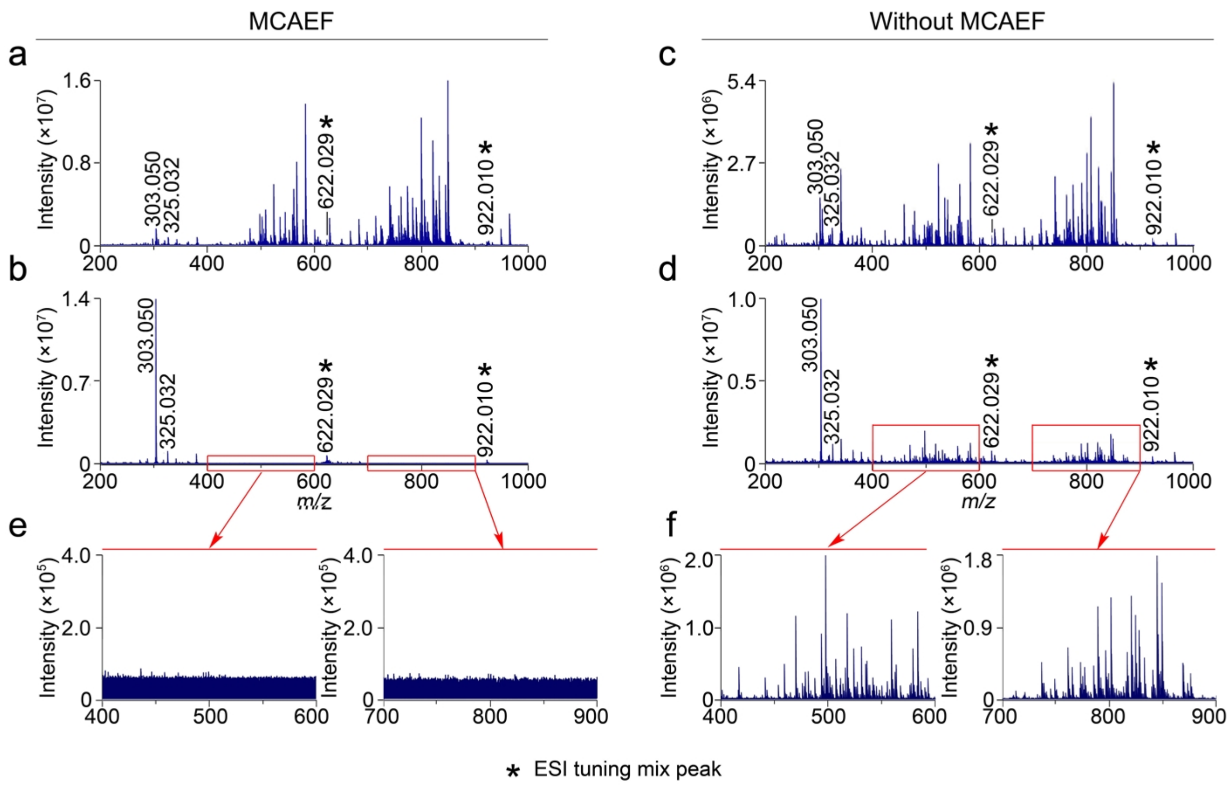

Supplementary Information Figure S1. Evaluation of the extent of electric-field driven micro-extraction of analytes from the tissue section. These MALDI mass spectra were acquired from a rat liver tissue section *with* MCAEF during quercetin matrix coating (**a**), and after matrix removal with methanol followed by matrix re-coating (**b**); and from another rat liver tissue section *without* MCAEF during quercetin matrix coating (**c**) and after matrix removal with methanol followed by matrix re-coating (**d**). For (**b**) and (**d**), no MCAEF was used during matrix re-coating. (**e**) The zoomed-in (panel b) mass spectrum with a mass window of 200 Da for *m/z* 400-600 and 700-900. (**f**) The zoom-in (panel **d**) mass spectra with a mass window of 200 Da for *m/z* 400-600 and 700-900. The ions of *m/z* 303.050 and 325.032 are matrix (quercetin) peaks. The ESI tuning-mix peak is labeled with an asterisk "*".

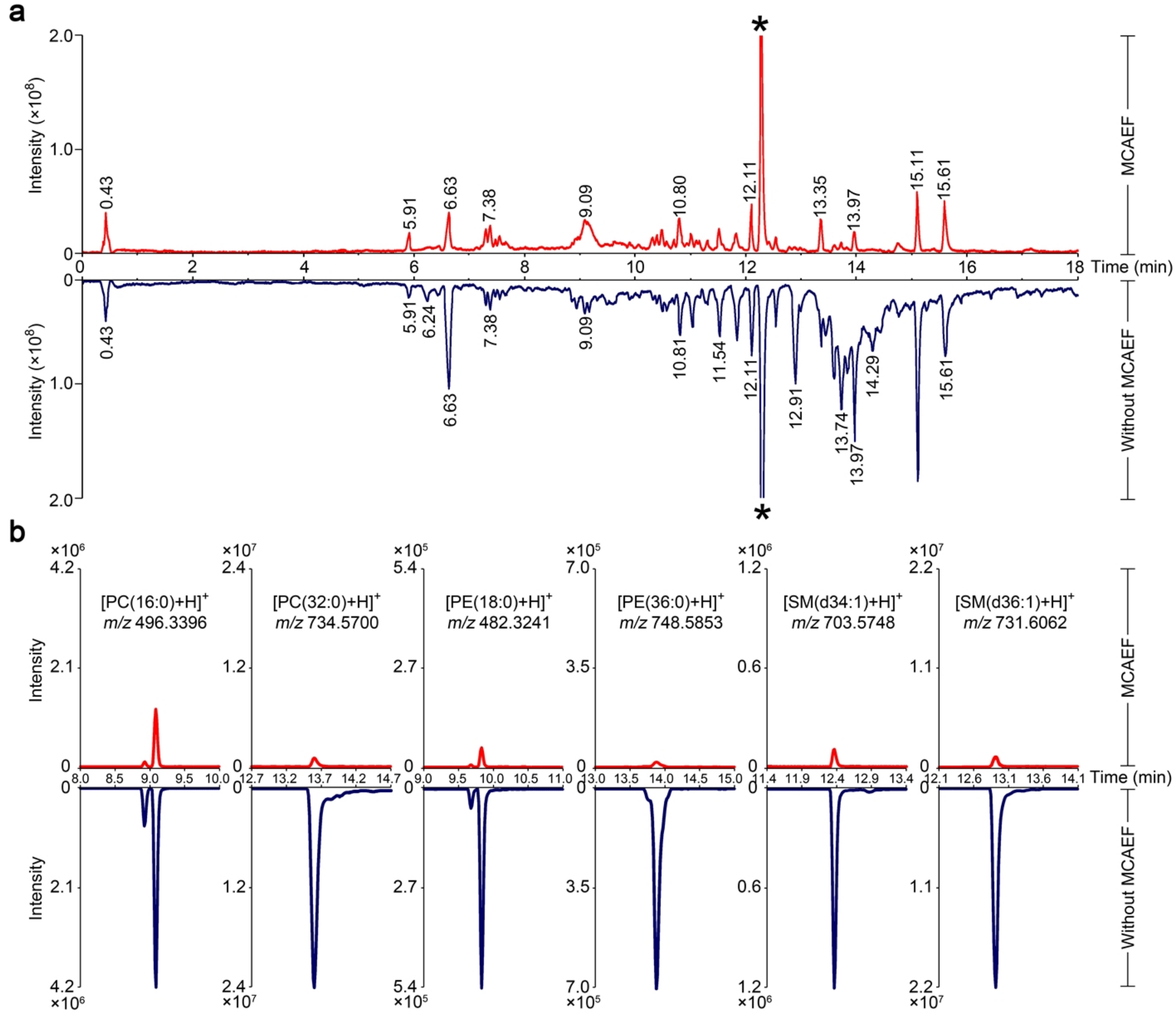

Supplementary Information Figure S2. UPLC-MS analysis of extracted lipids from rat brain tissue sections. (**a**) Total ion current chromatograms of lipids extracted from rat liver tissue sections *with* MCAEF (red) and *without* MCAEF (blue) during quercetin matrix coating. The lipid extractions were carried out after matrix removal with methanol. (**b**) The extracted ion chromatograms of *m/z* 496.3396 [PC(16:0)+H]$^+$, *m/z* 734.5700 [PC(32:0)+H]$^+$, *m/z* 482.3241 [PE(18:0)+H]$^+$, *m/z* 748.5853 [PE(36:0)+H]$^+$, *m/z* 703.5748 [SM(d34:1)+H]$^+$, and *m/z* 731.6062 [SM(d36:1)+H]$^+$. The peak at *m/z* 338.3414, labeled with an asterisk "*" in (**a**), is a general contaminant from a plasticizer.

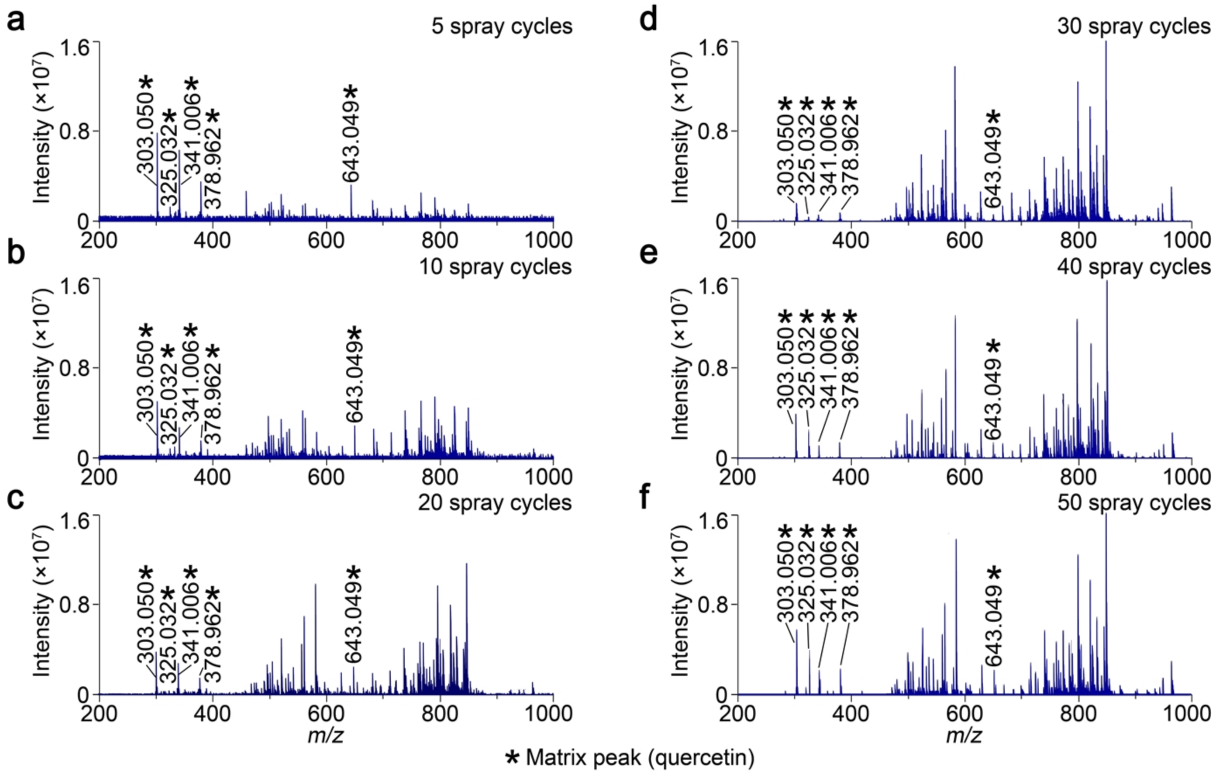

Supplementary Information Figure S3. Optimization of coated matrix thickness for lipid detection on rat liver tissue section.

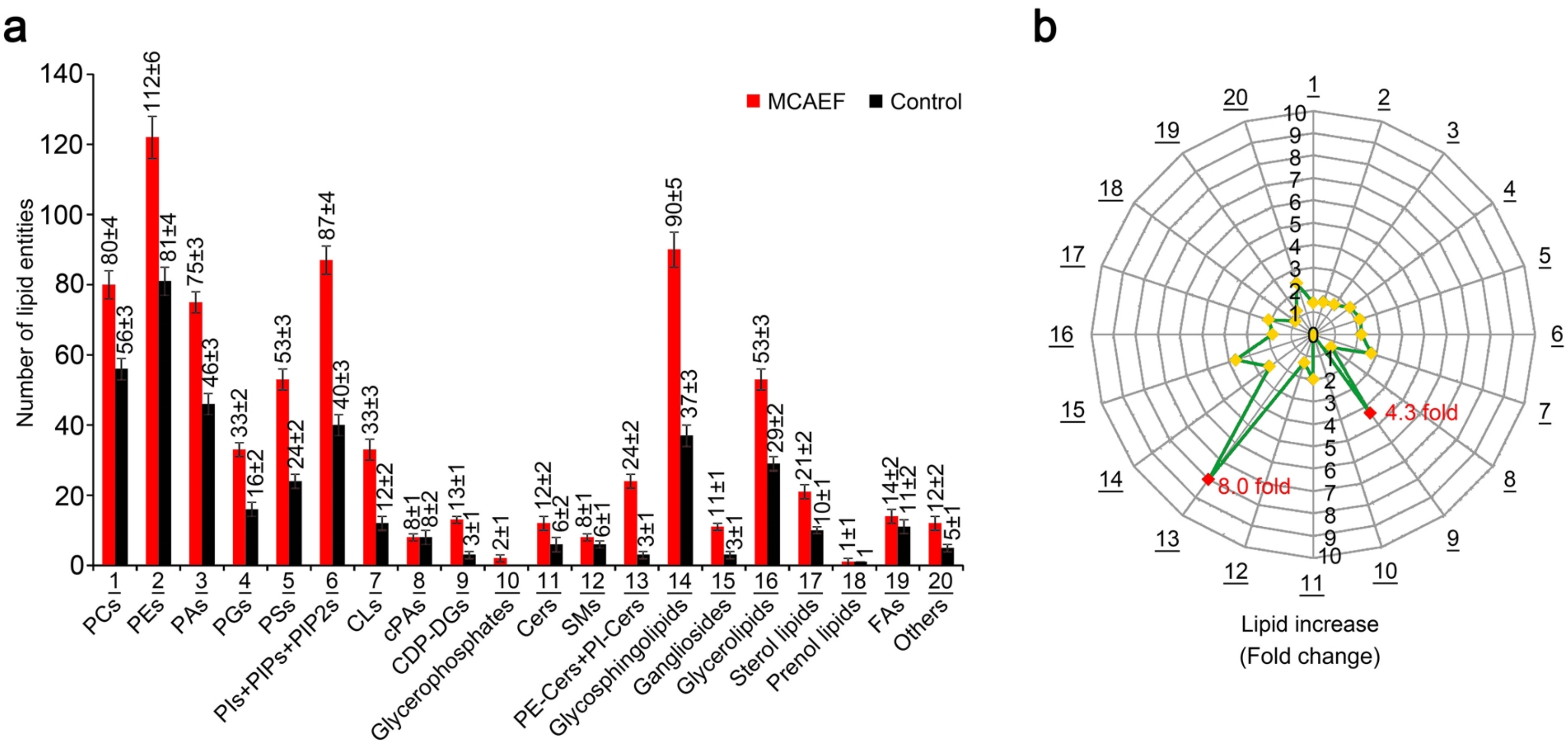

Supplementary Information Figure S4. Preference evaluation of MCAEF for detection of different lipid classes. (**a**) Bar graphs showing the summed numbers of lipid species detected from a rat brain tissue section by MALDI-FTICR MS, with and without MCAEF. (**b**) Radar graph showing the fold changes of the detected different lipid classes, with and without MCAEF.

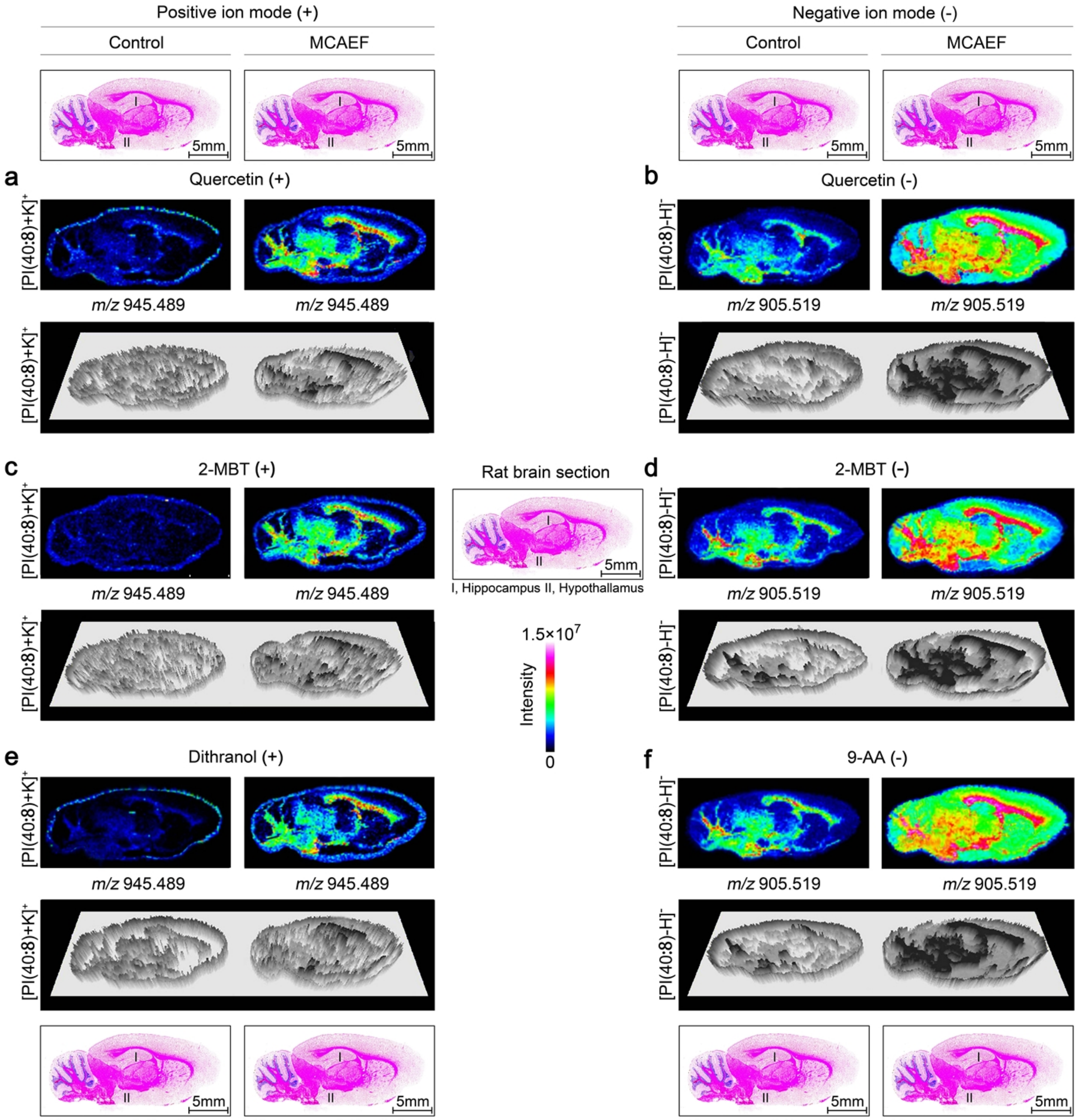

Supplementary Information Figure S5. Comparison of PI(40:8) signals across sagittal tissue sections of a rat brain, as detected by MALDI-FTICR MS, *with* and *without* MCAEF. Four matrices, including quercetin (**a**, **b**), 2-MBT (**c**, **d**), dithranol (**e**), and 9-AA (**f**), were employed for lipid imaging so as to assess the performance of MCAEF. For each lipid ion, the left image was from matrix coating without an electric field applied and the right image was from matrix coating with MCAEF.

Supplementary Information Table S1. Comparison of lipid detection by MALDI-FTICR MS from the hippocampus region of four rat brain tissue sections *with* and *without* electric field applied during the three different steps of each matrix spray cycle.

**Classification**	**Electric field (Measured *m/z*)**				**Calculated *m/z***	**Electric field (Average *S/N*, n=3)**				**Assignment**		
**Matrix coating**	**I**	**II**	**III**	**IV**		**I**	**II**	**III**	**IV**	Ion form	Compound	Molecular formula
**Spray (3-s)**	Off	On	On	Off		Off	On	On	Off			
**Incubation (60-s)**	Off	On	Off	On		Off	On	Off	On			
**Drying (90-s)**	Off	On	Off	On		Off	On	Off	On			
**Glycerophospholipids**												
Phosphatidylcholines (PCs)												
	478.32921	478.32944	478.32928	478.32951	478.32920	139.9 ±18.6	383.6 ±13.5	204.0 ±15.1	271.2 ±16.9	$[M+H]^+$	PC(O-16:2)	$C_{24}H_{48}NO_6P$
	500.31090	500.31142	500.31066	500.31203	500.31115	3.7 ±2.1	11.2 ±2.4	5.5 ±2.0	6.4 ±3.6	$[M+Na]^+$		
	516.28499	516.28529	516.28541	516.28563	516.28508	22.4 ±3.7	49.4 ±4.4	24.5 ±5.2	37.9 ±4.3	$[M+K]^+$		
	-	502.32663	-	-	502.32680	-	13.9 ±3.5	-	-	$[M+Na]^+$	PC(O-16:1)	$C_{24}H_{50}NO_6P$
	-	518.30086	-	518.30122	518.30073	-	15.2 ±4.8	-	11.4 ±7.1	$[M+K]^+$		
	-	496.33852	-	496.33830	496.33977	-	13.2 ±4.6	-	8.7 ±5.3	$[M+H]^+$	PC(16:0)	$C_{24}H_{50}NO_7P$
	534.29559	534.29568	534.29588	534.29580	534.29565	25.4 ±5.9	57.6 ±4.8	30.1 ±8.3	41.6 ±7.5	$[M+K]^+$		
	-	504.34245	504.34267	504.34272	504.34245	-	12.2 ±5.6	8.5 ±6.1	10.4 ±5.9	$[M+Na]^+$	PC(O-16:0)	$C_{24}H_{52}NO_6P$
	516.30887	516.30846	516.30845	516.30886	516.30847	12.4 ±5.5	49.4 ±6.1	24.5 ±8.1	37.9 ±6.9	$[M+H]^+$	PC(18:4)	$C_{26}H_{46}NO_7P$
	-	518.32420	-	518.32407	518.32412	-	15.2 ±5.8	-	12.8 ±7.8	$[M+H]^+$	PC(18:3)	$C_{26}H_{48}NO_7P$
	506.36056	506.36059	506.36043	506.36065	506.36050	77.2 ±11.0	132.9 ±8.1	91.8 ±7.9	106.9 ±9.3	$[M+H]^+$	PC(P-18:1)	$C_{26}H_{52}NO_6P$
	-	528.34252	-	528.34269	528.34245	-	21.4 ±5.1	-	14.7 ±3.7	$[M+Na]^+$	PC(O-18:2)	$C_{26}H_{52}NO_6P$
	544.31639	544.31641	544.31718	544.31689	544.31638	5.7 ±3.7	21.7 ±6.4	12.8 ±4.6	18.5 ±5.8	$[M+K]^+$		
	-	522.35540	522.53496	522.53563	522.35542	-	12.1	3.7	10.7	$[M+H]^+$	PC(18:1)	$C_{26}H_{52}NO_7P$

Classification		Electric field (Measured *m/z*)				Calculated *m/z*		Electric field (Average *S/N*, n=3)				Assignment		
	**Matrix coating**	**I**	**II**	**III**	**IV**		**Matrix coating**	**I**	**II**	**III**	**IV**	Ion form	Compound	Molecular formula
	**Spray (3-s)**	Off	On	On	Off		**Spray (3-s)**	Off	On	On	Off			
	**Incubation (60-s)**	Off	On	Off	On		**Incubation (60-s)**	Off	On	Off	On			
	**Drying (90-s)**	Off	On	Off	On		**Drying (90-s)**	Off	On	Off	On			
									±3.1	±2.2	±5.2			
		560.31123	560.31133	560.31151	560.31156	560.31130		9.5 ±5.7	24.7 ±3.9	11.9 ±6.4	18.8 ±5.8	$[M+K]^+$		
		524.37117	524.37105	524.37130	524.37093	524.37107		8.3 ±7.1	34.4 ±6.4	18.4 ±6.3	27.7 ±5.5	$[M+H]^+$	PC(18:0)	$C_{26}H_{54}NO_7P$
		562.32677	562.32705	562.32757	562.32771	562.32695		8.6 ±4.7	23.1 ±3.7	15.8 ±5.9	18.1 ±4.5	$[M+K]^+$		
		544.33970	544.33976	544.33927	544.33968	544.33977		6.3 ±3.2	15.0 ±5.6	7.7 ±2.7	8.3 ±3.2	$[M+H]^+$	PC(20:4)	$C_{28}H_{50}NO_7P$
		-	582.29573	-	-	582.29565		-	5.5 ±3.3	-	-	$[M+K]^+$		
		-	546.35542	-	-	546.35542		-	5.1 ±2.6	-	-	$[M+H]^+$	PC(20:3)	$C_{28}H_{52}NO_7P$
		548.37142	548.37104	548.37140	548.37095	548.37107		6.9 ±4.6	11.7 ±3.9	7.3 ±5.1	8.2 ±4.7	$[M+H]^+$	PC(20:2)	$C_{28}H_{54}NO_7P$
		586.32713	586.32691	586.32703	586.32704	586.32695		9.2 ±3.9	19.1 ±5.3	10.3 ±4.6	14.5 ±3.8	$[M+K]^+$		
		602.32227	602.32175	602.32157	602.32169	602.32186		5.1 ±3.6	14.1 ±6.1	5.4 ±3.1	9.3 ±4.6	$[M+K]^+$	PC(20:1)	$C_{28}H_{54}NO_8P$
		604.33764	604.33754	604.33725	604.33788	604.33751		7.3 ±5.4	20.7 ±7.6	9.5 ±6.3	16.1 5.6	$[M+K]^+$	PC(20:0)	$C_{28}H_{56}NO_8P$
		606.29527	606.29571	606.29593	606.29558	606.29565		24.3 ±8.2	53.8 ±7.1	30.0 ±7.9	36.7 ±8.4	$[M+K]^+$	PC(22:6)	$C_{30}H_{50}NO_7P$
		-	608.31099	-	608.31167	608.31130		-	10.3 ±4.8	-	7.4 ±5.4	$[M+K]^+$	LysoPC(22:5)	$C_{30}H_{52}NO_7P$
		610.32706	610.32697	610.32657	610.32668	610.32695		9.0 ±5.2	18.9 ±6.1	13.2 ±6.7	17.2 5.8	$[M+K]^+$	PC(22:4)	$C_{30}H_{54}NO_7P$
		614.35835	614.35824	614.35851	614.35816	614.35825		7.0 ±3.5	15.3 ±4.9	9.1 ±3.8	10.8 ±5.1	$[M+K]^+$	PC(22:2)	$C_{30}H_{58}NO_7P$
		616.37398	616.37392	616.37386	616.37396	616.37390		7.4 ±3.1	16.3 ±5.8	8.3 ±3.3	13.6 ±6.4	$[M+K]^+$	PC(22:1)	$C_{30}H_{60}NO_7P$
		618.38967	618.38953	618.38966	618.38985	618.38955		5.5 ±3.4	17.8 ±5.8	9.2 ±4.1	13.6 ±5.3	$[M+K]^+$	PC(22:0)	$C_{30}H_{62}NO_7P$
		644.40537	644.40524	644.40528	644.40540	644.40520		5.8 ±3.6	13.7 ±4.6	8.1 ±3.8	10.1 ±3.3	$[M+K]^+$	LysoPC(24:1)	$C_{32}H_{64}NO_7P$
		646.42079	646.42097	646.42048	646.42042	646.42085		6.1	13.7	8.9	11.1	$[M+K]^+$	PC(24:0)	$C_{32}H_{66}NO_7P$

Classification	Electric field (Measured *m/z*)				Calculated *m/z*	Electric field (Average *S/N*, n=3)				Assignment		
	Matrix coating					Matrix coating				Ion form	Compound	Molecular formula
	I	II	III	IV		I	II	III	IV			
	Spray (3-s)					Spray (3-s)						
	Off	On	On	Off		Off	On	On	Off			
	Incubation (60-s)					Incubation (60-s)						
	Off	On	Off	On		Off	On	Off	On			
	Drying (90-s)					Drying (90-s)						
	Off	On	Off	On		Off	On	Off	On			
						±4.2	±4.3	±3.4	±4.6			
	648.43664	648.43652	648.43663	648.43635	648.43650	5.6 ±3.0	18.4 ±4.9	10.7 ±3.7	16.6 ±5.1	$[M+K]^+$	LysoPC(26:1)	$C_{32}H_{68}NO_7P$
	650.45234	650.45217	650.45177	650.45262	650.45215	5.0 ±2.6	14.9 ±3.4	8.9 ±3.7	11.4 ±4.8	$[M+K]^+$	LysoPC(26:0)	$C_{32}H_{70}NO_7P$
	704.52246	704.52253	704.52289	704.52249	704.52248	19.1 ±5.3	47.2 ±8.4	25.6 ±6.8	33.5 ±7.6	$[M+H]^+$	PC(30:1)	$C_{38}H_{74}NO_8P$
	744.49457	744.49403	744.49418	744.49469	744.49401	6.6 ±4.3	27.4 ±6.8	16.7 ±3.8	20.3 ±5.9	$[M+K]^+$	PC(30:0)	$C_{38}H_{76}NO_8P$
	766.47811	766.47833	766.47816	766.47839	766.47836	9.1 ±5.8	20.5 ±6.7	15.9 ±5.4	19.9 ±6.1	$[M+K]^+$	PC(32:3)	$C_{40}H_{74}NO_8P$
	770.50981	770.51961	770.51028	770.51021	770.50966	10.2 ±6.4	27.6 ±7.2	20.4 ±6.6	24.3 ±7.0	$[M+K]^+$	PC(32:1)	$C_{40}H_{78}NO_8P$
	734.56974	734.57001	734.56907	734.56950	734.56943	5.0 ±3.7	14.9 ±4.2	8.3 ±4.6	12.3 ±6.1	$[M+H]^+$		
	756.55161	756.55138	756.55135	756.55167	756.55138	20.2 ±6.1	50.2 ±5.4	39.5 ±6.7	42.7 ±5.9	$[M+Na]^+$	PC(32:0)	$C_{40}H_{80}NO_8P$
	772.52537	772.52533	772.52518	775.52511	772.52531	181.4 ±12.1	415.2 ±15.7	282.7 ±13.7	325.5 ±16.4	$[M+K]^+$		
	790.47818	790.47837	790.47825	790.47820	790.47836	6.4 ±3.2	23.5 ±5.5	12.3 ±4.5	22.4 ±5.6	$[M+K]^+$	PC(34:5)	$C_{42}H_{74}NO_8P$
	792.49398	792.49404	792.49442	792.49458	792.49401	6.3 ±3.4	14.8 ±5.5	9.4 ±5.1	13.2 ±4.4	$[M+K]^+$	PC(34:4)	$C_{42}H_{76}NO_8P$
	-	794.50967	-	794.50971	794.50966	-	14.2 ±4.1	-	12.7 ±5.0	$[M+K]^+$	PC(34:3)	$C_{42}H_{78}NO_8P$
	-	796.52530	796.52567	796.52576	796.52531	-	16.1 ±5.1	9.4 ±5.9	13.4 ±6.3	$[M+K]^+$	PC(34:2)	$C_{42}H_{80}NO_8P$
	760.58524	760.58505	760.58506	760.58503	760.58508	5.3 ±3.7	15.7 ±4.6	8.7 ±4.1	15.4 ±5.3	$[M+H]^+$		
	782.56776	782.56699	782.56710	782.56734	782.56703	27.4 ±5.4	77.9 ±8.1	39.6 ±6.3	73.2 ±7.2	$[M+Na]^+$	PC(34:1)	$C_{42}H_{82}NO_8P$
	798.54057	798.54082	798.54052	798.54069	798.54096	188.8 ±16.3	683.8 ±18.0	350.2 ±13.4	595.8 ±16.3	$[M+K]^+$		
	-	762.60067	-	762.60111	762.60073	-	29.6 ±5.6	-	23.7 ±6.4	$[M+H]^+$	PC(34:0)	$C_{42}H_{84}NO_8P$
	-	784.58272	784.58291	784.58283	784.58268	-	9.3	6.2	8.6	$[M+Na]^+$		

Classification	Electric field (Measured *m/z*)					Calculated *m/z*	Electric field (<u>Average *S/N*, n=3)</u>					Assignment		
	**Matrix coating**	**I**	**II**	**III**	**IV**		**Matrix coating**	**I**	**II**	**III**	**IV**	Ion form	Compound	Molecular formula
	**Spray (3-s)**	Off	On	On	Off		**Spray (3-s)**	Off	On	On	Off			
	**Incubation (60-s)**	Off	On	Off	On		**Incubation (60-s)**	Off	On	Off	On			
	**Drying (90-s)**	Off	On	Off	On		**Drying (90-s)**	Off	On	Off	On			
									±3.8	±2.8	±4.1			
		-	800.55661	800.55630	800.55657	800.55661		-	84.3 ±10.6	63.4 ±8.4	70.6 ±9.5	$[M+K]^+$	PC(36:4)	$C_{44}H_{80}NO_8P$
		-	804.55132	-	804.55126	804.55138		-	20.2 ±5.1	-	18.9 ±6.4	$[M+Na]^+$		
		820.52528	820.52534	820.52513	820.52528	820.52531		37.3 ±6.4	159.0 ±13.5	86.3 ±9.6	126.3 ±12.8	$[M+K]^+$		
		-	822.54093	-	822.54072	822.54096		-	27.7 ±5.8	-	15.7 ±6.1	$[M+K]^+$	PC(36:3)	$C_{44}H_{82}NO_8P$
		792.56663	792.56679	792.56656	792.56654	792.56678		5.1 ±3.3	14.8 ±5.9	8.4 ±4.2	13.0 ±4.6	$[M+K]^+$	1-hexadecanyl-2-(8-[3]-ladderane-octanyl)-sn-glycerophospho choline	$C_{44}H_{84}NO_6P$
		808.58242	808.58259	808.58282	808.58298	808.58268		6.3 ±3.7	15.3 ±4.8	8.6 ±5.3	13.7 ±6.6	$[M+Na]^+$	PC(36:2)	$C_{44}H_{84}NO_8P$
		824.55618	824.55657	824.55661	824.55654	824.55661		36.0 ±5.7	86.8 ±8.3	57.8 ±6.6	82.1 ±5.9	$[M+K]^+$		
		-	810.57737	810.57742	810.57762	810.57735		-	15.1 ±5.0	7.8 ±4.7	12.9 ±5.5	$[M+K]^+$	PC(P-36:1)	$C_{44}H_{86}NO_7P$
		-	788.61632	788.61684	788.61601	788.61638		-	9.6 ±4.1	5.6 ±3.4	8.4 ±4.6	$[M+H]^+$	PC(36:1)	$C_{44}H_{86}NO_8P$
		826.57220	826.57220	826.57225	826.57219	826.57226		97.1 ±12.5	361.4 ±20.1	177.5 ±15.2	274.8 ±18.6	$[M+K]^+$		
		828.58806	828.58799	828.58789	828.58777	828.58791		16.3 ±6.4	53.8 ±7.2	33.8 ±6.8	41.0 ±6.5	$[M+K]^+$	PC(36:0)	$C_{44}H_{88}NO_8P$
		786.54336	786.54324	786.54326	786.54346	786.54322		6.5 ±3.7	16.9 ±5.6	9.7 ±4.3	13.4 ±5.8	$[M+H]^+$	1-(6-[5]-ladderane-hexanoyl)-2-(8-[3]-ladderane-octanyl)-sn-glycerophospho choline	$C_{46}H_{76}NO_7P$
		844.52541	844.52542	844.52537	844.52539	844.52531		17.7	74.3	40.9	58.2	$[M+K]^+$	PC(38:6)	$C_{46}H_{80}NO_8P$

Classification		Electric field (Measured *m/z*)				Calculated *m/z*		Electric field (Average *S/N*, n=3)				Assignment		
	**Matrix coating**	**I**	**II**	**III**	**IV**		**Matrix coating**	**I**	**II**	**III**	**IV**	Ion form	Compound	Molecular formula
	**Spray (3-s)**	Off	On	On	Off		**Spray (3-s)**	Off	On	On	Off			
	**Incubation (60-s)**	Off	On	Off	On		**Incubation (60-s)**	Off	On	Off	On			
	**Drying (90-s)**	Off	On	Off	On		**Drying (90-s)**	Off	On	Off	On			
								±7.2	±11.2	±8.6	±8.1			
		846.54121	846.54098	846.54089	846.54129	846.54096		14.2 ±6.5	54.7 ±8.3	31.5 ±7.1	45.5 ±7.7	$[M+K]^+$	PC(38:5)	$C_{46}H_{82}NO_8P$
		810.60045	810.60095	810.60064	810.60041	810.60073		9.1 ±5.1	28.3 ±6.8	16.8 ±4.4	26.2 ±5.3	$[M+H]^+$		
		832.58284	832.58253	832.58254	832.58241	832.58268		11.4 ±5.5	20.4 ±6.3	17.4 ±6.0	18.5 ±6.1	$[M+Na]^+$	PC(38:4)	$C_{46}H_{84}NO_8P$
		848.55723	848.55662	848.55674	848.55693	848.55661		165.5 ±15.2	885.3 ±25.5	516.1 ±19.4	714.3 ±23.7	$[M+K]^+$		
		850.57224	850.57227	850.57236	850.57215	850.57226		10.3 ±3.6	27.4 ±6.4	18.5 ±4.2	23.6 ±5.8	$[M+K]^+$	PC(38:3)	$C_{46}H_{86}NO_8P$
		854.60387	854.60371	854.60345	854.60347	854.60356		6.2 ±3.4	23.4 ±5.7	15.7 ±5.5	20.1 ±5.6	$[M+K]^+$	PC(38:1)	$C_{46}H_{90}NO_8P$
		-	840.62426	840.62452	840.62411	840.62430		-	15.7 ±5.4	7.5 ±4.4	10.3 ±6.0	$M+K]^+$	PC(P-38:0)	$C_{46}H_{92}NO_7P$
		856.61947	856.61935	856.61928	856.61948	856.61921		11.8 ±4.2	39.1 ±7.0	31.2 ±6.4	35.5 ±6.6	$M+K]^+$	PC(38:0)	$C_{46}H_{92}NO_8P$
		-	864.49419	-	-	864.49401		-	12.0 ±4.0	-	-	$[M+K]^+$	PC(40:10)	$C_{48}H_{76}NO_8P$
		-	866.50959	-	866.50955	866.50966		-	12.5 4.3	-	10.3 ±4.0	$[M+K]^+$	PC(40:9)	$C_{48}H_{78}NO_8P$
		-	852.53071	-	-	852.53040		-	15.2 ±5.0	-	-	$[M+K]^+$	1-(8-[5]-ladderane-octanoyl)-2-(8-[3]-ladderane-octanyl)-sn-glycerophosphocholine	$C_{48}H_{80}NO_7P$
		870.54121	870.54087	870.54113	870.54091	870.54096		6.4 ±3.1	33.2 ±6.5	25.3 ±4.7	30.5 ±6.1	$[M+K]^+$	PC(40:7)	$C_{48}H_{82}NO_8P$
		856.58264	856.58277	856.58257	586.58275	856.58268		6.6 ±3.7	19.9 ±5.0	12.1 ±4.4	15.7 ±5.1	$[M+Na]^+$	PC(40:6)	$C_{48}H_{84}NO_8P$
		872.55660	872.55655	872.55644	872.55652	872.55661		23.5	120.2	89.3	118.0	$[M+K]^+$		

Classification	Matrix coating	I	II	III	IV	Calculated *m/z*	Matrix coating	I	II	III	IV	Ion form	Compound	Molecular formula
	**Electric field (Measured *m/z*)**						**Electric field (Average *S/N*, n=3)**					**Assignment**		
	**Spray (3-s)**	Off	On	On	Off		**Spray (3-s)**	Off	On	On	Off			
	**Incubation (60-s)**	Off	On	Off	On		**Incubation (60-s)**	Off	On	Off	On			
	**Drying (90-s)**	Off	On	Off	On		**Drying (90-s)**	Off	On	Off	On			
								±5.2	±13.6	±10.6	±14.1			
		874.57235	874.57211	874.57248	874.57213	874.57226		17.9 ±5.3	52.1 ±8.8	32.6 ±7.1	40.3 ±7.6	$[M+K]^+$	PC(40:5)	$C_{48}H_{86}NO_8P$
		876.58740	876.58769	876.58757	876.58750	876.58791		26.0 ±5.5	60.9 ±13.4	42.6 ±7.6	55.8 ±8.3	$[M+K]^+$	PC(40:4)	$C_{48}H_{88}NO_8P$
		-	880.61923	-	880.61956	880.61921		-	10.6 ±4.3	-	8.3 ±4.0	$[M+K]^+$	PC(40:2)	$C_{48}H_{92}NO_8P$
		882.63506	882.63493	882.63498	882.63497	882.63486		7.2 ±3.8	20.3 ±5.3	16.8 ±5.1	18.9 ±5.0	$[M+K]^+$	PC(40:1)	$C_{48}H_{94}NO_8P$
		906.63497	906.63475	906.63490	906.63489	906.63486		6.2 ±3.3	24.6 ±5.6	18.9 ±4.9	21.3 ±5.3	$[M+K]^+$	PC(42:3)	$C_{50}H_{94}NO_8P$
		-	908.65063	908.65075	908.65042	908.65051		-	14.5 ±4.4	10.2 ±4.2	12.9 ±4.3	$[M+K]^+$	PC(42:2)	$C_{50}H_{96}NO_8P$
		910.66627	910.66619	910.66625	910.66611	910.66616		6.7 ±3.4	22.2 ±5.5	18.9 ±5.1	20.3 ±5.3	$[M+K]^+$	PC(42:1)	$C_{50}H_{98}NO_8P$
		-	936.68127	-	-	936.68181		-	8.2 ±5.3	-	-	$[M+K]^+$	PC(44:2)	$C_{52}H_{100}NO_8P$
		-	956.65043	-	-	956.65051		-	6.7 ±3.3	-	-	$[M+K]^+$	PC(46:6)	$C_{54}H_{96}NO_8P$
Phosphatidylethanolamines (PEs)														
		476.25382	476.25387	476.25368	476.25372	476.25378		5.0 ±2.1	23.6 ±5.6	14.1 ±4.4	19.3 ±5.1	$[M+K]^+$	PE(P-16:0)	$C_{21}H_{44}NO_6P$
		490.23316	490.23307	490.23309	490.23304	490.23305		5.1 ±2.2	10.2 ±4.2	7.9 ±4.0	8.8 ±4.1	$[M+K]^+$	PE(16:1)	$C_{21}H_{42}NO_7P$
		-	492.24870	-	492.24864	492.24870		-	8.7 ±4.0	-	8.3 ±4.1	$[M+K]^+$	PE(16:0)	$C_{21}H_{44}NO_7P$
		514.23316	514.23314	514.23292	514.23296	514.23305		5.2 ±2.3	14.4 ±4.5	9.6 ±4.2	13.4 ±4.4	$[M+K]^+$	PE(18:3)	$C_{23}H_{42}NO_7P$
		516.24877	516.24867	516.24866	516.24880	516.24870		5.5 ±2.4	11.6 ±4.4	7.1 ±3.7	8.0 ±4.0	$[M+K]^+$	PE(18:2)	$C_{23}H_{44}NO_7P$
		518.26436	518.26431	518.26429	518.26430	518.26435		5.3 ±2.3	8.4 ±4.2	6.7 ±3.6	8.1 ±4.0	$[M+K]^+$	PE(18:1)	$C_{23}H_{46}NO_7P$
		504.28506	504.28506	504.28511	504.28509	504.28508		8.5	25.2	15.6	23.3	$[M+K]^+$	PE(P-18:0)	$C_{23}H_{48}NO_6P$

Classification	Electric field (Measured *m/z*)					Calculated *m/z*	Electric field (Average *S/N*, n=3)					Assignment		
	**Matrix coating**	**I**	**II**	**III**	**IV**		**Matrix coating**	**I**	**II**	**III**	**IV**	Ion form	Compound	Molecular formula
	**Spray (3-s)**	Off	On	On	Off		**Spray (3-s)**	Off	On	On	Off			
	**Incubation (60-s)**	Off	On	Off	On		**Incubation (60-s)**	Off	On	Off	On			
	**Drying (90-s)**	Off	On	Off	On		**Drying (90-s)**	Off	On	Off	On			
								±4.3	±5.7	±4.6	±5.3			
		520.28004	520.28006	520.28012	520.28003	520.28000		5.3 ±2.2	15.4 ±4.5	9.6 ±4.2	13.6 ±4.4	$[M+K]^+$	PE(18:0)	$C_{23}H_{48}NO_7P$
		-	540.24881	-	540.24875	540.24870		-	23.6 ±5.3	-	17.2 ±4.8	$[M+K]^+$	PE(20:4)	$C_{25}H_{44}NO_7P$
		-	542.26438	542.26435	542.26443	542.26435		-	33.4 ±7.2	20.8 ±5.2	28.8 ±5.5	$[M+K]^+$	PE(20:3)	$C_{25}H_{46}NO_7P$
		544.27993	544.28009	544.28006	544.28016	544.28000		5.0 ±2.1	8.5 ±4.3	7.2 ±3.8	7.8 ±4.0	$[M+K]^+$	PE(20:2)	$C_{25}H_{48}NO_7P$
		546.29558	546.29566	546.29555	546.29560	546.29565		6.5 ±3.4	26.2 ±5.4	15.4 ±4.5	19.7 ±5.2	$[M+K]^+$	PE(20:1)	$C_{25}H_{50}NO_7P$
		510.35532	510.35552	510.35540	510.35541	510.35542		5.0 ±2.2	10.5 ±4.3	7.4 ±3.9	8.1 ±4.1	$[M+H]^+$	PE(20:0)	$C_{25}H_{52}NO_7P$
		-	548.31133	548.31144	548.31123	548.31130		-	9.5 ±4.1	5.1 ±2.3	6.8 ±3.3	$[M+K]^+$		
		564.24865	564.24874	564.24876	564.24868	564.24870		6.8 ±3.4	11.4 ±4.3	9.3 ±4.1	10.0 ±4.3	$[M+K]^+$	PE(22:6)	$C_{27}H_{44}NO_7P$
		568.28001	568.27999	568.28016	568.28013	568.28000		5.6 ±2.6	14.4 ±4.2	8.8 ±4.0	11.2 ±4.3	$[M+K]^+$	PE(22:4)	$C_{27}H_{48}NO_7P$
		572.31136	572.31125	572.31130	572.31131	572.31130		7.3 ±3.9	17.5 ±5.1	10.4 ±4.4	15.3 ±4.6	$[M+K]^+$	PE(22:2)	$C_{27}H_{52}NO_7P$
		574.32694	574.32685	574.32687	574.32697	574.32695		6.3 ±3.4	21.9 ±5.3	15.1 ±4.5	19.3 ±4.8	$[M+K]^+$	PE(22:1)	$C_{27}H_{54}NO_7P$
		-	538.38682	-	-	538.38672		-	9.9 ±4.4	-	-	$[M+H]^+$	PE(22:0)	$C_{27}H_{56}NO_7P$
		-	560.36859	560.36855	560.36857	560.36866		-	24.7 ±5.5	11.9 ±4.5	18.8 ±5.0	$[M+Na]^+$		
		602.35817	602.35823	602.35824	602.38532	602.35825		6.7 ±3.3	31.2 ±5.9	14.0 ±4.3	25.8 ±5.6	$[M+K]^+$	LysoPE(24:1)	$C_{29}H_{58}NO_7P$
		-	644.36882	644.36875	644.36883	644.36881		-	9.6 ±4.3	6.6 ±3.5	9.2 ±4.2	$[M+K]^+$	PE(26:1)	$C_{31}H_{60}NO_8P$
		646.38451	646.38438	646.38443	646.38450	646.38446		8.2 ±4.4	26.4 ±5.4	15.1 ±5.0	23.6 ±6.3	$[M+K]^+$	PE(26:0)	$C_{31}H_{62}NO_8P$
		756.49407	756.49399	756.49403	756.49403	756.49401		5.4 ±2.3	10.2 ±4.6	7.5 ±3.5	8.9 ±4.3	$[M+K]^+$	PE(34:1)	$C_{39}H_{76}NO_8P$
		740.49934	740.49921	740.49931	740.49898	740.49910		76.0	233.7	186.6	222.8	$[M+K]^+$	PE(P-34:1)	$C_{39}H_{76}NO_7P$

Classification	Electric field (Measured *m/z*)					Calculated *m/z*	Electric field (Average *S/N*, n=3)					Assignment		
	**Matrix coating**	**I**	**II**	**III**	**IV**		**Matrix coating**	**I**	**II**	**III**	**IV**	Ion form	Compound	Molecular formula
	**Spray (3-s)**	Off	On	On	Off		**Spray (3-s)**	Off	On	On	Off			
	**Incubation (60-s)**	Off	On	Off	On		**Incubation (60-s)**	Off	On	Off	On			
	**Drying (90-s)**	Off	On	Off	On		**Drying (90-s)**	Off	On	Off	On			
								±10.1	±16.5	±13.9	±15.7			
		-	742.51474	742.51473	742.51482	742.51475		11.0 ±4.6	24.3 ±6.5	16.2 ±5.3	20.5 ±5.7	$[M+K]^+$	PE(P-34:0)	$C_{39}H_{78}NO_7P$
		750.44725	750.44705	750.44706	750.44698	750.44706		8.9 ±5.0	36.2 ±6.9	25.3 ±5.6	33.0 ±6.4	$[M+K]^+$	PE(34:4)	$C_{39}H_{70}NO_8P$
		758.50955	758.50960	758.50973	758.50987	758.50966		6.1 ±3.1	17.1 ±5.3	9.1 ±4.0	16.2 ±5.1	$[M+K]^+$	PE(34:0)	$C_{39}H_{78}NO_8P$
		764.49915	764.49904	764.49920	764.49909	764.49910		12.4 ±4.8	21.6 ±5.6	16.3 ±5.1	19.0 ±5.3	$[M+K]^+$	PE(P-36:3)	$C_{41}H_{76}NO_7P$
		780.49414	780.49402	780.49408	780.49414	780.49401		6.9 ±3.4	13.8 ±5.7	8.5 ±4.2	11.7 ±5.3	$[M+K]^+$	PE(36:3)	$C_{41}H_{76}NO_8P$
		-	782.50962	-	782.50953	782.50966		-	9.6 ±4.5	-	9.3 ±4.3	$[M+K]^+$	PE(36:2)	$C_{41}H_{78}NO_8P$
		768.53035	768.53033	768.53054	768.53057	768.53040		5.4 ±2.5	15.3 ±5.0	8.7 ±4.3	13.7 ±4.8	$[M+K]^+$	PE(P-36:1)	$C_{41}H_{80}NO_7P$
		784.52527	784.52540	784.52529	784.52544	784.52531		6.3 ±2.8	14.1 ±4.6	9.7 ±4.5	12.8 ±5.1	$[M+K]^+$	PE(36:1)	$C_{41}H_{80}NO_8P$
		770.54613	770.54608	770.54604	770.54610	770.54605		9.6 ±4.4	62.3 ±8.4	39.9 ±7.1	47.8 ±7.6	$[M+K]^+$	PE(P-36:0)	$C_{41}H_{82}NO_7P$
		748.58529	748.58509	748.58494	748.58518	748.58508		14.1 ±4.6	31.0 ±6.5	20.7 ±5.2	23.0 ±5.5	$[M+H]^+$	PE(36:0)	$C_{41}H_{82}NO_8P$
		786.48345	786.48353	786.48341	786.48361	786.48345		10.5 ±4.5	24.9 ±5.4	14.7 ±4.8	22.4 ±5.3	$[M+K]^+$	PE(P-38:6)	$C_{43}H_{74}NO_7P$
		802.47833	802.47840	802.47836	802.47839	802.47836		5.0 ±2.3	8.7 ±4.3	6.1 ±3.1	7.6 ±4.1	$[M+K]^+$	PE(38:6)	$C_{43}H_{74}NO_8P$
		788.49920	788.49915	788.49908	788.49917	788.49910		9.7 ±4.6	21.6 ±5.2	15.4 ±5.0	19.6 ±5.3	$[M+K]^+$	PE(P-38:5)	$C_{43}H_{76}NO_7P$
		804.49397	804.49410	804.49407	804.49406	804.49401		8.4 ±4.2	14.6 ±4.7	10.2 ±4.2	13.7 ±4.8	$[M+K]^+$	PE(38:5)	$C_{43}H_{76}NO_8P$
		790.51471	790.51477	790.51460	790.51479	790.51475		10.2 ±4.3	23.5 ±5.6	16.3 ±5.1	21.4 ±5.3	$[M+K]^+$	PE(P-38:4)	$C_{43}H_{78}NO_7P$
		806.50958	806.50971	806.50965	806.50973	806.50966		8.6 ±4.3	48.3 ±7.7	34.6 ±6.7	43.9 ±7.2	$[M+K]^+$	PE(38:4)	$C_{43}H_{78}NO_8P$
		-	792.53052	-	-	792.53040		-	6.3 ±3.2	-	-	$[M+K]^+$	PE(P-38:3)	$C_{43}H_{80}NO_7P$
		-	810.54093	-	-	810.54096		-	5.1	-	-	$[M+K]^+$	PE(38:2)	$C_{43}H_{82}NO_8P$

Classification	Electric field (Measured *m/z*)					Calculated *m/z*	Electric field (Average *S/N*, n=3)					Assignment		
	**Matrix coating**	I	II	III	IV		**Matrix coating**	I	II	III	IV	Ion form	Compound	Molecular formula
	**Spray (3-s)**	Off	On	On	Off		**Spray (3-s)**	Off	On	On	Off			
	**Incubation (60-s)**	Off	On	Off	On		**Incubation (60-s)**	Off	On	Off	On			
	**Drying (90-s)**	Off	On	Off	On		**Drying (90-s)**	Off	On	Off	On			
									±2.6					
		774.60072	774.60067	774.60074	774.60072	774.60073		23.1 ±5.5	60.0 ±8.3	45.3 ±7.3	50.5 ±7.8	[M+H]$^+$	PE(38:1)	$C_{43}H_{84}NO_8P$
		812.55651	812.55668	812.55673	812.55661	812.55661		6.9 ±3.5	17.3 ±5.3	12.7 ±4.6	15.9 ±5.0	[M+K]$^+$		
		812.49903	812.49919	812.49909	812.49917	812.49910		15.9 ±4.8	33.2 ±6.7	28.6 ±5.6	30.1 ±6.5	[M+K]$^+$	PE(P-40:7)	$C_{45}H_{76}NO_7P$
		-	828.49405	828.49409	828.49415	828.49401		-	23.2 ±5.6	16.7 ±5.2	20.6 ±5.4	[M+K]$^+$	PE(40:7)	$C_{45}H_{76}NO_8P$
		814.51463	814.51471	814.51467	814.51485	814.51475		7.4 ±3.5	14.2 ±5.0	11.2 ±4.3	13.7 ±4.7	[M+K]$^+$	PE(P-40:6)	$C_{45}H_{78}NO_7P$
		830.50961	830.50970	830.50973	830.50958	830.50966		5.4 ±2.3	15.3 ±4.8	8.4 ±4.2	13.1 ±4.6	[M+K]$^+$	PE(40:6)	$C_{45}H_{78}NO_8P$
		816.53043	816.53049	816.53079	816.53036	816.53040		6.8 ±3.5	17.1 ±5.2	9.2 ±4.5	16.2 ±5.1	[M+K]$^+$	PE(P-40:5)	$C_{45}H_{80}NO_7P$
		-	832.52534	-	-	832.52531		-	5.6 ±2.4	-	-	[M+K]$^+$	PE(40:5)	$C_{45}H_{80}NO_8P$
		818.54613	818.54607	818.54617	818.54601	818.54605		6.7 ±3.5	17.8 ±5.3	10.8 ±4.1	15.3 ±4.7	[M+K]$^+$	PE(P-40:4)	$C_{45}H_{82}NO_7P$
		834.54078	834.54085	834.54090	834.54089	834.54096		7.8 ±3.6	21.6 ±5.5	16.8 ±4.9	20.5 ±5.3	[M+K]$^+$	PE(40:4)	$C_{45}H_{82}NO_8P$
		802.63207	802.63198	802.63211	802.63209	802.63203		11.7 ±4.5	23.8 ±5.6	16.9 ±4.8	22.4 ±5.4	[M+H]$^+$	PE(40:1)	$C_{45}H_{88}NO_8P$
		-	850.47840	-	850.47827	850.47836		-	7.5 ±3.5	-	6.4 ±3.0	[M+K]$^+$	PE(42:10)	$C_{47}H_{74}NO_8P$
		852.49400	852.49412	852.49405	852.49408	852.49401		18.5 ±5.0	96.6 ±14.1	62.7 ±8.5	78.1 ±12.4	[M+K]$^+$	PE(42:9)	$C_{47}H_{76}NO_8P$
		-	854.50973	854.50951	854.50933	854.50966		-	36.4 ±6.8	26.5 ±5.7	30.8 ±6.1	[M+K]$^+$	PE(42:8)	$C_{47}H_{78}NO_8P$
		-	856.52525	-	856.52542	856.52531		-	9.1 ±4.5	-	6.7 ±3.6	[M+K]$^+$	PE(42:7)	$C_{47}H_{80}NO_8P$
		-	858.54090	-	-	858.54096		-	5.3 ±2.2	-	-	[M+K]$^+$	PE(42:6)	$C_{47}H_{82}NO_8P$
		-	824.61629	824.61635	824.61643	824.61638		-	10.5 ±4.2	7.4 ±3.5	8.8 ±3.8	[M+H]$^+$	PE(42:4)	$C_{47}H_{86}NO_8P$
		810.63716	810.63713	810.63724	817.63726	810.63712		6.8	20.3	13.5	18.7	[M+H]$^+$	PE(O-42:4)	$C_{47}H_{88}NO_7P$

Classification	Electric field (Measured *m/z*)					Calculated *m/z*	Electric field (Average *S/N*, n=3)					Assignment		
	Matrix coating	I	II	III	IV		Matrix coating	I	II	III	IV	Ion form	Compound	Molecular formula
	Spray (3-s)	Off	On	On	Off		Spray (3-s)	Off	On	On	Off			
	Incubation (60-s)	Off	On	Off	On		Incubation (60-s)	Off	On	Off	On			
	Drying (90-s)	Off	On	Off	On		Drying (90-s)	Off	On	Off	On			
								±3.6	±5.2	±4.7	±5.0			
		864.58803	864.58785	864.58794	864.58787	864.58791		8.8 ±3.6	18.5 ±5.1	11.3 ±4.4	16.5 ±4.8	$[M+K]^+$	PE(42:3)	$C_{47}H_{88}NO_8P$
		850.60850	850.60867	850.60862	850.60868	850.60865		5.2 ±2.1	12.7 ±4.5	7.5 ±3.5	11.8 ±4.6	$[M+K]^+$	PE(P-42:2)	$C_{47}H_{90}NO_7P$
		845.67432	845.67416	845.67420	845.67429	845.67423		5.0 ±2.1	11.2 ±4.3	7.7 ±3.6	10.8 ±4.1	$[M+Na]^+$	PE(42:2)	$C_{47}H_{90}NO_8P$
		-	852.62425	-	852.62424	852.62430		-	14.1 ±5.0	-	11.9 ±4.6	$[M+K]^+$	PE(P-42:1)	$C_{47}H_{92}NO_7P$
		868.61925	868.61924	868.61931	868.61936	868.61921		6.2 ±3.1	13.4 ±4.8	8.5 ±3.5	11.7 ±4.5	$[M+K]^+$	PE(42:1)	$C_{47}H_{92}NO_8P$
		870.63493	870.63481	870.63470	870.63484	870.63486		5.9 ±2.8	13.3 ±4.7	9.8 ±4.0	12.1 ±4.7	$[M+K]^+$	PE(42:0)	$C_{47}H_{94}NO_8P$
		-	878.50961	878.50958	878.50963	878.50966		-	8.9 ±3.7	6.3 ±3.3	7.5 ±3.6	$[M+K]^+$	PE(44:10)	$C_{49}H_{78}NO_8P$
		-	880.52527	-	-	880.52531		-	5.3 ±2.2	-	-	$[M+K]^+$	PE(44:9)	$C_{49}H_{80}NO_8P$
		886.57231	886.57228	886.57226	886.57230	886.57226		5.0 ±2.0	17.8 ±5.3	12.3 ±4.3	15.1 ±4.9	$[M+K]^+$	PE(44:6)	$C_{49}H_{86}NO_8P$
		888.58787	888.58790	888.58786	888.58796	888.58791		5.0 ±2.0	15.0 ±4.8	10.4 ±4.1	14.5 ±5.2	$[M+K]^+$	PE(44:5)	$C_{49}H_{88}NO_8P$
		-	896.65061	-	896.65058	896.65051		-	7.4 ±3.4	-	5.1 ±2.2	$[M+K]^+$	PE(44:1)	$C_{49}H_{96}NO_8P$
Phosphatidic acids (PAs)														
		475.22224	475.22212	475.22218	475.22207	475.22215		5.0 ±2.1	12.3 ±4.3	7.4 ±3.3	11.5 ±4.4	$[M+K]^+$	PA(18:1)	$C_{21}H_{41}O_7P$
		477.23781	477.23774	477.23784	477.23772	477.23780		5.1 ±2.2	7.6 ±3.4	6.4 ±3.1	7.1 ±3.3	$[M+K]^+$	PA(18:0)	$C_{21}H_{43}O_7P$
		497.20661	497.20653	497.20651	497.20642	497.20650		15.7 ±5.1	30.7 ±6.3	22.4 ±5.6	24.6 ±5.8	$[M+K]^+$	PA(20:4)	$C_{23}H_{39}O_7P$
		499.22217	499.22225	499.22209	499.22218	499.22215		5.0 ±2.2	21.2 ±5.3	10.2 ±4.2	16.1 ±5.0	$[M+K]^+$	PA(20:3)	$C_{23}H_{41}O_7P$
		501.23790	501.23785	501.23780	501.23774	501.23780		5.1 ±2.3	18.2 ±6.0	13.0 ±4.9	15.0 ±5.2	$[M+K]^+$	PA(20:2)	$C_{23}H_{43}O_7P$

Classification		Electric field (Measured *m/z*)				Calculated *m/z*		Electric field (Average *S/N*, n=3)				Assignment		
	**Matrix coating**	**I**	**II**	**III**	**IV**		**Matrix coating**	**I**	**II**	**III**	**IV**	Ion form	Compound	Molecular formula
	**Spray (3-s)**	Off	On	On	Off		**Spray (3-s)**	Off	On	On	Off			
	**Incubation (60-s)**	Off	On	Off	On		**Incubation (60-s)**	Off	On	Off	On			
	**Drying (90-s)**	Off	On	Off	On		**Drying (90-s)**	Off	On	Off	On			
		487.27953	487.27954	487.27947	487.27953	487.27951		11.4 ±4.5	20.8 ±5.2	14.7 ±4.7	16.0 ±5.0	$[M+Na]^+$	PA(20:1)	$C_{23}H_{45}O_7P$
		503.25347	503.25347	503.25344	503.25342	503.25345		5.2 ±2.1	17.7 ±5.7	12.3 ±4.3	14.6 ±4.7	$[M+K]^+$		
		525.23791	525.23777	525.23780	525.23776	525.23780		7.8 ±3.5	22.4 ±5.6	12.6 ±4.4	20.3 ±5.1	$[M+K]^+$	PA(22:4)	$C_{25}H_{43}O_7P$
		531.28481	531.28483	531.28467	531.28473	531.28475		8.0 ±3.4	15.2 ±4.9	11.4 ±4.7	14.6 ±4.8	$[M+K]^+$	PA(22:1)	$C_{25}H_{49}O_7P$
		533.30051	533.30047	533.30043	533.30044	533.30040		5.2 ±2.3	19.7 ±4.3	13.2 ±3.3	17.9 ±4.2	$[M+K]^+$	PA(22:0)	$C_{25}H_{51}O_7P$
		679.37362	679.37357	679.37359	679.37354	679.37356		5.3 ±3.4	20.4 ±4.2	16.3 ±3.5	18.7 ±4.1	$[M+K]^+$	PA(32:4)	$C_{35}H_{61}O_8P$
		681.38945	681.38942	681.38920	681.38926	681.38921		5.0 ±2.5	16.6 ±3.4	10.7 ±3.2	15.1 ±3.3	$[M+K]^+$	PA(32:3)	$C_{35}H_{63}O_8P$
		683.40504	683.40493	683.40481	683.40477	683.40486		5.2 ±2.4	9.5 ±3.1	7.8 ±2.9	9.0 ±3.0	$[M+K]^+$	PA(32:2)	$C_{35}H_{65}O_8P$
		685.42062	685.42053	685.42053	685.42041	685.42051		5.2 ±2.4	16.4 ±4.2	10.4 ±3.5	14.7 ±4.3	$[M+K]^+$	PA(32:1)	$C_{35}H_{67}O_8P$
		687.43617	687.43623	687.43608	687.43620	687.43616		5.3 ±2.5	14.1 ±3.3	9.9 ±3.1	13.5 ±3.2	$[M+K]^+$	PA(32:0)	$C_{35}H_{69}O_8P$
		643.50361	643.50371	643.50363	643.50364	643.50370		5.2 ±2.4	13.9 ±3.6	8.4 ±2.6	12.8 ±3.2	$[M+Na]^+$	PA(O-32:0)	$C_{35}H_{73}O_6P$
		-	709.42057	709.42040	709.42052	709.42051		5.3 ±2.3	23.4 ±5.2	18.8 ±4.6	22.1 ±5.0	$[M+K]^+$	PA(34:3)	$C_{37}H_{67}O_8P$
		711.43619	711.43626	711.43614	711.43624	711.43616		6.1 ±2.6	28.0 ±5.3	20.5 ±5.1	26.2 ±5.3	$[M+K]^+$	PA(34:2)	$C_{37}H_{69}O_8P$
		697.4780	697.47799	697.47786	697.47787	697.47788		16.8 ±3.6	56.3 ±7.8	41.7 ±7.3	47.4 ±7.5	$[M+Na]^+$	PA(34:1)	$C_{37}H_{71}O_8P$
		713.45177	713.45186	713.45179	713.45180	713.45181		93.9 ±8.9	463.8 ±18.7	301.2 ±14.4	384.3 ±16.2	$[M+K]^+$		
		-	699.47255	-	699.47246	699.47255		-	6.5 ±3.1	-	5.1 ±2.3	$[M+K]^+$	PA(O-34:1)	$C_{37}H_{73}O_7P$
		701.45161	701.45172	701.45175	701.45154	701.45166		5.3 ±2.5	16.5 ±4.7	12.4 ±3.6	14.9 ±4.5	$[M+Na]^+$	PA(P-36:5)	$C_{39}H_{67}O_7P$
		733.42053	733.42058	733.42052	733.42047	733.42051		5.2 ±2.1	14.8 ±4.2	8.5 ±3.6	13.2 ±4.0	$[M+K]^+$	PA(36:5)	$C_{39}H_{67}O_8P$

Classification	Electric field (Measured *m/z*)					Calculated *m/z*	Electric field (<u>Average *S/N*, n=3)</u>					Assignment		
	**Matrix coating**	**I**	**II**	**III**	**IV**		**Matrix coating**	**I**	**II**	**III**	**IV**	Ion form	Compound	Molecular formula
	**Spray (3-s)**	Off	On	On	Off		**Spray (3-s)**	Off	On	On	Off			
	**Incubation (60-s)**	Off	On	Off	On		**Incubation (60-s)**	Off	On	Off	On			
	**Drying (90-s)**	Off	On	Off	On		**Drying (90-s)**	Off	On	Off	On			
		-	735.43625	735.43623	735.43618	735.43616		-	8.0 ±3.6	5.3 ±2.4	7.6 ±2.9	$[M+K]^+$	PA(36:4)	$C_{39}H_{69}O_8P$
		737.45191	737.45183	737.45172	737.45179	737.45181		6.1 ±2.8	23.2 ±4.7	18.4 ±4.3	22.0 ±4.5	$[M+K]^+$	PA(36:3)	$C_{39}H_{71}O_8P$
		723.49342	723.49358	723.49363	723.49358	723.49353		14.8 ±4.4	65.0 ±7.8	57.1 ±6.3	62.7 ±7.0	$[M+Na]^+$	PA(36:2)	$C_{39}H_{73}O_8P$
		739.46750	739.46738	739.46742	739.46743	739.46746		108.8 ±9.5	542.8 ±20.1	443.8 ±17.3	522.8 ±19.8	$[M+K]^+$		
		-	741.48304	741.48306	741.48315	741.48311		-	9.3 ±4.1	7.4 ±3.3	8.5 ±3.4	$[M+K]^+$	PA(36:1)	$C_{39}H_{75}O_8P$
		727.46731	727.46727	727.46726	727.46725	727.46731		7.8 ±3.5	11.4 ±4.3	10.1 ±4.1	11.0 ±4.2	$[M+Na]^+$	PA(P-38:6)	$C_{41}H_{69}O_7P$
		-	759.43613	-	759.43609	759.43616		-	7.1 ±3.2	-	6.5 ±2.8	$[M+K]^+$	PA(38:6)	$C_{41}H_{69}O_8P$
		761.45177	761.45178	761.45174	761.45189	761.45181		24.2 ±6.1	91.0 ±8.7	84.2 ±8.1	86.2 ±8.3	$[M+K]^+$	PA(38:5)	$C_{41}H_{71}O_8P$
		725.51149	725.51157	725.51167	725.51147	725.51158		6.7 ±3.6	25.4 ±5.2	23.9 ±5.0	24.5 ±5.1	$[M+H]^+$	PA(38:4)	$C_{41}H_{73}O_8P$
		763.46737	763.46801	763.46702	763.46733	763.46746		9.4 ±3.8	38.5 ±5.8	32.0 ±5.6	33.8 ±5.7	$[M+K]^+$		
		-	749.50914	-	-	749.50918		-	6.1 ±2.5	-	-	$[M+Na]^+$	PA(38:3)	$C_{41}H_{75}O_8P$
		765.48307	765.48304	765.48318	765.48316	765.48311		10.6 ±4.5	29.1 ±8.2	26.5 ±7.3	27.4 ±7.5	$[M+K]^+$		
		751.52478	751.52480	751.52479	751.52476	751.52483		5.5 ±2.4	15.2 ±4.1	13.0 ±4.0	14.7 ±4.2	$[M+Na]^+$	PA(38:2)	$C_{41}H_{77}O_8P$
		767.49873	767.49979	767.49883	767.49887	767.49876		33.4 ±5.7	138.7 ±10.2	122.8 ±9.7	127.8 ±10.0	$[M+K]^+$		
		771.53006	771.53014	771.53009	771.53006	771.53006		5.1 ±2.4	10.3 ±5.3	8.4 ±4.7	9.1 ±5.0	$[M+K]^+$	PA(38:0)	$C_{41}H_{81}O_8P$
		785.45186	785.45177	785.45189	785.45182	785.45181		14.0 ±4.1	33.9 ±5.8	26.6 ±4.9	33.5 ±5.8	$[M+K]^+$	PA(40:7)	$C_{43}H_{71}O_8P$
		-	787.46748	787.46738	787.46753	787.46746		-	22.4 ±5.2	17.5 ±4.6	20.7 ±5.0	$[M+K]^+$	PA(40:6)	$C_{43}H_{73}O_8P$
		-	773.50925	-	773.50926	773.50918		-	8.6 ±3.8	-	7.5 ±3.4	$[M+Na]^+$	PA(40:5)	$C_{43}H_{75}O_8P$

Classification	Electric field (Measured *m/z*)					Calculated *m/z*	Electric field (Average *S/N*, n=3)					Assignment		
	**Matrix coating**	**I**	**II**	**III**	**IV**		**Matrix coating**	**I**	**II**	**III**	**IV**	Ion form	Compound	Molecular formula
	**Spray (3-s)**	Off	On	On	Off		**Spray (3-s)**	Off	On	On	Off			
	**Incubation (60-s)**	Off	On	Off	On		**Incubation (60-s)**	Off	On	Off	On			
	**Drying (90-s)**	Off	On	Off	On		**Drying (90-s)**	Off	On	Off	On			
		789.48308	789.48308	789.48318	789.48305	789.48311		12.4 ±3.6	56.6 ±7.9	46.7 ±7.1	51.3 ±7.3	$[M+K]^+$		
		777.54052	777.54051	777.54047	777.54046	777.54048		5.7 ±3.0	16.6 ±5.3	12.4 ±4.7	14.9 ±5.1	$[M+Na]^+$	PA(40:3)	$C_{43}H_{79}O_8P$
		-	809.45183	-	809.45177	809.45181		-	7.9 ±4.5	-	6.4 ±4.1	$[M+K]^+$	PA(42:9)	$C_{45}H_{71}O_8P$
Phosphoglycerols (PGs)														
		547.24327	547.24324	547.24333	547.24336	547.24328		5.1 ±2.0	11.7 ±5.0	8.2 ±4.5	8.9 ±4.7	$[M+K]^+$	PG(18:2)	$C_{24}H_{45}O_9P$
		573.25901	573.25897	573.25893	573.25897	573.25893		5.0 ±2.0	9.9 ±4.6	6.5 ±3.3	8.7 ±4.1	$[M+K]^+$	PG(20:3)	$C_{26}H_{47}O_9P$
		559.30086	559.30057	559.30067	559.30066	559.30064		14.0 ±5.3	71.9 ±9.9	36.6 ±7.2	53.3 ±8.6	$[M+Na]^+$	PG(20:2)	$C_{26}H_{49}O_9P$
		599.27458	599.27461	599.27451	599.27448	599.27458		7.1 ±3.4	14.2 ±5.6	8.8 ±3.9	13.5 ±5.3	$[M+K]^+$	PG(22:4)	$C_{28}H_{49}O_9P$
		603.30597	603.30578	603.30595	603.30596	603.30588		14.0 ±5.7	31.2 ±7.0	23.7 ±5.7	27.8 ±6.8	$[M+K]^+$	PG(22:2)	$C_{28}H_{53}O_9P$
		-	745.47807	745.47808	745.47811	745.47803		-	11.2 ±4.8	8.7 ±3.7	10.3 ±4.8	$[M+K]^+$	PG(P-32:0)	$C_{38}H_{75}O_9P$
		-	743.48580	-	743.48569	743.48576		-	7.6 ±3.5	-	5.1 ±2.1	$[M+H]^+$	PG(34:4)	$C_{40}H_{71}O_{10}P$
		783.45733	783.45732	783.45728	783.45734	783.45729		13.1 ±5.0	36.9 ±7.7	31.4 ±7.2	34.7 ±7.5	$[M+K]^+$	PG(34:3)	$C_{40}H_{73}O_{10}P$
		793.49907	793.49904	793.49909	793.49911	793.49901		12.1 ±4.8	29.8 ±7.2	19.5 ±6.6	28.0 ±7.1	$[M+Na]^+$	PG(36:4)	$C_{42}H_{75}O_{10}P$
		817.53557	817.53554	817.53548	817.53560	817.53554		6.8 ±3.2	19.5 ±6.5	14.3 ±6.1	17.5 ±6.3	$[M+K]^+$	PG(36:0)	$C_{42}H_{83}O_{10}P$
		-	801.56398	801.56403	801.56405	801.56401		-	31.0 ±7.5	21.5 ±5.8	25.8 ±6.4	$[M+H]^+$	PG(38:3)	$C_{44}H_{81}O_{10}P$
		825.56158	825.56156	825.56150	825.65160	825.56161		10.8 ±4.3	40.0 ±11.2	31.9 ±7.7	39.5 ±10.4	$[M+Na]^+$	PG(38:2)	$C_{44}H_{83}O_{10}P$
		-	887.51987	-	-	887.51989		-	5.1 ±2.6	-	-	$[M+K]^+$	PG(42:7)	$C_{48}H_{81}O_{10}P$

Classification	Electric field (Measured m/z)					Calculated m/z	Electric field (Average S/N, n=3)					Assignment		
	**Matrix coating**	**I**	**II**	**III**	**IV**		**Matrix coating**	**I**	**II**	**III**	**IV**	Ion form	Compound	Molecular formula
	**Spray (3-s)**	Off	On	On	Off		**Spray (3-s)**	Off	On	On	Off			
	**Incubation (60-s)**	Off	On	Off	On		**Incubation (60-s)**	Off	On	Off	On			
	**Drying (90-s)**	Off	On	Off	On		**Drying (90-s)**	Off	On	Off	On			
Phosphatidylserine (PS)														
		576.30620	576.30612	576.30624	576.30613	576.30621		6.3 ±3.1	17.7 ±5.1	12.4 ±4.4	15.8 ±5.4	$[M+K]^+$	PS(P-20:0)	$C_{26}H_{52}NO_8P$
		592.30116	592.30114	592.30114	592.30117	592.30113		5.3 ±2.6	15.3 ±5.4	10.7 ±5.1	13.6 ±4.2	$[M+K]^+$	PS(20:0)	$C_{26}H_{52}NO_9P$
		612.26989	612.26986	612.26980	612.26991	612.26983		7.9 ±3.6	33.5 ±9.6	27.3 ±6.4	32.3 ±7.8	$[M+K]^+$	PS(22:4)	$C_{28}H_{48}NO_9P$
		-	780.47862	780.47855	780.47858	780.47861		-	14.8 ±4.6	10.3 ±5.0	13.8 ±4.8	$[M+Na]^+$	PS(34:3)	$C_{40}H_{72}NO_{10}P$
		-	808.50986	808.50993	808.50986	808.50991		-	12.0 ±4.8	5.3 ±2.3	10.5 ±4.6	$[M+Na]^+$	PS(36:3)	$C_{42}H_{76}NO_{10}P$
		828.51508	828.51517	828.51511	828.51509	828.51514		38.0 ±7.9	107.8 ±9.4	89.6 ±8.6	92.6 ±8.7	$[M+K]^+$	PS(36:1)	$C_{42}H_{80}NO_{10}P$
		-	824.44713	-	824.44735	824.44731		-	10.5 ±5.0	-	9.0 ±4.1	$[M+Na]^+$	PS(38:9)	$C_{44}H_{68}NO_{10}P$
		-	826.46296	826.46293	826.46302	826.46296		-	19.8 ±6.1	14.0 ±5.3	17.3 ±5.7	$[M+Na]^+$	PS(38:8)	$C_{44}H_{70}NO_{10}P$
		846.46837	846.46807	846.46819	846.46814	846.46819		35.7 ±7.5	165.6 ±15.0	134.5 ±13.4	145.4 ±14.7	$[M+K]^+$	PS(38:6)	$C_{44}H_{74}NO_{10}P$
		830.47331	830.47324	830.47338	830.47331	830.47328		9.6 ±4.1	49.2 ±11.9	38.6 ±10.2	47.9 ±11.6	$[M+K]^+$	PS(P-38:6)	$C_{44}H_{74}NO_9P$
		-	834.52553	-	834.52561	834.52556		-	8.9 ±4.6	-	6.7 ±3.4	$[M+Na]^+$	PS(38:4)	$C_{44}H_{78}NO_{10}P$
		-	854.49423	-	854.49424	854.49426		-	7.3 ±3.3	-	5.1 ±2.4	$[M+Na]^+$	PS(40:8)	$C_{46}H_{74}NO_{10}P$
		-	856.50986	-	-	856.50991		-	5.3 ±2.5	-	-	$[M+Na]^+$	PS(40:7)	$C_{46}H_{76}NO_{10}P$
		-	858.52557	858.52565	858.52547	858.52556		-	18.0 ±5.9	12.5 ±4.9	16.9 ±5.6	$[M+Na]^+$	PS(40:6)	$C_{46}H_{78}NO_{10}P$
		-	860.54119	-	860.54120	860.54121		-	8.9 ±4.5	-	6.4 ±3.4	$[M+Na]^+$	PS(40:5)	$C_{46}H_{80}NO_{10}P$
		846.62196	846.62180	846.62192	846.62183	846.62186		34.2 ±7.1	54.7 ±12.4	41.5 ±10.5	45.5 ±11.6	$[M+H]^+$	PS(40:1)	$C_{46}H_{88}NO_{10}P$
		-	830.62688	-	-	830.62695		-	6.2 ±3.1	-	-	$[M+H]^+$	PS(P-40:1)	$C_{46}H_{88}NO_9P$

Classification	Electric field (Measured *m/z*)					Calculated *m/z*	Electric field (Average *S/N*, n=3)					Assignment		
	**Matrix coating**	**I**	**II**	**III**	**IV**		**Matrix coating**	**I**	**II**	**III**	**IV**	Ion form	Compound	Molecular formula
	**Spray (3-s)**	Off	On	On	Off		**Spray (3-s)**	Off	On	On	Off			
	**Incubation (60-s)**	Off	On	Off	On		**Incubation (60-s)**	Off	On	Off	On			
	**Drying (90-s)**	Off	On	Off	On		**Drying (90-s)**	Off	On	Off	On			
		848.63754	848.63750	848.63742	848.63744	848.63751		9.6 ±4.3	49.4 ±15.3	39.8 ±14.7	47.2 ±15.0	$[M+H]^+$	PS(40:0)	$C_{46}H_{90}NO_{10}P$
		-	884.54118	884.54124	884.54120	884.54121		-	9.6 ±4.6	6.4 ±3.1	7.8 ±3.7	$[M+Na]^+$	PS(42:7)	$C_{48}H_{80}NO_{10}P$
Phosphatidylinositols (PIs)														
		-	919.47341	-	919.47332	919.47334		-	8.9 ±3.9	-	6.5 ±3.1	$[M+K]^+$	PI(38:7)	$C_{47}H_{77}O_{13}P$
		925.52030	925.52032	925.52027	925.52028	925.52029		6.3 ±3.0	26.7 ±6.1	14.5 ±5.3	19.3 ±6.1	$[M+K]^+$	PI(38:4)	$C_{47}H_{83}O_{13}P$
		945.48898	945.48891	945.48894	945.48906	945.48899		5.1 ±2.0	24.6 ±5.7	11.7 ±4.6	15.3 ±5.0	$[M+K]^+$	PI(40:8)	$C_{49}H_{79}O_{13}P$
		915.59573	915.59576	915.59568	915.59565	915.59571		6.7 ±3.2	24.8 ±5.8	13.7 ±4.7	20.4 ±5.2	$[M+H]^+$	PI(40:4)	$C_{49}H_{87}O_{13}P$
		-	931.53314	931.53319	931.53315	931.53311		-	16.8 ±5.1	9.1 ±4.1	11.5 ±4.7	$[M+H]^+$	PI(42:10)	$C_{51}H_{79}O_{13}P$
		-	975.53691	-	975.53589	975.53594		-	9.6 ±4.2	-	7.0 ±3.5	$[M+K]^+$	PI(42:7)	$C_{51}H_{85}O_{13}P$
		-	945.58269	-	-	945.58274		-	5.1 ±2.0	-	-	$[M+Na]^+$	PI(P-42:6)	$C_{51}H_{87}O_{12}P$
		-	961.57771	-	-	961.57765		-	6.6 ±3.3	-	-	$[M+Na]^+$	PI(42:6)	$C_{51}H_{87}O_{13}P$
Glycerophosphoinositol bisphosphates (PIP2s)														
		-	1035.43722	1035.4373 5	1035.4372 5	1035.4373 0		-	18.7 ±6.3	9.3 ±4.2	11.6 ±5.2	$[M+K]^+$	PIP2(34:1)	$C_{43}H_{83}O_{19}P_3$
Glycerophosphoglycero-phosphoglycerols (cardiolipins)														
		947.50202	947.50209	947.50213	947.50255	947.50212		72.1	146.3	96.5	104.1	$[M+Na]^+$	CL(1\'-	$C_{45}H_{82}O_{15}P_2$

Classification	Electric field (Measured *m/z*)					Calculated *m/z*	Electric field (Average *S/N*, n=3)					Assignment		
	**Matrix coating**	**I**	**II**	**III**	**IV**		**Matrix coating**	**I**	**II**	**III**	**IV**	Ion form	Compound	Molecular formula
	**Spray (3-s)**	Off	On	On	Off		**Spray (3-s)**	Off	On	On	Off			
	**Incubation (60-s)**	Off	On	Off	On		**Incubation (60-s)**	Off	On	Off	On			
	**Drying (90-s)**	Off	On	Off	On		**Drying (90-s)**	Off	On	Off	On			
								±9.8	±14.9	±10.1	±10.5			
		963.47605	963.47608	963.47597	963.47607	963.47605		335.6 ±16.4	1486.4 ±38.8	772.1 ±26.7	866.1 ±28.2	$[M+K]^+$	[18:2(9Z,12Z)/0:0],3\'-[18:2(9Z,12Z)/0:0])	
Cyclic phosphatidic acids (cPAs)														
		415.22203	415.22198	415.22204	415.22208	415.22200		7.2 ±3.3	15.3 ±5.6	9.4 ±4.8	13.7 ±5.2	$[M+Na]^+$	CPA(16:0)	$C_{19}H_{37}O_6P$
		431.19606	431.19601	431.19596	431.19599	431.19593		5.6 ±2.3	18.9 ±6.5	7.7 ±3.4	14.6 ±5.8	$[M+K]^+$		
		455.19598	455.19592	455.19604	455.19593	455.19593		8.6 ±5.5	36.0 ±8.4	21.9 ±6.8	31.7 ±8.1	$[M+K]^+$	CPA(18:2)	$C_{21}H_{37}O_6P$
		441.23764	441.23769	441.23761	441.23758	441.23765		5.0 ±2.1	9.8 ±4.8	6.2 ±3.5	8.5 ±4.6	$[M+Na]^+$	CPA(18:1)	$C_{21}H_{39}O_6P$
		457.21163	457.21167	457.21152	457.21169	457.21158		7.8 ±3.5	31.5 ±8.0	19.4 ±6.6	29.2 ±7.8	$[M+K]^+$		
		443.25328	443.25334	443.25340	443.25334	443.25330		5.3 ±2.3	11.2 ±5.0	7.0 ±3.2	8.8 ±4.7	$[M+Na]^+$	CPA(18:0)	$C_{21}H_{41}O_6P$
		459.22721	459.22719	459.22721	459.22718	459.22723		14.1 ±5.3	31.3 ±8.0	23.3 ±6.7	29.0 ±7.7	$[M+K]^+$		
CDP-Glycerols														
		-	980.53719	-	980.53721	980.53722		-	7.8 ±3.5	-	6.4 ±3.1	$[M+H]^+$	CDP-DG(34:1)	$C_{46}H_{83}N_3O_{15}P_2$
		-	1018.49315	-	101849318	1018.49310		-	10.3 ±5.9	-	8.4 ±5.4	$[M+K]^+$		
		-	982.55286	982.55295	982.55284	982.55287		-	12.0 ±5.0	5.6 ±2.3	7.6 ±3.4	$[M+H]^+$	CDP-DG(34:0)	$C_{46}H_{85}N_3O_{15}P_2$
		-	1020.50867	-	-	1020.50875		-	5.0 ±2.0	-	-	$[M+K]^+$		
		-	1010.58414	-	1010.58419	1010.58417		-	7.8 ±3.5	-	6.0 ±2.8	$[M+H]^+$	CDP-DG(36:0)	$C_{46}H_{89}N_3O_{15}P_2$

Classification	Electric field (Measured *m/z*)					Calculated *m/z*	Electric field (<u>Average *S/N*, n=3)</u>					Assignment		
	**Matrix coating**	**I**	**II**	**III**	**IV**		**Matrix coating**	**I**	**II**	**III**	**IV**	Ion form	Compound	Molecular formula
	**Spray (3-s)**	Off	On	On	Off		**Spray (3-s)**	Off	On	On	Off			
	**Incubation (60-s)**	Off	On	Off	On		**Incubation (60-s)**	Off	On	Off	On			
	**Drying (90-s)**	Off	On	Off	On		**Drying (90-s)**	Off	On	Off	On			
		-	1058.58409	-	-	1058.58417		-	5.4 ±2.5	-	-	$[M+H]^+$	CDP-DG(40:4)	$C_{52}H_{89}N_3O_{15}P_2$
		-	1096.54010	1096.54005	1096.54011	1096.54005		-	10.9 ±4.7	7.9 ±3.6	9.1 ±4.3	$[M+K]^+$		
Glycerophosphate														
		-	467.25331	-	467.25328	467.25330		-	6.3 ±3.0	-	5.0 ±2.2	$[M+Na]^+$	sn-3-O-(geranylgeranyl)glycerol 1-phosphate	$C_{23}H_{41}O_6P$
		-	483.22728	483.22732	483.22731	483.22723		-	29.2 ±7.9	15.9 ±6.5	28.2 ±7.6	$[M+K]^+$		
**Sphingolipids**														
Ceramides (Cers)														
		464.35007	464.35012	464.35002	464.35017	464.35005		5.1 ±2.2	8.7 ±4.1	5.6 ±2.3	6.0 ±2.8	$[M+K]^+$	C-8 Ceramide	$C_{26}H_{51}NO_3$
		602.49102	602.49091	602.49095	602.49086	602.49090		6.7 ±3.1	21.2 ±6.2	14.0 ±5.4	17.8 ±5.7	$[M+K]^+$	Cer(d36:2)	$C_{36}H_{69}NO_3$
		604.50661	604.50663	604.50645	604.50664	604.50655		5.3 ±2.0	10.7 ±4.3	7.9 ±3.3	9.1 ±4.0	$[M+K]^+$	Cer(d36:1)	$C_{36}H_{71}NO_3$
		-	684.47285	-	684.47279	684.47288		-	9.5 ±4.2	-	6.7 ±3.0	$[M+K]^+$	CerP(d36:1)	$C_{36}H_{72}NO_6P$
		632.53793	632.53781	632.53774	632.53784	632.53785		7.5 ±3.6	27.3 ±7.5	18.1 ±5.8	21.1 ±6.3	$[M+K]^+$	Cer(d38:1)	$C_{38}H_{75}NO_3$
		686.58480	686.58486	686.58473	686.58482	686.58480		7.4 ±3.5	16.4 ±5.4	8.4 ±4.0	13.5 ±5.1	$[M+K]^+$	Cer(d42:2)	$C_{42}H_{81}NO_3$
		-	766.55110	766.55116	766.55108	766.55113		-	13.0 ±5.2	8.4 ±4.1	11.6 ±4.6	$[M+K]^+$	CerP(d42:2)	$C_{42}H_{82}NO_6P$
		-	688.60044	-	688.60040	688.60045		-	7.3 ±3.1	-	5.0 ±2.2	$[M+K]^+$	Cer(d42:1)	$C_{42}H_{83}NO_3$
Sphingomyelins (SMs)														
		-	703.57475	703.57490	703.57487	703.57485		-	8.7 ±4.2	5.0 ±2.0	7.3 ±3.4	$[M+H]^+$	SM(d34:1)	$C_{39}H_{79}N_2O_6P$

Classification	Electric field (Measured *m/z*)					Calculated *m/z*	Electric field (Average *S/N*, n=3)					Assignment		
	**Matrix coating**	**I**	**II**	**III**	**IV**		**Matrix coating**	**I**	**II**	**III**	**IV**	Ion form	Compound	Molecular formula
	**Spray (3-s)**	Off	On	On	Off		**Spray (3-s)**	Off	On	On	Off			
	**Incubation (60-s)**	Off	On	Off	On		**Incubation (60-s)**	Off	On	Off	On			
	**Drying (90-s)**	Off	On	Off	On		**Drying (90-s)**	Off	On	Off	On			
		725.55684	725.55673	725.55682	725.55677	725.55680		6.2 ±2.7	24.5 ±6.2	15.4 ±4.2	23.9 ±5.9	$[M+Na]^+$		
		753.58812	753.58805	753.58816	753.58807	753.58810		12.3 ±4.1	22.9 ±5.7	16.7 ±4.5	18.3 ±4.8	$[M+Na]^+$	SM(d36:1)	$C_{41}H_{83}N_2O_6P$
		769.56197	769.56214	769.56207	769.56197	769.56203		86.2 ±9.9	138.3 ±13.6	88.3 ±10.1	103.8 ±12.3	$[M+K]^+$		
		797.59335	797.59341	797.59328	797.59323	797.59333		5.5 ±2.4	9.1 ±5.1	6.2 ±3.8	7.6 ±4.3	$[M+K]^+$	SM(d38:1)	$C_{43}H_{87}N_2O_6P$
		-	787.66879	-	-	787.66875		-	7.3 ±4.2	-	-	$[M+H]^+$	SM(d40:1)	$C_{45}H_{91}N_2O_6P$
		825.62461	825.62470	825.62457	825.62469	825.62463		20.8 ±5.3	40.0 ±7.5	37.9 ±7.1	39.5 ±7.3	$[M+K]^+$		
		-	813.68448	-	813.68438	813.68440		-	12.2 ±5.0	-	7.8 ±3.9	$[M+H]^+$	SM(d42:2)	$C_{47}H_{93}N_2O_6P$
		851.64021	851.64034	851.64026	851.64033	851.64028		7.6 ±3.6	12.2 ±4.1	9.9 ±3.8	11.2 ±4.0	$[M+K]^+$		
		-	815.70001	815.70009	815.70013	815.70005		-	8.0 ±3.4	5.8 ±3.4	7.2 ±3.0	$[M+H]^+$		
		837.68204	837.68202	837.68193	837.68194	837.68200		12.2 ±4.3	28.4 ±5.1	22.6 ±4.8	25.4 ±4.9	$[M+Na]^+$	SM(d42:1)	$C_{47}H_{95}N_2O_6P$
		853.65586	853.65585	853.65584	853.65590	853.65593		6.4 ±3.3	13.7 ±5.2	8.4 ±4.6	11.1 ±5.1	$[M+K]^+$		
Glycosphingolipids														
		500.29845	500.29837	500.29842	500.29836	500.29841		21.2 ±5.2	53.7 ±7.4	32.7 ±6.7	46.1 ±7.0	$[M+K]^+$	Glucosyl sphingosine	$C_{24}H_{47}NO_7$
		-	828.54429	828.54430	828.54435	828.54436		-	16.2 ±5.2	11.9 ±4.1	14.8 ±4.8	$[M+Na]^+$	LacCer(d30:1)	$C_{42}H_{79}NO_{13}$
		766.55930	766.55942	766.55929	766.55938	766.55938		6.7 ±3.4	14.3 ±4.5	9.3 ±4.0	13.2 ±4.2	$[M+K]^+$	GlcCer(d36:1)	$C_{42}H_{81}NO_8$
		-	856.57567	-	-	856.57566		-	7.5 ±3.9	-	-	$[M+Na]^+$	LacCer(d32:1)	$C_{44}H_{83}NO_{13}$
		-	852.58653	-	852.58648	852.58652		-	10.1 ±5.5	-	8.8 ±4.3	$[M+H]^+$	(3'-sulfo)Galβ-Cer(d38:0(2OH	$C_{44}H_{85}NO_{12}S$

Classification	Electric field (Measured *m/z*)					Calculated *m/z*	Electric field (Average *S/N*, n=3)					Assignment		
	**Matrix coating**	**I**	**II**	**III**	**IV**		**Matrix coating**	**I**	**II**	**III**	**IV**	Ion form	Compound	Molecular formula
	**Spray (3-s)**	Off	On	On	Off		**Spray (3-s)**	Off	On	On	Off			
	**Incubation (60-s)**	Off	On	Off	On		**Incubation (60-s)**	Off	On	Off	On			
	**Drying (90-s)**	Off	On	Off	On		**Drying (90-s)**	Off	On	Off	On			
													))	
		794.59074	794.59065	794.59072	794.59071	794.59068		8.5 ±4.5	14.2 ±6.3	10.5 ±5.6	12.7 ±5.9	$[M+K]^+$	GalCer(d38:1)	$C_{44}H_{85}NO_8$
		820.60641	820.60634	820.60635	820.60633	820.60633		70.3 ±11.2	159.0 ±16.3	106.8 ±13.5	126.3 ±14.2	$[M+K]^+$	GlcCer(d40:2)	$C_{46}H_{87}NO_8$
		-	836.60123	-	-	836.60124		-	5.2 ±2.5	-	-	$[M+K]^+$	GlcCer(d16:2/24:0(2OH))	$C_{46}H_{87}NO_9$
		822.62196	822.62202	822.62192	822.62194	822.62198		10.6 ±6.1	17.7 ±8.3	12.6 ±6.3	15.7 ±7.2	$[M+K]^+$	GlcCer(d40:1)	$C_{46}H_{89}NO_8$
		-	928.62121	928.62127	928.62116	928.61220		-	18.6 ±9.4	13.8 ±6.5	16.6 ±8.7	$[M+K]^+$	LacCer(d36:1)	$C_{48}H_{91}NO_{13}$
		832.66372	832.66370	832.66377	832.66365	832.66369		5.9 ±2.8	14.0 ±6.2	9.5 ±5.0	13.5 ±6.1	$[M+Na]^+$	GlcCer(d42:2)	$C_{48}H_{91}NO_8$
		848.63762	848.63761	848.63772	848.63774	848.63763		9.5 ±5.9	49.8 ±13.5	32.2 ±12.1	46.4 ±13.0	$[M+K]^+$		
		-	892.67198	892.67205	892.67197	892.67197		-	8.2 ±5.3	6.3 ±3.4	7.5 ±4.1	$[M+H]^+$	LacCer(d36:0)	$C_{48}H_{93}NO_{13}$
		850.65327	850.65326	850.65332	850.65330	850.65328		5.2 ±2.1	19.2 ±6.7	12.7 ±5.4	16.2 ±6.1	$[M+K]^+$	GlcCer(d42:1)	$C_{48}H_{93}NO_8$
		-	852.66891	-	-	852.66893		-	7.3 ±4.3	-	-	$[M+K]^+$	GlcCer(d42:0)	$C_{48}H_{95}NO_8$
		876.66887	876.66889	876.66883	876.66898	876.66893		26.0 ±13.1	60.9 ±16.1	42.6 ±14.5	54.8 ±15.7	$[M+K]^+$	GlcCer(d44:2)	$C_{50}H_{95}NO_8$
		878.68468	878.68466	878.68449	878.68463	878.68458		5.4 ±2.4	15.5 ±5.8	10.8 ±4.8	13.5 ±5.1	$[M+K]^+$	GlcCer(d44:1)	$C_{50}H_{97}NO_8$
		-	1010.69051	-	-	1010.69045		-	6.5 ±2.8	-	-	$[M+K]^+$	Galβ1-4Glcβ-Cer(d42:2)	$C_{54}H_{101}NO_{13}$
		-	1012.70616	-	-	1012.70610		-	5.6 ±2.3	-	-	$[M+K]^+$	Galβ1-4Glcβ-Cer(d42:1)	$C_{54}H_{103}NO_{13}$
Sphingoid bases														
		-	264.19336	-	-	264.19340		-	6.5 ±3.1	-	-	$[M+Na]^+$	(4E,6E,d14:2) sphingosine	$C_{14}H_{27}NO_2$

Classification	Electric field (Measured *m/z*)					Calculated *m/z*	Electric field (Average *S/N*, n=3)					Assignment		
	**Matrix coating**	**I**	**II**	**III**	**IV**		**Matrix coating**	**I**	**II**	**III**	**IV**	Ion form	Compound	Molecular formula
	**Spray (3-s)**	Off	On	On	Off		**Spray (3-s)**	Off	On	On	Off			
	**Incubation (60-s)**	Off	On	Off	On		**Incubation (60-s)**	Off	On	Off	On			
	**Drying (90-s)**	Off	On	Off	On		**Drying (90-s)**	Off	On	Off	On			
Ceramide phosphoinositols (PI-Cers)														
		-	852.50094	852.49982	852.49988	852.49989		-	13.2 ±4.3	7.8 ±3.7	10.1 ±4.1	$[M+K]^+$	PI-Cer(t34:0(2OH))	$C_{40}H_{80}NO_{13}P$
		838.61681	838.61683	838.61680	838.61682	838.61678		14.0 ±5.1	52.6 ± 9.1	34.7 ±8.4	49.4 ±8.8	$[M+H]^+$	PI-Cer(d38:0)	$C_{44}H_{88}NO_{11}P$
		864.63248	864.63239	864.63237	864.63239	864.63243		63.1 ±13.2	186.1 ±20.1	130.8 ±13.4	160.5 ±17.8	$[M+H]^+$	PI-Cer(d40:10)	$C_{46}H_{90}NO_{11}P$
		866.64815	866.64805	866.64813	866.64808	866.64808		77.1 ±14.3	256.8 ±23.4	196.4 ±21.5	200.5 ±22.0	$[M+H]^+$	PI-Cer(d40:0)	$C_{46}H_{92}NO_{11}P$
		-	904.62494	-	904.62497	904.62494		-	8.9 ±4.4	-	5.0 ±2.3	$[M+Na]^+$	PI-Cer(t40:0)	$C_{46}H_{92}NO_{12}P$
		894.67929	894.67937	894.67942	894.67937	894.67938		5.6 ±2.4	11.6 ±4.2	7.1 ±3.4	9.9 ±4.0	$[M+H]^+$	PI-Cer(d42:0)	$C_{48}H_{96}NO_{11}P$
		-	1154.70930	-	1154.70914	1154.70921		-	8.4 ±4.2	-	6.5 ±3.1	$[M+K]^+$	MIPC(t44:0(2OH))	$C_{56}H_{110}NO_{18}P$
**Neutral Lipids**														
**Glycerolipids**														
Monoacylglycerols (MAGs)														
		369.24012	369.24015	369.24014	369.24011	369.24017		5.4 ±2.3	10.3 ±5.1	8.5 ±3.4	9.7 ±4.5	$[M+K]^+$	MG (16:0)	$C_{19}H_{38}O_4$
		-	379.28181	-	-	379.28188		-	5.1 ±2.0	-	-	$[M+Na]^+$	MG (18:1)	$C_{21}H_{40}O_4$
		-	395.25575	-	-	395.25582		-	5.4 ±2.3	-	-	$[M+K]^+$		
		-	397.27154	-	-	397.27147		-	5.5 ±2.0	-	-	$[M+K]^+$	MG (18:0)	$C_{21}H_{42}O_4$
		-	417.24032	-	-	417.24017		-	5.7 ±2.5	-	-	$[M+K]^+$	MG (20:4)	$C_{23}H_{38}O_4$

Classification		Electric field (Measured *m/z*)				Calculated *m/z*		Electric field (Average *S/N*, n=3)				Assignment		
	**Matrix coating**	**I**	**II**	**III**	**IV**		**Matrix coating**	**I**	**II**	**III**	**IV**	Ion form	Compound	Molecular formula
	**Spray (3-s)**	Off	On	On	Off		**Spray (3-s)**	Off	On	On	Off			
	**Incubation (60-s)**	Off	On	Off	On		**Incubation (60-s)**	Off	On	Off	On			
	**Drying (90-s)**	Off	On	Off	On		**Drying (90-s)**	Off	On	Off	On			
		419.25577	419.25581	419.25576	419.25584	419.25582		5.4 ±2.2	17.3 ±5.8	9.6 ±4.4	12.0 ±5.2	$[M+K]^+$	MG (20:3)	$C_{23}H_{40}O_4$
		-	425.26621	-	-	425.26623		-	5.2 ±2.3	-	-	$[M+Na]^+$	MG (22:6)	$C_{25}H_{38}O_4$
		445.27137	445.27143	445.27146	445.27150	445.27147		5.3 ±2.0	8.0 ±3.2	6.3 ±2.8	7.4 ±3.0	$[M+K]^+$	MG (22:4)	$C_{25}H_{42}O_4$
Diacylglycerols (DAGs)														
		551.50347	551.50335	551.50350	551.50337	551.50339		60.8 ±13.1	287.8 ±16.8	141.0 ±14.0	203.1 ±15.7	$[M+H]^+$		
		-	573.48531	573.48537	573.48529	573.48533		-	9.9 ±4.0	6.5 ±2.9	8.8 ±3.7		DG(P-32:1)	$C_{35}H_{66}O_4$
		-	589.45925	-	589.45918	589.45927		-	7.9 ±3.2	-	6.5 ±2.9			
		607.46996	607.46982	607.46982	607.46976	607.46983		6.7 ±2.7	16.6 ±5.3	8.2 ±3.3	13.6 ±4.8	$[M+K]^+$	DG(32:0)	$C_{35}H_{68}O_5$
		561.52389	561.52406	561.52370	561.52410	561.52412		5.3 ±2.4	7.6 ±3.1	5.8 ±2.6	6.8 ±3.0	$[M+H]^+$	1-tetradecanyl-2-(8-[3]-ladderane-octanyl)-sn-glycerol	$C_{37}H_{68}O_3$
		-	631.47028	-	-	631.46983		-	5.2 ±2.0	-	-	$[M+K]^+$	DG(34:2)	$C_{37}H_{68}O_5$
		633.48562	633.48551	633.48538	633.48549	633.48548		6.4 ±3.1	11.5 ±5.2	7.3 ±4.5	9.4 ±4.9	$[M+K]^+$	DG(34:1)	$C_{37}H_{70}O_5$
		619.50647	619.50645	619.50631	619.50645	619.50622		5.2 ±2.2	7.9 ±3.2	5.9 ±2.6	6.8 ±3.3	$[M+K]^+$	DG(O-34:1)	$C_{37}H_{72}O_4$
		-	635.50130	-	635.50131	635.50113		-	7.6 ±3.1	-	5.1 ±2.3	$[M+K]^+$	DG(34:0)	$C_{37}H_{72}O_5$
		655.46960	655.47014	655.46992	655.46986	655.46983		6.8 ±3.2	16.0 ±5.1	8.5 ±3.4	12.6 ±4.6	$[M+K]^+$	DG(36:4)	$C_{39}H_{68}O_5$
		603.53483	603.53475	603.53445	603.53452	603.53469		27.2 ±8.2	54.1 ±13.2	30.2 ±10.5	44.8 ±12.1	$[M+H]^+$	1-(14-methyl-pentadecanoyl)-2-(8-[3]-	$C_{39}H_{70}O_4$
		[illegible]	[illegible]	[illegible]	[illegible]	[illegible]		6.5	11.6	7.5	8.4	[illegible]		

Classification	Electric field (Measured *m/z*)					Calculated *m/z*	Electric field (Average *S/N*, n=3)					Assignment		
	**Matrix coating**	**I**	**II**	**III**	**IV**		**Matrix coating**	**I**	**II**	**III**	**IV**	Ion form	Compound	Molecular formula
	**Spray (3-s)**	Off	On	On	Off		**Spray (3-s)**	Off	On	On	Off			
	**Incubation (60-s)**	Off	On	Off	On		**Incubation (60-s)**	Off	On	Off	On			
	**Drying (90-s)**	Off	On	Off	On		**Drying (90-s)**	Off	On	Off	On			
													ladderane-octanyl)-sn-glycerol	
		-	657.48551	-	-	657.48548		-	5.3 ±2.4	-	-	[M+K]+	DG(36:3)	$C_{39}H_{70}O_5$
		589.55568	589.55554	589.55533	589.55549	589.55542		5.1 ±2.0	7.9 ±3.3	6.5 ±3.1	7.0 ±3.2	[M+H]+	1-hexadecanyl-2-(8-[3]-ladderane-octanyl)-sn-glycerol	$C_{39}H_{72}O_3$
		-	611.53752	611.53720	611.53741	611.53737		-	7.4 ±3.1	5.2 ±2.1	6.4 ±2.7	[M+Na]+		
		659.50094	659.50127	659.50123	659.50117	659.50113		5.4 ±2.3	10.5 ±4.2	8.4 ±3.5	9.8 ±3.7	[M+K]+	DG(36:2)	$C_{39}H_{72}O_5$
		661.51710	661.51683	661.51665	661.51648	661.51678		5.7 ±2.4	9.4 ±3.7	7.3 ±3.1	8.1 ±3.5	[M+K]+	DG(36:1)	$C_{39}H_{72}O_5$
		-	621.48765	621.48768	621.48770	621.48774		-	7.9 ±3.2	5.3 ±2.2	7.0 ±2.7	[M+H]+	1-(6-[5]-ladderane-hexanoyl)-2-(8-[3]-ladderane-octanyl)-sn-glycerol	$C_{41}H_{64}O_4$
		-	679.47000	-	-	679.46983		-	6.4 ±2.5	-	-	[M+K]+	DG(38:6)	$C_{41}H_{68}O_5$
		-	681.48559	-	-	681.48548		-	6.0 ±2.4	-	-	[M+K]+	DG(38:5)	$C_{41}H_{70}O_5$
		683.50148	683.50110	683.50112	683.50122	683.50113		5.0 ±2.0	8.4 ±3.6	6.1 ±2.4	7.8 ±3.2	[M+K]+	DG(38:4)	$C_{41}H_{72}O_5$
		687.53220	687.53237	687.53233	687.53229	687.53243		6.0 ±2.6	10.3 ±4.2	7.4 ±3.1	9.6 ±4.0	[M+K]+	DG(38:2)	$C_{41}H_{76}O_5$
		689.54823	689.54818	689.54804	689.54811	689.54808		5.2 ±2.0	8.1 ±3.5	7.3 ±3.0	7.6 ±3.2	[M+K]+	DG(38:1)	$C_{41}H_{78}O_5$
		682.45673	682.45669	682.45666	682.45674	682.45677		11.5 ±4.4	24.6 ±6.5	18.7 ±6.1	22.6 ±6.3	[M+Na]+	DG(40:8)	$C_{43}H_{63}D_5O_5$
		-	699.43846	699.43853	699.43846	699.43853		-	9.0 ±3.6	7.8 ±3.2	8.5 ±3.4	[M+K]+	DG(40:10)	$C_{43}H_{64}O_5$

Classification	Electric field (Measured *m/z*)					Calculated *m/z*	Electric field (Average *S/N*, n=3)					Assignment		
	**Matrix coating**	**I**	**II**	**III**	**IV**		**Matrix coating**	**I**	**II**	**III**	**IV**	Ion form	Compound	Molecular formula
	**Spray (3-s)**	Off	On	On	Off		**Spray (3-s)**	Off	On	On	Off			
	**Incubation (60-s)**	Off	On	Off	On		**Incubation (60-s)**	Off	On	Off	On			
	**Drying (90-s)**	Off	On	Off	On		**Drying (90-s)**	Off	On	Off	On			
		649.51920	649.51967	649.51932	649.51942	649.51904		6.7 ±2.8	15.7 ±5.2	9.9 ±4.5	13.4 ±4.7	$[M+H]^+$	1-(8-[5]-ladderane-octanoyl)-2-(8-[3]-ladderane-octanyl)-sn-glycerol	$C_{43}H_{68}O_4$
		-	635.53977	-	635.53959	635.53977		-	7.3 ±3.0	-	6.2 ±2.6	$[M+H]^+$	1-(8-[5]-ladderane-octanyl)-2-(8-[3]-ladderane-octanyl)-sn-glycerol	$C_{43}H_{70}O_3$
		651.53456	651.53471	651.53481	651.53509	651.53469		15.6 ±5.3	52.2 ±9.0	26.4 ±6.8	38.6 ±8.7	$[M+H]^+$	1-(8-[3]-ladderane-octanoyl-2-(8-[3]-ladderane-octanyl)-sn-glycerol	$C_{43}H_{70}O_4$
		707.50137	707.50109	707.50129	707.50138	707.50113		5.3 ±2.2	9.3 ±4.3	6.4 ±2.8	8.0 ±3.4	$[M+K]^+$	DG(40:6)	$C_{43}H_{72}O_5$
		725.45443	725.45413	725.45407	725.45419	725.45418		7.6 ±3.1	15.4 ±5.3	10.8 ±4.2	13.1 ±4.4	$[M+K]^+$	DG(42:11)	$C_{45}H_{66}O_5$
Triradylglycerols (TAGs)														
		-	869.66542	869.66546	869.66533	869.66537		-	11.2 ±4.4	7.6 ±3.1	8.8 ±3.7	$[M+H]^+$	TG(54:11)	$C_{57}H_{88}O_6$
		-	873.69664	-	-	873.69667		-	6.7 ±3.2	-	-	$[M+H]^+$	TG(54:9)	$C_{57}H_{92}O_6$
		-	995.70995	-	-	995.70991		-	5.8 ±2.5	-	-	$[M+Na]^+$	TG(62:15)	$C_{65}H_{96}O_6$
		-	997.72553	-	997.72531	997.72556		-	7.8 ±3.2	-	5.3 ±2.2	$[M+Na]^+$	TG(62:14)	$C_{65}H_{98}O_6$
		-	1035.68388	-	1035.68378	1035.68385		-	11.0 ±4.5	-	6.1 ±3.0	$[M+K]^+$	TG(64:17)	$C_{67}H_{96}O_6$

Classification	Matrix coating	I	II	III	IV	Calculated *m/z*	Matrix coating	I	II	III	IV	Ion form	Compound	Molecular formula
	**Electric field (Measured *m/z*)**						**Electric field (Average *S/N*, n=3)**					**Assignment**		
	**Spray (3-s)**	Off	On	On	Off		**Spray (3-s)**	Off	On	On	Off			
	**Incubation (60-s)**	Off	On	Off	On		**Incubation (60-s)**	Off	On	Off	On			
	**Drying (90-s)**	Off	On	Off	On		**Drying (90-s)**	Off	On	Off	On			
Other Glycerolipids														
		834.62189	834.6281	834.62174	834.62175	834.62183		15.3 ±5.3	46.4 ±12.3	27.6 ±8.4	37.2 ±10.2	$[M+Na]^+$	1-(9Z,1Z-octadecadienoyl)-2-(10Z,13Z,16Z,19Z-docosatetraenoyl)-3-O-[hydroxymethyl-N,N,N-trimethyl-beta-alanine]-glycerol	$C_{50}H_{85}NO_7$
**Sterol Lipids**														
		429.24023	429.24024	429.24022	429.24017	429.24017		6.1 ±2.9	13.6 ±4.3	7.7 ±3.5	10.5 ±4.1	$[M+K]^+$	C24 bile acids and/or its isomers	$C_{24}H_{38}O_4$
		457.27165	457.27148	457.27139	457.27135	457.27147		8.5 ±4.4	31.5 ±7.6	16.4 ±5.4	29.2 ±7.0	$[M+K]^+$	24-northornasterol A	$C_{26}H_{42}O_4$
		-	423.30241	-	-	423.30237		-	5.7 ±2.6	-	-	$[M+K]^+$	Dehydrocholesterol	$C_{27}H_{44}O$
		-	471.28706	-	-	471.28712		-	6.4 ±3.1	-	-	$[M+K]^+$	C27 bile acids and/or its isomers	$C_{27}H_{44}O_4$
		409.34418	409.34412	409.34404	409.34418	409.34409		6.2 ±3.0	15.2 ±5.6	7.4 ±3.4	13.3 ±4.2	$[M+Na]^+$	Cholesterol	$C_{27}H_{46}O$
		425.31836	425.31819	425.31805	425.31808	425.31802		6.0 ±2.8	13.5 ±5.3	7.8 ±3.5	9.7 ±4.5	$[M+K]^+$		
		473.32393	473.32365	473.32378	473.32357	473.32375		5.7	14.7	6.4	8.6	$[M+Na]^+$	C27 bile acids	$C_{27}H_{46}O_5$

Classification	Electric field (Measured *m/z*)					Calculated *m/z*	Electric field (Average *S/N*, n=3)					Assignment		
	**Matrix coating**	**I**	**II**	**III**	**IV**		**Matrix coating**	**I**	**II**	**III**	**IV**	Ion form	Compound	Molecular formula
	**Spray (3-s)**	Off	On	On	Off		**Spray (3-s)**	Off	On	On	Off			
	**Incubation (60-s)**	Off	On	Off	On		**Incubation (60-s)**	Off	On	Off	On			
	**Drying (90-s)**	Off	On	Off	On		**Drying (90-s)**	Off	On	Off	On			
								±2.5	±5.8	±3.0	±4.4		and/ or its isomers	
		-	489.31863	-	-	489.31866		-	6.0 ±2.8	-	-	$[M+Na]^+$	C27 bile acids and/ or its isomers	$C_{27}H_{46}O_6$
		485.30306	485.30276	485.30275	485.30286	485.30277		5.5 ±2.4	12.5 ±5.0	7.8 ±3.5	10.3 ±4.8	$[M+K]^+$	Ergosterols and C24-methyl derivatives	$C_{28}H_{46}O_4$
		-	431.32845	-	431.32866	431.32844		-	6.4 ±3.1	-	5.6 ±2.5	$[M+Na]^+$	Conicasterol B	$C_{29}H_{44}O$
		497.33906	497.33934	497.33920	497.33919	497.33915		6.4 ±3.0	10.6 ±4.7	7.3 ±3.4	9.8 ±4.6	$[M+K]^+$	C30 isoprenoids	$C_{30}H_{50}O_3$
		-	777.41860	-	-	777.41859		-	5.4 ±2.3	-	-	$[M+K]^+$	Spirostanols and/ or its isomers	$C_{40}H_{66}O_{12}$
		-	827.41898	827.41886	827.41890	827.41898		-	11.9 ±4.8	7.9 ±3.5	9.5 ±4.6	$[M+K]^+$	Spirostanols and/ or its isomers	$C_{40}H_{68}O_{15}$
**Prenol Lipids**														
		445.29251	445.29253	445.29231	445.29241	445.29245		5.0 ±2.0	7.2 ±4.4	5.3 ±2.3	5.7 ±2.5	$[M+Na]^+$	19-(3-methyl-butanoyloxy)-villanovane-13alpha,17-diol	$C_{25}H_{42}O_5$
**Fatty acyls**														
Fatty acids (FAs)														
		-	319.20342	-	-	319.20339		-	5.0 ±2.3	-	-	$[M+K]^+$	FA(18:2)	$C_{18}H_{32}O_2$
		321.21914	321.21922	321.21903	321.21924	321.21904		5.4 ±2.2	15.0 ±6.0	8.7 ±4.2	10.9 ±5.3	$[M+K]^+$	FA(18:1)	$C_{18}H_{34}O_2$

Classification		Electric field (Measured *m/z*)				Calculated *m/z*		Electric field (Average *S/N*, n=3)				Assignment		
	**Matrix coating**	**I**	**II**	**III**	**IV**		**Matrix coating**	**I**	**II**	**III**	**IV**	Ion form	Compound	Molecular formula
	**Spray (3-s)**	Off	On	On	Off		**Spray (3-s)**	Off	On	On	Off			
	**Incubation (60-s)**	Off	On	Off	On		**Incubation (60-s)**	Off	On	Off	On			
	**Drying (90-s)**	Off	On	Off	On		**Drying (90-s)**	Off	On	Off	On			
		343.20308	343.20334	343.20335	343.20345	343.20339		6.0 ±2.7	20.2 ±6.0	11.4 ±4.7	19.1 ±5.6	$[M+K]^+$	FA(20:4)	$C_{20}H_{32}O_2$
		367.20339	367.20341	367.20332	367.20339	367.20339		5.4 ±2.3	10.1 ±5.2	8.5 ±5.3	9.7 ±5.1	$[M+K]^+$	FA(22:6)	$C_{22}H_{32}O_2$
		-	393.29748	393.29743	393.29776	393.29753		-	21.6 ±6.5	8.8 ±5.6	18.0 ±5.8	$[M+Na]^+$	FA(22:0)	$C_{22}H_{42}O_4$
		409.27132	409.27137	409.27160	409.27133	409.27147		7.2 ±3.3	23.9 ±6.3	11.4 ±5.7	15.9 ±6.0	$[M+K]^+$		
		465.33448	465.33412	465.33406	465.33421	465.33407		13.2 ±5.3	23.2 ±6.2	18.8 ±5.6	21.1 ±6.2	$[M+K]^+$	FA(26:0)	$C_{26}H_{50}O_4$
**Other compounds**														
		322.05479	322.05487	322.05479	322.05464	322.05483		12.3 ±5.1	28.3 ±6.6	19.3 ±5.7	27.7 ±6.4	$[M+K]^+$	Guanosine	$C_{10}H_{13}N_5O_5$
		-	327.03528	-	-	327.03526		-	6.0 ±2.6	-	-	$[M+Na]^+$	Thymidine 3,5-cyclic monophosphate	$C_{10}H_{13}N_2O_7P$
		352.04164	352.04185	352.04170	352.04169	352.04174		5.2 ±2.3	12.0 ±6.0	8.5 ±5.5	10.8 ±5.4	$[M+Na]^+$	Cyclic adenosine monophosphate (cAMP)	$C_{10}H_{12}N_5O_6P$
		368.01546	368.01564	368.01581	368.01559	368.01568		5.8 ±2.5	20.1 ±7.1	11.9 ±5.8	17.4 ±6.5	$[M+K]^+$		
		-	1146.50845	-	1146.50857	1146.50865		-	8.4 ±5.4	-	6.3 ±2.8	$[M+H]^+$	CoA(26:0)	$C_{47}H_{86}N_7O_{17}P_3S$
		-	1168.49036	-	1168.49027	1168.49060		-	6.5 ±2.9	-	5.3 ±2.4	$[M+Na]^+$		

Note: n=3 means triplicate tissue slides; the means reflect nine mass spectra acquired from the triplicate tissue slides that were mounted on three ITO-coated microscopic glass slides.

Supplementary Information Table S2. Comparison of lipid detection on rat brain sections by MALDI-FTICR MS in the positive-ion mode using MCAEF and standard spray methods for quercetin coating, respectively.

Classification	No.	Measured *m/z*		Calculated *m/z*	Error (ppm)		Assignment			Ref.	Structurally specific CID ions (*m/z*)$^{a)}$
		MCAEF	Standard spray		MCAEF	Common spray	Ion form	Compound	Molecular formula		
**Glycerophospholipids**											
Phosphatidylcholines (PCs)											
	1	478.32944	478.32921	478.32920	0.50	0.02	$[M+H]^+$	PC(O-16:2)	$C_{24}H_{48}NO_6P$		
		500.31143	500.31090	500.31115	-0.56	0.50	$[M+Na]^+$				
		516.28531	516.28499	516.28508	0.45	-0.17	$[M+K]^+$				
	2	502.32660	-	502.32680	-0.40	-	$[M+Na]^+$	PC(O-16:1)	$C_{24}H_{50}NO_6P$		
		518.30102	518.30067	518.30073	0.56	-0.12	$[M+K]^+$				
	3	496.33958	496.33925	496.33977	-0.38	0.06	$[M+H]^+$	PC(16:0)	$C_{24}H_{50}NO_7P$		104, 184, 478, 496
		534.29588	534.29559	534.29565	0.43	-0.11	$[M+K]^+$			17	
	4	504.34249	-	504.34245	0.08	-	$[M+Na]^+$	PC(O-16:0)	$C_{24}H_{52}NO_6P$		
	5	516.30896	516.30887	516.30847	0.95	0.77	$[M+H]^+$	PC(18:4)	$C_{26}H_{46}NO_7P$		
	6	518.32450	-	518.32412	0.73	-	$[M+H]^+$	PC(18:3)	$C_{26}H_{48}NO_7P$		
	7	506.36069	506.36056	506.36050	0.38	0.12	$[M+H]^+$	PC(P-18:1)	$C_{26}H_{52}NO_6P$		
	8	528.34262	528.34236	528.34245	0.32	-0.17	$[M+Na]^+$	PC(O-18:2)	$C_{26}H_{52}NO_6P$		
		544.31646	544.31639	544.31638	0.15	0.02	$[M+K]^+$				
	9	522.35543	-	522.35542	0.02	-	$[M+H]^+$	PC(18:1)	$C_{26}H_{52}NO_7P$		104, 184, 504, 522
		560.31143	560.31123	560.31130	0.23	-0.12	$[M+K]^+$			17	
	10	524.37155	524.37117	524.37107	0.92	0.19	$[M+H]^+$	PC(18:0)	$C_{26}H_{54}NO_7P$	18	104, 184, 506, 524
		562.32725	562.32677	562.32695	0.53	-0.32	$[M+K]^+$			17	
	11	544.33975	544.33970	544.33977	-0.04	-0.13	$[M+H]^+$	PC(20:4)	$C_{28}H_{50}NO_7P$		104, 184, 526, 544
		582.29603	-	582.29565	0.65	-	$[M+K]^+$				
	12	546.35543	-	546.35542	0.02	-	$[M+H]^+$	PC(20:3)	$C_{28}H_{52}NO_7P$		
	13	548.37134	548.37142	548.37107	0.49	0.64	$[M+H]^+$	PC(20:2)	$C_{28}H_{54}NO_7P$		
		586.32721	586.32713	586.32695	0.44	0.31	$[M+K]^+$				
	14	602.32135	602.32227	602.32186	-0.85	0.68	$[M+K]^+$	PC(20:1)	$C_{28}H_{54}NO_8P$		
	15	604.33734	604.33764	604.33751	-0.28	0.22	$[M+K]^+$	PC(20:0)	$C_{28}H_{56}NO_8P$		

Classification	No.	Measured *m/z*		Calculated *m/z*	Error (ppm)		Assignment			Ref.	Structurally specific CID ions (*m/z*)[a)]
		MCAEF	Standard spray		MCAEF	Common spray	Ion form	Compound	Molecular formula		
	16	606.29509	606.29527	606.29565	-0.92	-0.63	$[M+K]^+$	PC(22:6)	$C_{30}H_{50}NO_7P$	17	
	17	608.31094	-	608.31130	-0.59	-	$[M+K]^+$	LysoPC(22:5)	$C_{30}H_{52}NO_7P$		
	18	610.32647	610.32706	610.32695	-0.79	0.18	$[M+K]^+$	PC(22:4)	$C_{30}H_{54}NO_7P$		
	19	614.35804	614.35835	614.35825	-0.34	0.16	$[M+K]^+$	PC(22:2)	$C_{30}H_{58}NO_7P$		
	20	616.37402	616.37398	616.37390	0.19	0.13	$[M+K]^+$	PC(22:1)	$C_{30}H_{60}NO_7P$		
	21	618.38923	618.38967	618.38955	-0.52	0.19	$[M+K]^+$	PC(22:0)	$C_{30}H_{62}NO_7P$		
	22	644.40554	644.40537	644.40520	0.53	0.26	$[M+K]^+$	LysoPC(24:1)	$C_{32}H_{64}NO_7P$		
	23	646.42107	646.42079	646.42085	0.34	-0.09	$[M+K]^+$	PC(24:0)	$C_{32}H_{66}NO_7P$		
	24	648.43642	648.43664	648.43650	-0.12	0.22	$[M+K]^+$	LysoPC(26:1)	$C_{32}H_{68}NO_7P$		
	25	650.45257	650.45234	650.45215	0.65	0.29	$[M+K]^+$	LysoPC(26:0)	$C_{32}H_{70}NO_7P$		
	26	704.52283	704.52246	704.52248	0.50	-0.03	$[M+H]^+$	PC(30:1)	$C_{38}H_{74}NO_8P$		
	27	744.49463	744.49457	744.49401	0.83	0.75	$[M+K]^+$	PC(30:0)	$C_{38}H_{76}NO_8P$		
	28	766.47843	766.47811	766.47836	0.09	-0.33	$[M+K]^+$	PC(32:3)	$C_{40}H_{74}NO_8P$		
	29	770.51011	770.50981	770.50966	0.58	0.19	$[M+K]^+$	PC(32:1)	$C_{40}H_{78}NO_8P$		104, 184, 476, 732
	30	734.57001	734.56974	734.56943	0.79	0.42	$[M+H]^+$	PC(32:0)	$C_{40}H_{80}NO_8P$	19-23	104, 147, 163, 184, 478, 735
		756.55118	756.55161	756.55138	-0.26	0.30	$[M+Na]^+$			20, 22, 24-26	
		772.52504	772.52537	772.52531	-0.35	0.08	$[M+K]^+$			17, 20, 22, 24, 27	
	31	790.47857	790.47818	790.47836	0.27	-0.23	$[M+K]^+$	PC(34:5)	$C_{42}H_{74}NO_8P$		
	32	792.49424	792.49398	792.49401	0.29	-0.04	$[M+K]^+$	PC(34:4)	$C_{42}H_{76}NO_8P$		
	33	794.50967	-	794.50966	0.38	-0.01	$[M+K]^+$	PC(34:3)	$C_{42}H_{78}NO_8P$		
	34	796.52530	-	796.52531	0.90	-0.01	$[M+K]^+$	PC(34:2)	$C_{42}H_{80}NO_8P$		184, 758
	35	760.58475	760.58524	760.58508	-0.43	0.21	$[M+H]^+$	PC(34:1)	$C_{42}H_{82}NO_8P$	17-20, 22, 28-31	86, 184, 577, 701, 761
		782.56690	782.56776	782.56703	-0.17	0.93	$[M+Na]^+$			22, 26	

Classification	No.	Measured m/z		Calculated m/z	Error (ppm)		Assignment			Ref.	Structurally specific CID ions (m/z)[a)]
		MCAEF	Standard spray		MCAEF	Common spray	Ion form	Compound	Molecular formula		
		798.54062	798.54057	798.54096	-0.43	-0.49	$[M+K]^+$			17-20, 22, 24-27, 30, 32	
	36	762.60067	-	762.60073	-0.08	-	$[M+H]^+$	PC(34:0)	$C_{42}H_{84}NO_8P$	22, 23	163, 184, 762
		784.58279	-	784.58268	0.14	-	$[M+Na]^+$			22, 26	
		800.55681	-	800.55661	0.25	-	$[M+K]^+$			26	
	37	804.55102	-	804.55138	-0.45	-	$[M+Na]^+$	PC(36:4)	$C_{44}H_{80}NO_8P$		184, 783
		820.52564	820.52528	820.52531	0.40	-0.04	$[M+K]^+$			17, 25, 27, 28, 32	
	38	822.54083	-	822.54096	-0.16	-	$[M+K]^+$	PC(36:3)	$C_{44}H_{82}NO_8P$		184, 785
	39	792.56609	792.56663	792.56678	-0.87	-0.19	$[M+K]^+$	1-hexadecanyl-2-(8-[3]-ladderane-octanyl)-sn-glycerophosphocholine	$C_{44}H_{84}NO_6P$	17	184, 754
	40	808.58219	808.58242	808.58268	-0.61	-0.32	$[M+Na]^+$	PC(36:2)	$C_{44}H_{84}NO_8P$	17, 18, 22	184, 787
		824.55651	824.55618	824.55661	-0.12	-0.52	$[M+K]^+$			18, 28, 32	
	41	810.57727	-	810.57735	-0.10	-	$[M+K]^+$	PC(P-36:1)	$C_{44}H_{86}NO_7P$		
	42	788.61632	-	788.61638	-0.08	-	$[M+H]^+$	PC(36:1)	$C_{44}H_{86}NO_8P$	22, 23	184, 789
		826.57280	826.57220	826.57226	0.65	-0.07	$[M+K]^+$			17, 19, 27, 31, 33	
	43	828.58799	828.58806	828.58791	0.10	0.18	$[M+K]^+$	PC(36:0)	$C_{44}H_{88}NO_8P$		
	44	786.54364	786.54376	786.54322	0.53	0.69	$[M+H]^+$	1-(6-[5]-ladderane-hexanoyl)-2-(8-[3]-ladderane-octanyl)-sn-glycerophosphocholine	$C_{46}H_{76}NO_7P$		
	45	844.52562	844.52571	844.52531	0.37	0.47	$[M+K]^+$	PC(38:6)	$C_{46}H_{80}NO_8P$	17, 22, 24, 27, 28, 34	
	46	846.54098	846.54121	846.54096	0.02	0.30	$[M+K]^+$	PC(38:5)	$C_{46}H_{82}NO_8P$	17, 27	184, 627, 750, 809
	47	810.60115	810.60045	810.60073	0.52	-0.35	$[M+H]^+$	PC(38:4)	$C_{46}H_{84}NO_8P$	18, 22, 23, 28	184, 627, 752, 811
		832.58253	832.58284	832.58268	-0.18	0.19	$[M+Na]^+$			17, 18, 22, 35	

Classification	No.	Measured *m/z*		Calculated *m/z*	Error (ppm)		Assignment			Ref.	Structurally specific CID ions (*m/z*)[a)]
		MCAEF	Standard spray		MCAEF	Common spray	Ion form	Compound	Molecular formula		
		848.55675	848.55723	848.55661	0.16	0.73	$[M+K]^+$			18, 25, 27	
	48	850.57247	850.57524	850.57226	0.25	-0.02	$[M+K]^+$	PC(38:3)	$C_{46}H_{86}NO_8P$		
	49	854.60371	854.60387	854.60356	0.18	0.36	$[M+K]^+$	PC(38:1)	$C_{46}H_{90}NO_8P$		
	50	840.62426	-	840.62430	-0.05	-	$M+K]^+$	PC(P-38:0)	$C_{46}H_{92}NO_7P$		
	51	856.61945	856.61947	856.61921	0.28	0.30	$M+K]^+$	PC(38:0)	$C_{46}H_{92}NO_8P$		
	52	864.49419	-	864.49401	0.21	-	$[M+K]^+$	PC(40:10)	$C_{48}H_{76}NO_8P$		
	53	866.50959	-	866.50966	-0.08	-	$[M+K]^+$	PC(40:9)	$C_{48}H_{78}NO_8P$		
	54	852.53071	-	852.53040	0.36	-	$[M+K]^+$	1-(8-[5]-ladderane-octanoyl)-2-(8-[3]-ladderane-octanyl)-sn-glycerophosphocholine	$C_{48}H_{80}NO_7P$		
	55	870.54027	870.54121	870.54096	-0.79	0.29	$[M+K]^+$	PC(40:7)	$C_{48}H_{82}NO_8P$	27	
	56	856.58277	856.58214	856.58268	0.11	-0.63	$[M+Na]^+$	PC(40:6)	$C_{48}H_{84}NO_8P$	17, 24, 28	86, 184, 776, 834
		872.55643	872.55660	872.55661	-0.21	-0.01	$[M+K]^+$			24, 27	
	57	874.57191	874.57235	874.57226	-0.40	0.10	$[M+K]^+$	PC(40:5)	$C_{48}H_{86}NO_8P$		86, 184, 778, 836
	58	876.58767	876.58740	876.58791	-0.27	-0.58	$[M+K]^+$	PC(40:4)	$C_{48}H_{88}NO_8P$		86, 184, 780, 838
	59	880.61923	-	880.61921	0.02	-	$[M+K]^+$	PC(40:2)	$C_{48}H_{92}NO_8P$		
	60	882.63453	882.63526	882.63486	-0.37	0.45	$[M+K]^+$	PC(40:1)	$C_{48}H_{94}NO_8P$		
	61	906.63465	906.63497	906.63486	-0.23	0.12	$[M+K]^+$	PC(42:3)	$C_{50}H_{94}NO_8P$		
	62	908.65023	-	908.65051	-0.31	-	$[M+K]^+$	PC(42:2)	$C_{50}H_{96}NO_8P$		
	63	910.66639	-	910.66616	0.25	-	$[M+K]^+$	PC(42:1)	$C_{50}H_{98}NO_8P$		
	64	936.68227	-	936.68181	0.49	-	$[M+K]^+$	PC(44:2)	$C_{52}H_{100}NO_8P$		
	65	956.65043	-	956.65051	-0.08	-	$[M+K]^+$	PC(46:6)	$C_{54}H_{96}NO_8P$		
Phosphatidylethanolamines (PEs)											
	1	476.25387	476.25392	476.25378	0.19	0.29	$[M+K]^+$	PE(P-16:0)	$C_{21}H_{44}NO_6P$		
	2	490.23327	490.23326	490.23305	0.45	0.43	$[M+K]^+$	PE(16:1)	$C_{21}H_{42}NO_7P$		
	3	492.24870	-	492.24870	0.00	-	$[M+K]^+$	PE(16:0)	$C_{21}H_{44}NO_7P$		
	4	514.23314	514.23336	514.23305	0.18	0.60	$[M+K]^+$	PE(18:3)	$C_{23}H_{42}NO_7P$		
	5	516.24847	516.24887	516.24870	-0.45	0.33	$[M+K]^+$	PE(18:2)	$C_{23}H_{44}NO_7P$		

Classification	No.	Measured *m/z*		Calculated *m/z*	Error (ppm)		Assignment			Ref.	Structurally specific CID ions (*m/z*)[a)]
		MCAEF	Standard spray		MCAEF	Common spray	Ion form	Compound	Molecular formula		
	6	518.26421	518.26456	518.26435	-0.27	0.41	$[M+K]^+$	PE(18:1)	$C_{23}H_{46}NO_7P$		155, 265, 308, 339, 462, 480
	7	504.28529	504.28536	504.28508	0.41	056	$[M+K]^+$	PE(P-18:0)	$C_{23}H_{48}NO_6P$		267, 403, 462
	8	520.28006	520.28034	520.28000	0.12	0.65	$[M+K]^+$	PE(18:0)	$C_{23}H_{48}NO_7P$		140, 153, 196, 214, 283, 419, 437, 480
	9	540.24891	-	540.24870	0.39	-	$[M+K]^+$	PE(20:4)	$C_{25}H_{44}NO_7P$		153, 195, 259, 303, 439, 500
	10	542.26438	-	542.26435	0.06	-	$[M+K]^+$	PE(20:3)	$C_{25}H_{46}NO_7P$		
	11	544.28009	544.27983	544.28000	0.17	-0.31	$[M+K]^+$	PE(20:2)	$C_{25}H_{48}NO_7P$		
	12	546.29566	546.29528	546.29565	0.02	-0.68	$[M+K]^+$	PE(20:1)	$C_{25}H_{50}NO_7P$		
	13	510.35562	510.35532	510.35542	0.39	-0.20	$[M+H]^+$	PE(20:0)	$C_{25}H_{52}NO_7P$		
		548.31180	-	548.31130	0.91	-	$[M+K]^+$				
	14	564.24874	564.24855	564.24870	0.07	-0.27	$[M+K]^+$	PE(22:6)	$C_{27}H_{44}NO_7P$		
	15	568.27959	568.28031	568.28000	-0.72	0.55	$[M+K]^+$	PE(22:4)	$C_{27}H_{48}NO_7P$		
	16	572.31115	572.31156	572.31130	-0.26	0.45	$[M+K]^+$	PE(22:2)	$C_{27}H_{52}NO_7P$		
	17	574.32675	574.32714	574.32695	-0.35	0.33	$[M+K]^+$	PE(22:1)	$C_{27}H_{54}NO_7P$		
	18	538.38622	-	538.38672	-0.93	-	$[M+H]^+$	PE(22:0)	$C_{27}H_{56}NO_7P$		
		560.36859	-	560.36866	-0.12	-	$[M+Na]^+$				
	19	602.35803	602.35847	602.35825	-0.37	0.37	$[M+K]^+$	LysoPE(24:1)	$C_{29}H_{58}NO_7P$		
	20	644.36862	-	644.36881	-0.29	-	$[M+K]^+$	PE(26:1)	$C_{31}H_{60}NO_8P$		
	21	646.38438	646.38471	646.38446	-0.12	0.39	$[M+K]^+$	PE(26:0)	$C_{31}H_{62}NO_8P$		
	22	756.49369	756.49357	756.49401	-0.42	-0.58	$[M+K]^+$	PE(34:1)	$C_{39}H_{76}NO_8P$		
	23	740.49921	740.49934	740.49910	0.15	0.32	$[M+K]^+$	PE(P-34:1)	$C_{39}H_{76}NO_7P$		
	24	742.51414	-	742.51475	-0.82	-	$[M+K]^+$	PE(P-34:0)	$C_{39}H_{78}NO_7P$		
	25	750.44734	750.44725	750.44706	0.37	0.25	$[M+K]^+$	PE(34:4)	$C_{39}H_{70}NO_8P$		
	26	758.51000	758.51025	758.50966	0.45	0.78	$[M+K]^+$	PE(34:0)	$C_{39}H_{78}NO_8P$		
	27	764.49904	764.49935	764.49910	-0.08	0.33	$[M+K]^+$	PE(P-36:3)	$C_{41}H_{76}NO_7P$		
	28	780.49412	780.49434	780.49401	0.14	0.42	$[M+K]^+$	PE(36:3)	$C_{41}H_{76}NO_8P$		
	29	782.50982	-	782.50966	-0.20	-	$[M+K]^+$	PE(36:2)	$C_{41}H_{78}NO_8P$		

Classification	No.	Measured *m/z*		Calculated *m/z*	Error (ppm)		Assignment			Ref.	Structurally specific CID ions (*m/z*)[a]
		MCAEF	Standard spray		MCAEF	Common spray	Ion form	Compound	Molecular formula		
	30	768.53053	768.53035	768.53040	0.17	-0.07	$[M+K]^+$	PE(P-36:1)	$C_{41}H_{80}NO_7P$		
	31	784.52570	784.52487	784.52531	0.50	-0.56	$[M+K]^+$	PE(36:1)	$C_{41}H_{80}NO_8P$		
	32	770.54624	770.54633	770.54605	0.25	0.36	$[M+K]^+$	PE(P-36:0)	$C_{41}H_{82}NO_7P$		
	33	748.58529	748.58529	748.58508	0.28	0.28	$[M+H]^+$	PE(36:0)	$C_{41}H_{82}NO_8P$		607, 748
	34	786.48354	786.48345	786.48345	0.11	0.00	$[M+K]^+$	PE(P-38:6)	$C_{43}H_{74}NO_7P$		
	35	802.47840	802.47873	802.47836	0.05	0.46	$[M+K]^+$	PE(38:6)	$C_{43}H_{74}NO_8P$		
	36	788.49835	788.49860	788.49910	-0.95	-0.63	$[M+K]^+$	PE(P-38:5)	$C_{43}H_{76}NO_7P$		
	37	804.49421	804.49397	804.49401	0.25	-0.05	$[M+K]^+$	PE(38:5)	$C_{43}H_{76}NO_8P$		
	38	790.51488	790.51451	790.51475	0.16	-0.30	$[M+K]^+$	PE(P-38:4)	$C_{43}H_{78}NO_7P$		
	39	806.50991	806.50956	806.50966	0.31	-0.12	$[M+K]^+$	PE(38:4)	$C_{43}H_{78}NO_8P$		341, 627, 768 or 259, 283, 303, 462, 480, 482, 500, 767
	40	792.53052	-	792.53040	0.15	-	$[M+K]^+$	PE(P-38:3)	$C_{43}H_{80}NO_7P$		
	41	810.54083	-	810.54096	-0.16	-	$[M+K]^+$	PE(38:2)	$C_{43}H_{82}NO_8P$		
	42	774.60067	774.60072	774.60073	-0.08	-0.01	$[M+H]^+$	PE(38:1)	$C_{43}H_{84}NO_8P$		
		812.55688	812.55651	812.55661	0.33	-0.12	$[M+K]^+$				
	43	812.49979	812.49973	812.49910	0.85	0.78	$[M+K]^+$	PE(P-40:7)	$C_{45}H_{76}NO_7P$		
	44	828.49435	-	828.49401	0.41	-	$[M+K]^+$	PE(40:7)	$C_{45}H_{76}NO_8P$		
	45	814.51441	814.51423	814.51475	-0.42	-0.64	$[M+K]^+$	PE(P-40:6)	$C_{45}H_{78}NO_7P$		
	46	830.50977	830.50921	830.50966	0.13	-0.54	$[M+K]^+$	PE(40:6)	$C_{45}H_{78}NO_8P$		
	47	816.53009	816.53073	816.53040	-0.38	0.40	$[M+K]^+$	PE(P-40:5)	$C_{45}H_{80}NO_7P$		
	48	832.52507	-	832.52531	-0.29	-	$[M+K]^+$	PE(40:5)	$C_{45}H_{80}NO_8P$		
	49	818.54557	818.54653	818.54605	-0.59	0.59	$[M+K]^+$	PE(P-40:4)	$C_{45}H_{82}NO_7P$		
	50	834.54025	834.54078	834.54096	-0.85	-0.22	$[M+K]^+$	PE(40:4)	$C_{45}H_{82}NO_8P$		
	51	802.63128	802.63127	802.63203	-0.93	-0.95	$[M+H]^+$	PE(40:1)	$C_{45}H_{88}NO_8P$		
	52	850.47870	-	850.47836	0.40	-	$[M+K]^+$	PE(42:10)	$C_{47}H_{74}NO_8P$		
	53	852.49475	852.49450	852.49401	0.87	0.57	$[M+K]^+$	PE(42:9)	$C_{47}H_{76}NO_8P$		
	54	854.51013	-	854.50966	0.55	-	$[M+K]^+$	PE(42:8)	$C_{47}H_{78}NO_8P$		
	55	856.52505	-	856.52531	-0.30	-	$[M+K]^+$	PE(42:7)	$C_{47}H_{80}NO_8P$		
	56	858.54080	-	858.54096	-0.19	-	$[M+K]^+$	PE(42:6)	$C_{47}H_{82}NO_8P$		

Classification	No.	Measured *m/z*		Calculated *m/z*	Error (ppm)		Assignment			Ref.	Structurally specific CID ions (*m/z*)[a)]
		MCAEF	Standard spray		MCAEF	Common spray	Ion form	Compound	Molecular formula		
	57	824.61619	-	824.61638	-0.23	-	$[M+H]^+$	PE(42:4)	$C_{47}H_{86}NO_8P$		
	58	810.63704	810.63736	810.63712	-0.10	0.30	$[M+H]^+$	PE(O-42:4)	$C_{47}H_{88}NO_7P$		
	59	864.58775	864.58803	864.58791	-0.19	0.14	$[M+K]^+$	PE(42:3)	$C_{47}H_{88}NO_8P$		
	60	850.60853	850.60840	850.60865	-0.14	-0.29	$[M+K]^+$	PE(P-42:2)	$C_{47}H_{90}NO_7P$		
	61	845.67436	845.67442	845.67423	0.15	0.22	$[M+Na]^+$	PE(42:2)	$C_{47}H_{90}NO_8P$		
	62	852.62425	-	852.62430	-0.06	-	$[M+K]^+$	PE(P-42:1)	$C_{47}H_{92}NO_7P$		
	63	868.61934	868.61952	868.61921	0.15	0.36	$[M+K]^+$	PE(42:1)	$C_{47}H_{92}NO_8P$		
	64	870.63471	870.63493	870.63486	-0.17	0.08	$[M+K]^+$	PE(42:0)	$C_{47}H_{94}NO_8P$		
	65	878.50911	-	878.50966	-0.63	-	$[M+K]^+$	PE(44:10)	$C_{49}H_{78}NO_8P$		
	66	880.52546	-	880.52531	0.17	-	$[M+K]^+$	PE(44:9)	$C_{49}H_{80}NO_8P$		
	67	886.57238	886.57251	886.57226	0.14	0.28	$[M+K]^+$	PE(44:6)	$C_{49}H_{86}NO_8P$		
	68	888.58780	888.58817	888.58791	-0.12	0.29	$[M+K]^+$	PE(44:5)	$C_{49}H_{88}NO_8P$		
	69	896.65061	-	896.65051	0.11	-	$[M+K]^+$	PE(44:1)	$C_{49}H_{96}NO_8P$		
Phosphatidic acids (PAs)											
	1	475.22231	475.22224	475.22215	0.34	0.19	$[M+K]^+$	PA(18:1)	$C_{21}H_{41}O_7P$		79, 153, 171, 283, 437
	2	477.23744	477.23741	477.23780	-0.75	-0.82	$[M+K]^+$	PA(18:0)	$C_{21}H_{43}O_7P$		
	3	497.20674	497.20681	497.20650	0.48	0.62	$[M+K]^+$	PA(20:4)	$C_{23}H_{39}O_7P$		153, 171, 259, 303, 457
	4	499.22225	499.22247	499.22215	0.20	0.64	$[M+K]^+$	PA(20:3)	$C_{23}H_{41}O_7P$		
	5	501.23795	501.23790	501.23780	0.30	0.20	$[M+K]^+$	PA(20:2)	$C_{23}H_{43}O_7P$		
	6	487.27974	487.27973	487.27951	0.45	0.45	$[M+Na]^+$	PA(20:1)	$C_{23}H_{45}O_7P$		
		503.25357	503.25347	503.25345	0.24	0.04	$[M+K]^+$				
	7	525.23767	525.23791	525.23780	-0.25	0.21	$[M+K]^+$	PA(22:4)	$C_{25}H_{43}O_7P$		
	8	531.28493	531.28481	531.28475	0.34	0.11	$[M+K]^+$	PA(22:1)	$C_{25}H_{49}O_7P$		
	9	533.30057	533.30061	533.30040	0.32	0.39	$[M+K]^+$	PA(22:0)	$C_{25}H_{51}O_7P$		
	10	679.37367	679.37382	679.37356	0.16	0.38	$[M+K]^+$	PA(32:4)	$C_{35}H_{61}O_8P$		
	11	681.38952	681.38945	681.38921	0.45	0.35	$[M+K]^+$	PA(32:3)	$C_{35}H_{63}O_8P$		
	12	683.40493	683.40504	683.40486	0.10	0.26	$[M+K]^+$	PA(32:2)	$C_{35}H_{65}O_8P$		
	13	685.42113	685.42092	685.42051	0.90	0.60	$[M+K]^+$	PA(32:1)	$C_{35}H_{67}O_8P$		
	14	687.43633	687.43577	687.43616	0.25	-0.57	$[M+K]^+$	PA(32:0)	$C_{35}H_{69}O_8P$		

Classification	No.	Measured m/z		Calculated m/z	Error (ppm)		Assignment			Ref.	Structurally specific CID ions (m/z)[a)]
		MCAEF	Standard spray		MCAEF	Common spray	Ion form	Compound	Molecular formula		
	15	643.50371	643.50361	643.50370	0.02	-0.14	$[M+Na]^+$	PA(O-32:0)	$C_{35}H_{73}O_6P$		
	16	709.42087	-	709.42051	0.51	-	$[M+K]^+$	PA(34:3)	$C_{37}H_{67}O_8P$		
	17	711.43686	711.43679	711.43616	0.98	0.89	$[M+K]^+$	PA(34:2)	$C_{37}H_{69}O_8P$	17	79, 153, 255, 279, 391, 409, 671
	18	697.47829<br>713.45196	697.4780<br>713.45177	697.47788<br>713.45181	0.59<br>0.21	0.17<br>-0.06	$[M+Na]^+$<br>$[M+K]^+$	PA(34:1)	$C_{37}H_{71}O_8P$	17	153, 255, 281, 391, 409, 417, 435, 673
	19	699.47295	-	699.47255	0.57	-	$[M+K]^+$	PA(O-34:1)	$C_{37}H_{73}O_7P$		
	20	701.45132	701.45151	701.45166	-0.48	-0.21	$[M+Na]^+$	PA(P-36:5)	$C_{39}H_{67}O_7P$		
	21	733.42038	733.42063	733.42051	-0.18	0.16	$[M+K]^+$	PA(36:5)	$C_{39}H_{67}O_8P$		
	22	735.43625	-	735.43616	0.12	-	$[M+K]^+$	PA(36:4)	$C_{39}H_{69}O_8P$		
	23	737.45211	737.45231	737.45181	0.41	0.68	$[M+K]^+$	PA(36:3)	$C_{39}H_{71}O_8P$		279, 281, 415, 417, 433, 435
	24	723.49388<br>739.46738	723.49342<br>739.46750	723.49353<br>739.46746	0.48<br>-0.11	-0.15<br>0.05	$[M+Na]^+$<br>$[M+K]^+$	PA(36:2)	$C_{39}H_{73}O_8P$	17	78, 153, 279, 283, 415, 419, 433, 437, 699
	25	741.48304	-	741.48311	-0.09	-	$[M+K]^+$	PA(36:1)	$C_{39}H_{75}O_8P$		79, 153, 281, 283, 417, 419, 435, 437, 701
	26	727.46777	727.46771	727.46731	0.63	0.55	$[M+Na]^+$	PA(P-38:6)	$C_{41}H_{69}O_7P$		
	27	759.43543	-	759.43616	-0.96	-	$[M+K]^+$	PA(38:6)	$C_{41}H_{69}O_8P$		153, 255, 283, 391, 409, 463, 481, 719
	28	761.45158	761.45147	761.45181	-0.30	-0.45	$[M+K]^+$	PA(38:5)	$C_{41}H_{71}O_8P$		
	29	725.51175<br>763.46801	725.51189<br>763.46737	725.51158<br>763.46746	0.23<br>0.72	0.43<br>-0.12	$[M+H]^+$<br>$[M+K]^+$	PA(38:4)	$C_{41}H_{73}O_8P$		153, 259, 283, 303, 419, 437, 439, 457, 723
	30	749.50874<br>765.48304	-<br>765.48387	749.50918<br>765.48311	-0.59<br>-0.09	-<br>0.99	$[M+Na]^+$<br>$[M+K]^+$	PA(38:3)	$C_{41}H_{75}O_8P$		
	31	751.52440<br>767.49919	751.52478<br>767.49893	751.52483<br>767.49876	-0.57<br>0.56	-0.07<br>0.22	$[M+Na]^+$<br>$[M+K]^+$	PA(38:2)	$C_{41}H_{77}O_8P$		
	32	771.53014	771.53026	771.53006	0.10	0.26	$[M+K]^+$	PA(38:0)	$C_{41}H_{81}O_8P$		
	33	785.45107	785.45156	785.45181	-0.94	-0.32	$[M+K]^+$	PA(40:7)	$C_{43}H_{71}O_8P$		
	34	787.46788	-	787.46746	0.53	-	$[M+K]^+$	PA(40:6)	$C_{43}H_{73}O_8P$		153, 283, 327, 419, 437, 463, 481, 747
	35	773.50955<br>789.48282	-<br>789.48298	773.50918<br>789.48311	0.48<br>-0.37	-<br>-0.16	$[M+Na]^+$<br>$[M+K]^+$	PA(40:5)	$C_{43}H_{75}O_8P$		153, 283, 329, 419, 437, 465, 483, 749

Classification	No.	Measured *m/z*		Calculated *m/z*	Error (ppm)		Assignment			Ref.	Structurally specific CID ions (*m/z*)[a)]
		MCAEF	Standard spray		MCAEF	Common spray	Ion form	Compound	Molecular formula		
	36	777.54061	777.54072	777.54048	0.17	0.31	$[M+Na]^+$	PA(40:3)	$C_{43}H_{79}O_8P$		
	37	809.45195	-	809.45181	0.17	-	$[M+K]^+$	PA(42:9)	$C_{45}H_{71}O_8P$		
Phosphoglycerols (PGs)											
	1	547.24304	547.24337	547.24328	-0.44	0.16	$[M+K]^+$	PG(18:2)	$C_{24}H_{45}O_9P$		
	2	573.25867	573.25907	573.25893	-0.45	0.24	$[M+K]^+$	PG(20:3)	$C_{26}H_{47}O_9P$		
	3	559.30057	559.30086	559.30064	-0.13	0.39	$[M+Na]^+$	PG(20:2)	$C_{26}H_{49}O_9P$		
	4	599.27421	599.27468	599.27458	-0.62	0.17	$[M+K]^+$	PG(22:4)	$C_{28}H_{49}O_9P$		
	5	603.30578	603.30597	603.30588	-0.17	0.15	$[M+K]^+$	PG(22:2)	$C_{28}H_{53}O_9P$		
	6	745.47747	-	745.47803	-0.75	-	$[M+K]^+$	PG(P-32:0)	$C_{38}H_{75}O_9P$		
	7	743.48550	-	743.48576	-0.35	-	$[M+H]^+$	PG(34:4)	$C_{40}H_{71}O_{10}P$		
	8	783.45732	783.45743	783.45729	0.04	0.18	$[M+K]^+$	PG(34:3)	$C_{40}H_{73}O_{10}P$		
	9	793.49954	793.49947	793.49901	0.67	0.58	$[M+Na]^+$	PG(36:4)	$C_{42}H_{75}O_{10}P$		
	10	817.53534	817.53567	817.53554	-0.24	0.16	$[M+K]^+$	PG(36:0)	$C_{42}H_{83}O_{10}P$		
	11	801.56403	-	801.56401	0.02	-	$[M+H]^+$	PG(38:3)	$C_{44}H_{81}O_{10}P$		
	12	825.56146	825.56178	825.56161	-0.18	0.21	$[M+Na]^+$	PG(38:2)	$C_{44}H_{83}O_{10}P$		
	13	887.51967	-	887.51989	-0.25	-	$[M+K]^+$	PG(42:7)	$C_{48}H_{81}O_{10}P$		
Phosphatidylserine (PS)											
	1	576.30642	576.30650	576.30621	0.36	0.50	$[M+K]^+$	PS(P-20:0)	$C_{26}H_{52}NO_8P$		
	2	592.30134	592.30146	592.30113	0.36	0.56	$[M+K]^+$	PS(20:0)	$C_{26}H_{52}NO_9P$		
	3	612.26968	612.26999	612.26983	-0.25	0.26	$[M+K]^+$	PS(22:4)	$C_{28}H_{48}NO_9P$		
	4	780.47812	-	780.47861	-0.63	-	$[M+Na]^+$	PS(34:3)	$C_{40}H_{72}NO_{10}P$		
	5	808.50976	-	808.50991	-0.19	-	$[M+Na]^+$	PS(36:3)	$C_{42}H_{76}NO_{10}P$		
	6	828.51537	828.51508	828.51514	0.28	-0.07	$[M+K]^+$	PS(36:1)	$C_{42}H_{80}NO_{10}P$		
	7	824.44713	-	824.44731	-0.22	-	$[M+Na]^+$	PS(38:9)	$C_{44}H_{68}NO_{10}P$		
	8	826.46296	-	826.46296	0.00	-	$[M+Na]^+$	PS(38:8)	$C_{44}H_{70}NO_{10}P$		

Classification	No.	Measured *m/z*		Calculated *m/z*	Error (ppm)		Assignment			Ref.	Structurally specific CID ions (*m/z*)[a)]
		MCAEF	Standard spray		MCAEF	Common spray	Ion form	Compound	Molecular formula		
	9	846.46807	846.46837	846.46819	-0.14	0.21	$[M+K]^+$	PS(38:6)	$C_{44}H_{74}NO_{10}P$		
	10	830.47354	830.47361	830.47328	0.31	0.40	$[M+K]^+$	PS(P-38:6)	$C_{44}H_{74}NO_{9}P$		
	11	834.52516	-	834.52556	-0.48	-	$[M+Na]^+$	PS(38:4)	$C_{44}H_{78}NO_{10}P$		
	12	854.49493	-	854.49426	0.78	-	$[M+Na]^+$	PS(40:8)	$C_{46}H_{74}NO_{10}P$		
	13	856.50985	-	856.50991	-0.07	-	$[M+Na]^+$	PS(40:7)	$C_{46}H_{76}NO_{10}P$		
	14	858.52587	-	858.52556	0.36	-	$[M+Na]^+$	PS(40:6)	$C_{46}H_{78}NO_{10}P$		
	15	860.54139	-	860.54121	0.21	-	$[M+Na]^+$	PS(40:5)	$C_{46}H_{80}NO_{10}P$		
	16	846.62150	846.62196	846.62186	-0.43	0.11	$[M+H]^+$	PS(40:1)	$C_{46}H_{88}NO_{10}P$		
	17	830.62688	-	830.62695	-0.08	-	$[M+H]^+$	PS(P-40:1)	$C_{46}H_{88}NO_{9}P$		
	18	848.63714	848.63754	848.63751	-0.44	0.04	$[M+H]^+$	PS(40:0)	$C_{46}H_{90}NO_{10}P$		
	19	884.54178	-	884.54121	0.64	-	$[M+Na]^+$	PS(42:7)	$C_{48}H_{80}NO_{10}P$		
Phosphatidylinositols (PIs)											
	1	919.47341	-	919.47334	0.08	-	$[M+K]^+$	PI(38:7)	$C_{47}H_{77}O_{13}P$		
	2	925.52053	925.52050	925.52029	0.26	0.23	$[M+K]^+$	PI(38:4)	$C_{47}H_{83}O_{13}P$		240, 259, 283, 303, 419, 437, 439, 457, 581, 599, 601, 619, 886
	3	945.48861	945.48858	945.48899	-0.40	-0.43	$[M+K]^+$	PI(40:8)	$C_{49}H_{79}O_{13}P$		
	4	915.59576	915.59563	915.59571	0.05	-0.09	$[M+H]^+$	PI(40:4)	$C_{49}H_{87}O_{13}P$		
	5	931.53324	-	931.53311	0.14	-	$[M+H]^+$	PI(42:10)	$C_{51}H_{79}O_{13}P$		
	6	975.53674	-	975.53594	0.82	-	$[M+K]^+$	PI(42:7)	$C_{51}H_{85}O_{13}P$		
	7	945.58259	-	945.58274	-0.16	-	$[M+Na]^+$	PI(P-42:6)	$C_{51}H_{87}O_{12}P$		
	8	961.57721	-	961.57765	-0.46	-	$[M+Na]^+$	PI(42:6)	$C_{51}H_{87}O_{13}P$		
Glycerophosphoinositol bisphosphates (PIP2s)											
	1	1035.43662	-	1035.43730	-0.66	-	$[M+K]^+$	PIP2(34:1)	$C_{43}H_{83}O_{19}P_3$		
Glycerophosphoglycero-phosphoglycerols (cardiolipins)											
	1	947.50279	947.50162	947.50212	0.71	-0.53	$[M+Na]^+$	CL(1\'-[18:2(9Z,12Z)/0:0],3\'-	$C_{45}H_{82}O_{15}P_2$		
		963.47618	963.47655	963.47605	0.13	0.52	$[M+K]^+$				

Classification	No.	Measured *m/z*		Calculated *m/z*	Error (ppm)		Assignment			Ref.	Structurally specific CID ions (*m/z*)[a)]
		MCAEF	Standard spray		MCAEF	Common spray	Ion form	Compound	Molecular formula		
								[18:2(9Z,12Z)/0:0])			
Cyclic phosphatidic acids (cPAs)											
	1	415.22193	415.22203	415.22200	-0.17	0.07	$[M+Na]^+$	CPA(16:0)	$C_{19}H_{37}O_6P$		
		431.19611	431.19616	431.19593	0.42	0.53	$[M+K]^+$				
	2	455.19572	455.19588	455.19593	-0.46	-0.11	$[M+K]^+$	CPA(18:2)	$C_{21}H_{37}O_6P$		
	3	441.23769	441.23724	441.23765	0.09	-0.93	$[M+Na]^+$	CPA(18:1)	$C_{21}H_{39}O_6P$		
		457.21177	457.21173	457.21158	0.42	0.33	$[M+K]^+$				
	4	443.25334	443.25320	443.25330	0.09	-0.23	$[M+Na]^+$	CPA(18:0)	$C_{21}H_{41}O_6P$		
		459.22743	459.22741	459.22723	0.44	0.39	$[M+K]^+$				
CDP-Glycerols											
	1	980.53779	-	980.53722	0.58	-	$[M+H]^+$	CDP-DG(34:1)	$C_{46}H_{83}N_3O_{15}P_2$		
		1018.49325	-	1018.49310	0.15	-	$[M+K]^+$				
	2	982.55256	-	982.55287	-0.32	-	$[M+H]^+$	CDP-DG(34:0)	$C_{46}H_{85}N_3O_{15}P_2$		
		1020.50867	-	1020.50875	-0.08	-	$[M+K]^+$				
	3	1010.58474	-	1010.58417	0.54	-	$[M+H]^+$	CDP-DG(36:0)	$C_{46}H_{89}N_3O_{15}P_2$		
	4	1058.58469	-	1058.58417	0.49	-	$[M+H]^+$	CDP-DG(40:4)	$C_{52}H_{89}N_3O_{15}P_2$		
		1096.54020	-	1096.54005	0.14	-	$[M+K]^+$				
Glycerophosphate											
	1	467.25331	-	467.25330	0.02	-	$[M+Na]^+$	sn-3-O-(geranylgeranyl)glycerol 1-phosphate	$C_{23}H_{41}O_6P$		
		483.22728	-	483.22723	0.10	-	$[M+K]^+$				
**Sphingolipids**											
Ceramides (Cers)											
	1	464.35032	464.35027	464.35005	0.58	0.47	$[M+K]^+$	C-8 Ceramide	$C_{26}H_{51}NO_3$		
	2	602.49131	602.49122	602.49090	0.68	0.53	$[M+K]^+$	Cer(d36:2)	$C_{36}H_{69}NO_3$		
	3	604.50685	604.50681	604.50655	0.50	0.43	$[M+K]^+$	Cer(d36:1)	$C_{36}H_{71}NO_3$		
	4	684.47275	-	684.47288	-0.19	-	$[M+K]^+$	CerP(d36:1)	$C_{36}H_{72}NO_6P$		

Classification	No.	Measured *m/z*		Calculated *m/z*	Error (ppm)		Assignment			Ref.	Structurally specific CID ions (*m/z*)[a)]
		MCAEF	Standard spray		MCAEF	Common spray	Ion form	Compound	Molecular formula		
	5	632.53811	632.53823	632.53785	0.41	0.60	$[M+K]^+$	Cer(d38:1)	$C_{38}H_{75}NO_3$		
	6	686.58456	686.58460	686.58480	-0.35	-0.29	$[M+K]^+$	Cer(d42:2)	$C_{42}H_{81}NO_3$		
	7	766.55160	-	766.55113	0.61	-	$[M+K]^+$	CerP(d42:2)	$C_{42}H_{82}NO_6P$	17	264, 749, 767
	8	688.60044	-	688.60045	-0.02	-	$[M+K]^+$	Cer(d42:1)	$C_{42}H_{83}NO_3$		
Sphingomyelins (SMs)											
	1	703.57475	-	703.57485	-0.14	-	$[M+H]^+$	SM(d34:1)	$C_{39}H_{79}N_2O_6P$	17, 23, 28	163, 184, 682
		725.55673	725.55694	725.55680	-0.10	0.19	$[M+Na]^+$			17, 19, 25, 36	
	2	753.58804	753.58822	753.58810	-0.08	-0.16	$[M+Na]^+$	SM(d36:1)	$C_{41}H_{83}N_2O_6P$	26	86, 184, 703, 731
		769.56224	769.56187	769.56203	0.27	-0.21	$[M+K]^+$			17, 26, 37	
	3	797.59361	797.59355	797.59333	0.35	0.28	$[M+K]^+$	SM(d38:1)	$C_{43}H_{87}N_2O_6P$	17, 32	614, 738
	4	787.66858	-	787.66875	-0.22	-	$[M+H]^+$	SM(d40:1)	$C_{45}H_{91}N_2O_6P$	23	
		825.62452	825.62481	825.62463	-0.13	0.22	$[M+K]^+$				
	5	813.68484	-	813.68440	0.54	-	$[M+H]^+$	SM(d42:2)	$C_{47}H_{93}N_2O_6P$	23	652, 776
		851.64041	851.64021	851.64028	0.15	-0.08	$[M+K]^+$				
	6	815.70041	-	815.70005	0.44	-	$[M+H]^+$	SM(d42:1)	$C_{47}H_{95}N_2O_6P$	23	654, 778
		837.68232	837.68204	837.68200	0.38	0.05	$[M+Na]^+$				
		853.65645	853.65568	853.65593	0.61	-0.29	$[M+K]^+$			17	
Glycosphingolipids											
	1	500.29867	500.29815	500.29841	0.52	-0.52	$[M+K]^+$	Glucosyl sphingosine	$C_{24}H_{47}NO_7$		
	2	828.54447	-	828.54436	0.13	-	$[M+Na]^+$	LacCer(d30:1)	$C_{42}H_{79}NO_{13}$		264, 447, 465, 627, 789, 807
	3	766.55942	766.55930	766.55938	0.05	-0.10	$[M+K]^+$	GlcCer(d36:1)	$C_{42}H_{81}NO_8$	22	
	4	856.57577	-	856.57566	0.13	-	$[M+Na]^+$	LacCer(d32:1)	$C_{44}H_{83}NO_{13}$		
	5	852.58713	-	852.58652	0.72	-	$[M+H]^+$	(3'-sulfo)Galβ-Cer(d38:0(2OH))	$C_{44}H_{85}NO_{12}S$		
	6	794.59095	794.59084	794.59068	0.34	0.20	$[M+K]^+$	GalCer(d38:1)	$C_{44}H_{85}NO_8$	22	
	7	820.60674	820.60671	820.60633	0.50	0.46	$[M+K]^+$	GlcCer(d40:2)	$C_{46}H_{87}NO_8$	22	

Classification	No.	Measured *m/z*		Calculated *m/z*	Error (ppm)		Assignment			Ref.	Structurally specific CID ions (*m/z*)[a]
		MCAEF	Standard spray		MCAEF	Common spray	Ion form	Compound	Molecular formula		
	8	836.60133	-	836.60124	0.11	-	$[M+K]^+$	GlcCer(d16:2/24:0(2OH))	$C_{46}H_{87}NO_9$		
	9	822.62190	822.62156	822.62198	-0.10	-0.51	$[M+K]^+$	GlcCer(d40:1)	$C_{46}H_{89}NO_8$	22	
	10	928.61212	-	928.61220	-0.09	-	$[M+K]^+$	LacCer(d36:1)	$C_{48}H_{91}NO_{13}$		
	11	832.66350	832.66332	832.66369	-0.23	-0.44	$[M+Na]^+$	GlcCer(d42:2)	$C_{48}H_{91}NO_8$	22	
		848.63831	848.63842	848.63763	0.80	0.93	$[M+K]^+$				
	12	892.67158	-	892.67197	-0.44	-	$[M+H]^+$	LacCer(d36:0)	$C_{48}H_{93}NO_{13}$		
	13	850.65367	850.65337	850.65328	0.46	0.11	$[M+K]^+$	GlcCer(d42:1)	$C_{48}H_{93}NO_8$	22	
	14	852.66911	-	852.66893	0.21	-	$[M+K]^+$	GlcCer(d42:0)	$C_{48}H_{95}NO_8$		
	15	876.66849	876.66867	876.66893	-0.50	-0.30	$[M+K]^+$	GlcCer(d44:2)	$C_{50}H_{95}NO_8$	22	
	16	878.68466	878.68478	878.68458	0.09	0.23	$[M+K]^+$	GlcCer(d44:1)	$C_{50}H_{97}NO_8$		
	17	1010.69083	-	1010.69045	0.38	-	$[M+K]^+$	Galβ1-4Glcβ-Cer(d42:2)	$C_{54}H_{101}NO_{13}$		
	18	1012.70616	-	1012.70610	0.06	-	$[M+K]^+$	Galβ1-4Glcβ-Cer(d42:1)	$C_{54}H_{103}NO_{13}$		
Sphingoid bases											
	1	264.19316	-	264.19340	-0.91	-	$[M+Na]^+$	(4E,6E,d14:2) sphingosine	$C_{14}H_{27}NO_2$		
Ceramide phosphoinositols (PI-Cers)											
	1	852.50034	-	852.49989	0.53	-	$[M+K]^+$	PI-Cer(t34:0(2OH))	$C_{40}H_{80}NO_{13}P$		
	2	838.61683	-	838.61678	0.06	-	$[M+H]^+$	PI-Cer(d38:0)	$C_{44}H_{88}NO_{11}P$		
	3	864.63279	-	864.63243	0.42	-	$[M+H]^+$	PI-Cer(d40:10)	$C_{46}H_{90}NO_{11}P$		
	4	866.64805	-	866.64808	-0.03	-	$[M+H]^+$	PI-Cer(d40:0)	$C_{46}H_{92}NO_{11}P$		
	5	904.62434	-	904.62494	-0.66	-	$[M+Na]^+$	PI-Cer(t40:0)	$C_{46}H_{92}NO_{12}P$		
	6	894.67917	-	894.67938	-0.23	-	$[M+H]^+$	PI-Cer(d42:0)	$C_{48}H_{96}NO_{11}P$		
	7	1154.70941	-	1154.70921	0.17	-	$[M+K]^+$	MIPC(t44:0(2OH))	$C_{56}H_{110}NO_{18}P$		
**Neutral Lipids**											
**Glycerolipids**											
Monoacylglycerols (MAGs)											
	1	369.24037	369.24012	369.24017	0.54	-0.14	$[M+K]^+$	MG (16:0)	$C_{19}H_{38}O_4$		239, 257, 313, 331, 369
	2	379.28181	379.28191	379.28188	-0.18	0.08	$[M+Na]^+$	MG (18:1)	$C_{21}H_{40}O_4$		
		395.25575	395.25583	395.25582	-0.18	0.03	$[M+K]^+$				

Classification	No.	Measured *m/z*		Calculated *m/z*	Error (ppm)		Assignment			Ref.	Structurally specific CID ions (*m/z*)[a)]
		MCAEF	Standard spray		MCAEF	Common spray	Ion form	Compound	Molecular formula		
	3	397.27164	-	397.27147	0.43	-	$[M+K]^+$	MG (18:0)	$C_{21}H_{42}O_4$		
	4	417.24037	417.24025	417.24017	0.48	0.19	$[M+K]^+$	MG (20:4)	$C_{23}H_{38}O_4$		
	5	419.25581	419.25577	419.25582	-0.02	-0.12	$[M+K]^+$	MG (20:3)	$C_{23}H_{40}O_4$		
	6	425.26612	-	425.26623	-0.26	-	$[M+Na]^+$	MG (22:6)	$C_{25}H_{38}O_4$		
	7	445.27173	445.27173	445.27147	0.58	0.58	$[M+K]^+$	MG (22:4)	$C_{25}H_{42}O_4$		
Diacylglycerols (DAGs)											
		551.50365	551.50347	551.50339	0.47	0.15	$[M+H]^+$				
	1	573.48551	-	573.48533	0.31	-	$[M+Na]^+$	DG(P-32:1)	$C_{35}H_{66}O_4$		
		589.45915	-	589.45927	-0.20	-	$[M+K]^+$				
	2	607.47032	607.47016	607.46983	0.81	0.54	$[M+K]^+$	DG(32:0)	$C_{35}H_{68}O_5$		313, 551, 569
	3	561.52376	561.52389	561.52412	-0.64	-0.41	$[M+H]^+$	1-tetradecanyl-2-(8-[3]-ladderane-octanyl)-sn-glycerol	$C_{37}H_{68}O_3$		
	4	631.47028	-	631.46983	0.71	-	$[M+K]^+$	DG(34:2)	$C_{37}H_{68}O_5$		
	5	633.48581	633.48582	633.48548	0.52	0.54	$[M+K]^+$	DG(34:1)	$C_{37}H_{70}O_5$		
	6	619.50655	619.50647	619.50622	0.53	0.40	$[M+K]^+$	DG(O-34:1)	$C_{37}H_{72}O_4$		
	7	635.50160	-	635.50113	0.74	-	$[M+K]^+$	DG(34:0)	$C_{37}H_{72}O_5$		229, 250, 301,341, 597
	8	655.47014	655.46930	655.46983	0.47	-0.81	$[M+K]^+$	DG(36:4)	$C_{39}H_{68}O_5$		
	9	603.53505	603.53483	603.53469	0.60	0.23	$[M+H]^+$	1-(14-methyl-pentadecanoyl)-2-(8-[3]-ladderane-octanyl)-sn-glycerol	$C_{39}H_{70}O_4$		
		641.49026	641.49016	641.49057	-0.48	-0.64	$[M+K]^+$				
	10	657.48501	-	657.48548	-0.71	-	$[M+K]^+$	DG(36:3)	$C_{39}H_{70}O_5$		
	11	589.55554	589.55568	589.55542	0.20	0.44	$[M+H]^+$	1-hexadecanyl-2-(8-[3]-ladderane-octanyl)-sn-glycerol	$C_{39}H_{72}O_3$		
		611.53758	-	611.53737	0.34	-	$[M+Na]^+$				
	12	659.50127	659.50094	659.50113	0.21	-0.29	$[M+K]^+$	DG(36:2)	$C_{39}H_{72}O_5$		
	13	661.51722	661.51710	661.51678	0.67	0.48	$[M+K]^+$	DG(36:1)	$C_{39}H_{72}O_5$		
	14	621.48715	-	621.48774	-0.95	-	$[M+H]^+$	1-(6-[5]-ladderane-hexanoyl)-2-(8-[3]-ladderane-octanyl)-sn-	$C_{41}H_{64}O_4$		

Classification	No.	Measured *m/z*		Calculated *m/z*	Error (ppm)		Assignment			Ref.	Structurally specific CID ions (*m/z*)[a)]
		MCAEF	Standard spray		MCAEF	Common spray	Ion form	Compound	Molecular formula		
								glycerol			
	15	679.47020	679.46969	679.46983	0.54	-0.21	$[M+K]^+$	DG(38:6)	$C_{41}H_{68}O_5$		
	16	681.48559	681.48600	681.48548	0.16	0.76	$[M+K]^+$	DG(38:5)	$C_{41}H_{70}O_5$		
	17	683.50180	683.50168	683.50113	0.98	0.80	$[M+K]^+$	DG(38:4)	$C_{41}H_{72}O_5$		
	18	687.53232	687.53220	687.53243	-0.16	-0.33	$[M+K]^+$	DG(38:2)	$C_{41}H_{76}O_5$		
	19	689.54838	689.54863	689.54808	0.44	0.80	$[M+K]^+$	DG(38:1)	$C_{41}H_{78}O_5$		
	20	682.45663	682.45673	682.45677	-0.21	-0.06	$[M+Na]^+$	DG(40:8)	$C_{43}H_{63}D_5O_5$		250, 287, 301, 325, 660
	21	699.43846	-	699.43853	-0.10	-	$[M+K]^+$	DG(40:10)	$C_{43}H_{64}O_5$		
	22	649.51967	649.51920	649.51904	0.97	0.25	$[M+H]^+$	1-(8-[5]-ladderane-octanoyl)-2-(8-[3]-ladderane-octanyl)-sn-glycerol	$C_{43}H_{68}O_4$		
	23	635.53977	-	635.53977	0.00	-	$[M+H]^+$	1-(8-[5]-ladderane-octanyl)-2-(8-[3]-ladderane-octanyl)-sn-glycerol	$C_{43}H_{70}O_3$		
	24	651.53511	651.53446	651.53469	0.64	-0.35	$[M+H]^+$	1-(8-[3]-ladderane-octanoyl-2-(8-[3]-ladderane-octanyl)-sn-glycerol	$C_{43}H_{70}O_4$		
	25	707.50059	707.50137	707.50113	-0.76	0.34	$[M+K]^+$	DG(40:6)	$C_{43}H_{72}O_5$		
	26	725.45413	725.45443	725.45418	-0.07	0.34	$[M+K]^+$	DG(42:11)	$C_{45}H_{66}O_5$		
Triradylglycerols (TAGs)											
	1	869.66542	-	869.66537	0.06	-	$[M+H]^+$	TG(54:11)	$C_{57}H_{88}O_6$		
	2	873.69664	-	873.69667	-0.03	-	$[M+H]^+$	TG(54:9)	$C_{57}H_{92}O_6$		
	3	995.70995	-	995.70991	0.04	-	$[M+Na]^+$	TG(62:15)	$C_{65}H_{96}O_6$		
	4	997.72583	-	997.72556	-0.14	-	$[M+Na]^+$	TG(62:14)	$C_{65}H_{98}O_6$		
	5	1035.68350	-	1035.68385	-0.34	-	$[M+K]^+$	TG(64:17)	$C_{67}H_{96}O_6$		
Other Glycerolipids											
	1	834.62108	834.62159	834.62183	-0.90	-0.29	$[M+Na]^+$	1-(9Z,1Z-octadecadienoyl)-2-(10Z,13Z,16Z,19Z-docosatetraenoyl)-3-O-[hydroxymethyl-N,N,N-trimethyl-beta-alanine]-	$C_{50}H_{85}NO_7$		

Classification	No.	Measured *m/z*		Calculated *m/z*	Error (ppm)		Assignment			Ref.	Structurally specific CID ions (*m/z*)[a)]
		MCAEF	Standard spray		MCAEF	Common spray	Ion form	Compound	Molecular formula		
								glycerol			
**Sterol Lipids**											
	1	429.24054	429.24023	429.24017	0.86	0.14	$[M+K]^+$	C24 bile acids and/or its isomers	$C_{24}H_{38}O_4$		
	2	457.27128	457.27125	457.27147	-0.42	-0.48	$[M+K]^+$	24-northornasterol A	$C_{26}H_{42}O_4$		
	3	423.30220	-	423.30237	-0.40	-	$[M+K]^+$	Dehydrocholesterol	$C_{27}H_{44}O$		
	4	471.28682	-	471.28712	-0.64	-	$[M+K]^+$	C27 bile acids and/or its isomers	$C_{27}H_{44}O_4$		
	5	409.34413<br>425.31823	409.34418<br>425.31836	409.34409<br>425.31802	0.10<br>0.49	0.22<br>0.80	$[M+Na]^+$<br>$[M+K]^+$	Cholesterol	$C_{27}H_{46}O$		
	6	473.32356	473.32393	473.32375	-0.40	0.38	$[M+Na]^+$	C27 bile acids and/ or its isomers	$C_{27}H_{46}O_5$		
	7	489.31869	-	489.31866	0.06	-	$[M+Na]^+$	C27 bile acids and/ or its isomers	$C_{27}H_{46}O_6$		
	8	485.30288	485.30306	485.30277	0.23	0.58	$[M+K]^+$	Ergosterols and C24-methyl derivatives	$C_{28}H_{46}O_4$		
	9	431.32854	-	431.32844	0.23	-	$[M+Na]^+$	Conicasterol B	$C_{29}H_{44}O$		
	10	497.33943	497.33956	497.33915	0.56	0.82	$[M+K]^+$	C30 isoprenoids	$C_{30}H_{50}O_3$		
	11	777.41861	-	777.41859	0.03	-	$[M+K]^+$	Spirostanols and/ or its isomers	$C_{40}H_{66}O_{12}$		
	12	827.41889	-	827.41898	-0.11	-	$[M+K]^+$	Spirostanols and/ or its isomers	$C_{40}H_{68}O_{15}$		
**Prenol Lipids**											
	1	445.29235	445.29251	445.29245	-0.22	0.13	$[M+Na]^+$	19-(3-methyl-butanoyloxy)-villanovane-13alpha,17-diol	$C_{25}H_{42}O_5$		
**Fatty acyls**											
Fatty acids (FAs)											
	1	319.20346	-	319.20339	0.22	-	$[M+K]^+$	FA(18:2)	$C_{18}H_{32}O_2$	[2]	
	2	321.21911	321.21914	321.21904	0.22	0.31	$[M+K]^+$	FA(18:1)	$C_{18}H_{34}O_2$	[2]	
	3	343.20348	343.20408	343.20339	0.26	-0.90	$[M+K]^+$	FA(20:4)	$C_{20}H_{32}O_2$	[2]	59, 80, 177, 205, 259, 303

Classification	No.	Measured *m/z*		Calculated *m/z*	Error (ppm)		Assignment			Ref.	Structurally specific CID ions (*m/z*)[a)]
		MCAEF	Standard spray		MCAEF	Common spray	Ion form	Compound	Molecular formula		
	4	367.20345	367.20339	367.20339	0.16	0.00	$[M+K]^+$	FA(22:6)	$C_{22}H_{32}O_2$	2	
	5	393.29789	-	393.29753	0.92	-	$[M+Na]^+$	FA(22:0)	$C_{22}H_{42}O_4$	2	
		409.27128	409.27132	409.27147	-0.46	-0.37	$[M+K]^+$			2	
	6	465.33428	465.33448	465.33407	0.45	0.88	$[M+K]^+$	FA(26:0)	$C_{26}H_{50}O_4$	2	
**Number of Lipids**						**MCAEF: 320 *vs.* common spray: 208**					
**Other compounds**											
	1	322.05478	322.05479	322.05483	-0.16	-0.12	$[M+K]^+$	Guanosine	$C_{10}H_{13}N_5O_5$		
	2	327.03528	-	327.03526	0.06	-	$[M+Na]^+$	Thymidine 3,5-cyclic monophosphate	$C_{10}H_{13}N_2O_7P$		
	3	352.04158	352.04164	352.04174	-0.45	-0.28	$[M+Na]^+$	Cyclic adenosine monophosphate (cAMP)	$C_{10}H_{12}N_5O_6P$		
		368.01550	368.01546	368.01568	-0.49	-0.60	$[M+K]^+$				
	4	1146.50914	-	1146.50865	0.43	-	$[M+H]^+$	CoA(26:0)	$C_{47}H_{86}N_7O_{17}P_3S$		
		1168.49083	-	1168.49060	0.20	-	$[M+Na]^+$				
**Number of Lipids**								**MCAEF: 4 *vs.* common spray: 2**			

a), Structurally specific CID ions of extracted lipids were detected by LC-MS/MS using CID. Red fragment ions were detected in the positive ion mode, and blue fragment ions were detected in the negative ion mode.

Supplementary Information Table S3. Comparison of lipid detection on rat brain sections by MALDI-FTICR MS in the negative-ion mode using MCAEF and common spray methods for quercetin matrix deposition, respectively.

Classification	No.	Measured *m/z*		Calculated *m/z*	Error (ppm)		Assignment			Ref.	Structurally specific CID ions (*m/z*)[a)]
		MCAEF	Common spray		MCAEF	Common spray	Ion form	Compound	Molecular formula		
**Glycerophospholipids**											
Phosphatidylcholines (PCs)											
	1	554.30174	554.30186	554.30189	-0.27	-0.05	$[M+Cl]^-$	PC(18:2)	C26H50NO7P		
	2	558.33304	558.33334	558.33319	-0.27	0.27	$[M+Cl]^-$	PC(18:0)	C26H54NO7P		104, 184, 506, 524 or 224, 283, 508, 558
	3	764.50034	764.50009	764.50026	0.10	-0.22	$[M+Cl]^-$	PC(32:2)	C40H76NO8P		
	4	816.53154	-	816.53156	-0.02	-	$[M+Cl]^-$	PC(36:4)	C44H80NO8P		184, 783
	5	804.53081	-	804.53150	-0.86	-	$[M+K-2H]^-$	PC(O-36:4)	$C_{44}H_{82}NO_7P$		
	6	844.56278	844.56257	844.56286	-0.09	-0.34	$[M+Cl]^-$	PC(38:4)	C46H84NO8P		184, 627, 752, 811 or 259, 283, 303, 490, 508, 510, 528, 794, 844
	7	860.59426	-	860.59410	0.19	-	$[M+K-2H]^-$	PC(O-40:4)	$C_{48}H_{90}NO_7P$		
	8	874.60931	874.60956	874.60981	-0.57	-0.29	$[M+Cl]^-$	PC(40:3)	$C_{48}H_{90}NO_8P$		
		876.58946	876.58924	876.58901	0.51	0.26	$[M+K-2H]^-$				
	9	876.62535	876.62587	876.62546	-0.13	0.47	$[M+Cl]^-$	PC(40:2)	$C_{48}H_{92}NO_8P$		
	10	880.62069	-	880.62031	0.43	-	$[M+K-2H]^-$	PC(40:1)	$C_{48}H_{94}NO_8P$		
	11	896.59408	896.59424	896.59416	-0.09	0.09	$[M+Cl]^-$	PC(42:6)	C50H88NO8P		
	12	902.60392	-	902.60466	-0.82	-	$[M+K-2H]^-$	PC(42:4)	$C_{50}H_{92}NO_8P$		
	13	934.66756	934.66751	934.66726	0.32	0.27	$[M+K-2H]^-$	PC(44:2)	$C_{52}H_{100}NO_8P$		
	14	930.63646	-	930.63596	0.54	-	$[M+K-2H]^-$	PC(44:4)	$C_{52}H_{96}NO_8P$		
	15	932.65184	932.65145	932.65161	0.25	-0.17	$[M+K-2H]^-$	PC(44:3)	$C_{52}H_{98}NO_8P$		

Classification	No.	Measured *m/z*		Calculated *m/z*	Error (ppm)		Assignment			Ref.	Structurally specific CID ions (*m/z*)[a)]
		MCAEF	Common spray		MCAEF	Common spray	Ion form	Compound	Molecular formula		
Phosphatidylethanolamines (PEs)											
	1	452.27854	-	452.27826	0.62	-	$[M-H]^-$	PE(16:0)	$C_{21}H_{44}NO_7P$		153, 196, 214, 255, 378, 409, 452
	2	478.29393	478.29394	478.29391	0.04	0.06	$[M-H]^-$	PE(18:1)	$C_{23}H_{46}NO_7P$		153, 196, 214, 281, 417,435, 478
	3	480.30981	-	480.30956	0.52	-	$[M-H]^-$	PE(18:0)	$C_{23}H_{48}NO_7P$		140, 153, 196, 214, 283, 419, 437, 480
	4	500.27854	-	500.27826	0.56	-	$[M-H]^-$	PE(20:4)	$C_{25}H_{44}NO_7P$		153, 195, 259, 303, 500
	5	502.29341	-	502.29391	-0.99	-	$[M-H]^-$	PE(20:3)	$C_{25}H_{46}NO_7P$		
	6	506.32500	506.32492	506.32521	-0.41	-0.57	$[M-H]^-$	PE(20:1)	$C_{25}H_{50}NO_7P$		
	7	508.34087	-	508.34086	0.02	-	$[M-H]^-$	PE(20:0)	$C_{25}H_{52}NO_7P$		
	8	524.27848	-	524.27826	0.42	-	$[M-H]^-$	PE(22:6)	$C_{27}H_{44}NO_7P$		
	9	526.29340	526.29427	526.29391	-0.97	0.68	$[M-H]^-$	PE(22:5)	$C_{27}H_{46}NO_7P$		
	10	528.30963	528.30939	528.30956	0.13	-0.32	$[M-H]^-$	PE(22:4)	$C_{27}H_{48}NO_7P$		
	11	552.30959	-	552.30956	0.05	-	$[M-H]^-$	LysoPE(24:6)	$C_{29}H_{48}NO_7P$		
	12	712.49217	-	712.49228	-0.15	-	$[M-H]^-$	PE(34:3)	$C_{39}H_{72}NO_8P$		
	13	714.50768	-	714.50793	-0.35	-	$[M-H]^-$	PE(34:2)	$C_{39}H_{74}NO_8P$		
	14	716.52392	716.52340	716.52358	0.47	-0.25	$[M-H]^-$	PE(34:1)	$C_{39}H_{76}NO_8P$	21, 38	153, 196, 255, 281, 434, 452, 460, 478, 716
	15	718.53928	718.53959	718.53923	0.07	0.50	$[M-H]^-$	PE(34:0)	$C_{39}H_{78}NO_8P$		
	16	722.51337	722.51293	722.51301	0.50	-0.11	$[M-H]^-$	PE(P-36:4)	$C_{41}H_{74}NO_7P$	38	
	17	738.50744	738.50770	738.50793	-0.66	-0.31	$[M-H]^-$	PE(36:4)	C41H74NO8P		
	18	726.54447	726.54404	726.54431	0.22	-0.37	$[M-H]^-$	PE(P-36:2)	$C_{41}H_{78}NO_7P$	21, 38	
	19	742.53937	742.53940	742.53923	0.19	0.23	$[M-H]^-$	PE(36:2)	$C_{41}H_{78}NO_8P$	38	196, 281, 460, 478, 742
	20	728.56049	728.56040	728.55996	0.73	0.60	$[M-H]^-$	PE(P-36:1)	$C_{41}H_{80}NO_7P$	21, 38	79, 140, 255, 309, 281,728
	21	744.55476	744.55515	744.55488	-0.16	0.36	$[M-H]^-$	PE(36:1)	$C_{41}H_{80}NO_8P$	21, 38	

Classification	No.	Measured *m/z*		Calculated *m/z*	Error (ppm)		Assignment			Ref.	Structurally specific CID ions (*m/z*)[a)]
		MCAEF	Common spray		MCAEF	Common spray	Ion form	Compound	Molecular formula		
	22	746.57089	-	746.57053	0.48	-	$[M-H]^-$	PE(36:0)	$C_{41}H_{82}NO_8P$		153, 283, 419, 437, 462, 480, 746
	23	762.50754	762.50791	762.50793	-0.51	-0.03	$[M-H]^-$	PE(38:6)	$C_{43}H_{74}NO_8P$	38	
	24	764.52405	764.52378	764.52358	0.61	0.26	$[M-H]^-$	PE(38:5)	$C_{43}H_{76}NO_8P$	38	
	25	750.54448	750.54468	750.54431	0.23	0.49	$[M-H]^-$	PE(P-38:4)	$C_{43}H_{78}NO_7P$	38	
	26	766.53930	766.53950	766.53923	0.09	0.35	$[M-H]^-$	PE(38:4)	$C_{43}H_{78}NO_8P$	38	259, 283, 303, 462, 480, 482, 500, 767
	27	782.53464	-	782.53414	0.64	-	$[M-H]^-$	PE(38:4(12OH))	$C_{43}H_{78}NO_9P$		
	28	754.57596	754.57550	754.57561	0.46	-0.15	$[M-H]^-$	PE(P-38:2)	$C_{43}H_{82}NO_7P$		
	29	770.57050	-	770.57053	-0.04	-	$[M-H]^-$	PE(38:2)	$C_{43}H_{82}NO_8P$	38	
	30	756.59121	-	756.59126	-0.07	-	$[M-H]^-$	PE(P-38:1)	$C_{43}H_{84}NO_7P$		
	31	772.58602	772.58630	772.58618	-0.21	0.16	$[M-H]^-$	PE(38:1)	$C_{43}H_{84}NO_8P$		
	32	772.52884	772.52797	772.52866	0.23	-0.89	$[M-H]^-$	PE(P-40:7)	$C_{45}H_{76}NO_7P$		
	33	788.52324	788.52389	788.52358	-0.43	0.39	$[M-H]^-$	PE(40:7)	$C_{45}H_{76}NO_8P$		
	34	774.54441	774.54424	774.54431	0.13	-0.09	$[M-H]^-$	PE(P-40:6)	$C_{45}H_{78}NO_7P$	38	283, 327, 446, 464, 774
	35	790.53881	790.53852	790.53923	-0.53	-0.90	$[M-H]^-$	PE(40:6)	$C_{45}H_{78}NO_8P$	32, 38	283, 327, 462, 480, 506, 524, 790
	36	776.55967	776.55965	776.55996	-0.37	-0.40	$[M-H]^-$	PE(P-40:5)	$C_{45}H_{80}NO_7P$		
	37	792.55562	792.55505	792.55488	0.93	0.21	$[M-H]^-$	PE(40:5)	$C_{45}H_{80}NO_8P$		
	38	778.57537	778.57567	778.57561	-0.31	0.08	$[M-H]^-$	PE(P-40:4)	$C_{45}H_{82}NO_7P$		
	39	794.57124	794.57051	794.57053	0.89	-0.03	$[M-H]^-$	PE(40:4)	$C_{45}H_{82}NO_8P$	32	
	40	796.58632	796.58643	796.58618	0.18	0.31	$[M-H]^-$	PE(40:3)	$C_{45}H_{84}NO_8P$		
	41	798.60174	798.60153	798.60183	-0.11	-0.38	$[M-H]^-$	PE(40:2)	$C_{45}H_{86}NO_8P$		
	42	800.61736	800.61745	800.61748	-0.15	-0.04	$[M-H]^-$	PE(40:1)	$C_{45}H_{88}NO_8P$		
	43	812.52374	-	812.52358	0.20	-	$[M-H]^-$	PE(42:9)	$C_{47}H_{76}NO_8P$		
	44	816.55471	816.55442	816.55488	-0.21	-0.56	$[M-H]^-$	PE(42:7)	$C_{47}H_{80}NO_8P$		
	45	818.57026	-	818.57053	-0.33	-	$[M-H]^-$	PE(42:6)	$C_{47}H_{82}NO_8P$		

Classification	No.	Measured *m/z*		Calculated *m/z*	Error (ppm)		Assignment			Ref.	Structurally specific CID ions (*m/z*)[a)]
		MCAEF	Common spray		MCAEF	Common spray	Ion form	Compound	Molecular formula		
	46	820.58636	-	820.58618	0.22	-	$[M-H]^-$	PE(42:5)	$C_{47}H_{84}NO_8P$		
	47	822.60197	-	822.60183	0.17	-	$[M-H]^-$	PE(42:4)	$C_{47}H_{86}NO_8P$		
	48	838.53954	838.53921	838.53923	0.37	-0.02	$[M-H]^-$	PE(44:10)	$C_{49}H_{78}NO_8P$		
		874.51537	874.51566	874.51591	-0.62	-0.29	$[M+Cl]^-$				
	49	840.55467	-	840.55488	-0.25	-	$[M-H]^-$	PE(44:9)	$C_{49}H_{80}NO_8P$		
	50	850.63324	850.63334	850.63313	0.13	0.25	$[M-H]^-$	PE(44:4)	$C_{49}H_{90}NO_8P$		
		886.61021	-	886.60981	0.45	-	$[M+Cl]^-$				
	51	914.64111	-	914.64111	0.00	-	$[M+Cl]^-$	PE(46:4)	$C_{51}H_{94}NO_8P$		
	52	880.68014	-	880.68008	0.07	-	$[M-H]^-$	PE(46:3)	$C_{51}H_{96}NO_8P$		
		916.65626	916.65606	916.65676	-0.55	-0.76	$[M+Cl]^-$				
	53	918.67254	-	918.67241	0.14	-	$[M+Cl]^-$	PE(46:2)	$C_{51}H_{98}NO_8P$		
		920.65164	-	920.65161	0.03	-	$[M+K-2H]^-$				
Phosphatidic acids (PAs)											
	1	427.18936	-	427.18911	0.59	-	$[M-H]^-$	PA(18:5)	$C_{21}H_{33}O_7P$		
	2	429.20491	-	429.20476	0.35	-	$[M-H]^-$	PA(18:4)	$C_{21}H_{35}O_7P$		
	3	431.22021	-	431.22041	-0.46	-	$[M-H]^-$	PA(18:3)	$C_{21}H_{37}O_7P$		
	4	433.23603	-	433.23606	-0.07	-	$[M-H]^-$	PA(18:2)	$C_{21}H_{39}O_7P$		
	5	435.25159	435.25140	435.25171	-0.28	-0.71	$[M-H]^-$	PA(18:1)	$C_{21}H_{41}O_7P$		79, 153, 171, 281, 435
	6	437.26773	437.26778	437.26736	0.85	0.96	$[M-H]^-$	PA(18:0)	$C_{21}H_{43}O_7P$		79, 153, 171, 283, 437
	7	457.23622	-	457.23606	0.35	-	$[M-H]^-$	PA(20:4)	$C_{23}H_{39}O_7P$		153, 171, 259, 303, 457
	8	459.25173	459.25148	459.25171	0.04	-0.50	$[M-H]^-$	PA(20:3)	$C_{23}H_{41}O_7P$		
	9	461.26718	461.26712	461.26736	-0.39	-0.52	$[M-H]^-$	PA(20:2)	$C_{23}H_{43}O_7P$		
	10	463.28313	-	463.28301	0.26	-	$[M-H]^-$	PA(20:1)	$C_{23}H_{45}O_7P$		
	11	449.30360	-	449.30375	-0.33	-	$[M-H]^-$	PA(P-20:0)	$C_{23}H_{47}O_6P$		
	12	465.29838	465.29833	465.29866	-0.60	-0.71	$[M-H]^-$	PA(20:0)	$C_{23}H_{47}O_7P$		
	13	481.23637	-	481.23606	0.64	-	$[M-H]^-$	PA(22:6)	$C_{25}H_{39}O_7P$		

Classification	No.	Measured *m/z*		Calculated *m/z*	Error (ppm)		Assignment			Ref.	Structurally specific CID ions (*m/z*)[a)]
		MCAEF	Common spray		MCAEF	Common spray	Ion form	Compound	Molecular formula		
	14	489.29827	-	489.29866	-0.80	-	$[M-H]^-$	PA(22:2)	$C_{25}H_{47}O_7P$		
	15	647.46559	647.46596	647.46573	-0.22	0.36	$[M-H]^-$	PA(32:0)	$C_{35}H_{69}O_8P$		79, 153, 255, 391, 409, 647
	16	671.46589	-	671.46573	0.24	-	$[M-H]^-$	PA(34:2)	$C_{37}H_{69}O_8P$		79, 153, 255, 279, 391, 409, 671
	17	673.48154	673.48144	673.48138	0.24	0.09	$[M-H]^-$	PA(34:1)	$C_{37}H_{71}O_8P$		153, 255, 281, 391, 409, 417, 435, 673
	18	675.49735	-	675.49703	0.47	-	$[M-H]^-$	PA(34:0)	$C_{37}H_{73}O_8P$		
	19	695.46513	-	695.46573	-0.86	-	$[M-H]^-$	PA(36:4)	$C_{39}H_{69}O_8P$		
	20	697.48156	-	697.48138	0.26	-	$[M-H]^-$	PA(36:3)	$C_{39}H_{71}O_8P$		279, 281, 415, 417, 433, 435
	21	699.49712	699.49711	699.49703	0.13	0.11	$[M-H]^-$	PA(36:2)	$C_{39}H_{73}O_8P$	21	78, 153, 279, 283, 415, 419, 433, 437, 699
	22	701.51256	701.51232	701.51268	-0.17	-0.51	$[M-H]^-$	PA(36:1)	$C_{39}H_{75}O_8P$	21	79, 153, 281, 283, 417, 419, 435, 437, 701
	23	687.53378	687.53347	687.53342	0.52	0.07	$[M-H]^-$	PA(O-36:1)	$C_{39}H_{77}O_7P$		
	24	703.52851	703.52827	703.52833	0.26	-0.09	$[M-H]^-$	PA(36:0)	$C_{39}H_{77}O_8P$		
	25	719.46599	-	719.46573	0.36	-	$[M-H]^-$	PA(38:6)	$C_{41}H_{69}O_8P$		153, 255, 283, 391, 409, 463, 481, 719
	26	721.48119	721.48149	721.48138	-0.26	0.15	$[M-H]^-$	PA(38:5)	$C_{41}H_{71}O_8P$		
	27	723.49748	723.46990	723.49703	0.62	-0.18	$[M-H]^-$	PA(38:4)	$C_{41}H_{73}O_8P$		153, 259, 283, 303, 419, 437, 439, 457, 723
	28	725.51278	725.51277	725.51268	0.14	0.12	$[M-H]^-$	PA(38:3)	$C_{41}H_{75}O_8P$		
	29	727.52855	727.52822	727.52833	0.30	-0.15	$[M-H]^-$	PA(38:2)	$C_{41}H_{77}O_8P$		
	30	729.54399	729.54379	729.54398	0.01	-0.26	$[M-H]^-$	PA(38:1)	$C_{41}H_{79}O_8P$		
	31	745.48168	745.48105	745.48138	0.40	-0.44	$[M-H]^-$	PA(40:7)	$C_{43}H_{71}O_8P$		
	32	747.49694	747.49741	747.49703	-0.12	0.51	$[M-H]^-$	PA(40:6)	$C_{43}H_{73}O_8P$		153, 283, 327, 419, 437, 463, 481, 747
	33	749.51266	749.51271	749.51268	-0.03	0.04	$[M-H]^-$	PA(40:5)	$C_{43}H_{75}O_8P$		153, 283, 329, 419, 437, 465, 483, 749

Classification	No.	Measured *m/z*		Calculated *m/z*	Error (ppm)		Assignment			Ref.	Structurally specific CID ions (*m/z*)[a)]
		MCAEF	Common spray		MCAEF	Common spray	Ion form	Compound	Molecular formula		
	34	755.55931	-	755.55963	-0.42	-	$[M-H]^-$	PA(40:2)	$C_{43}H_{81}O_8P$		
	35	795.53092	-	795.53116	-0.30	-	$[M+K-2H]^-$	PA(40:1)	$C_{43}H_{83}O_8P$		
	36	821.54659	821.54614	821.54681	-0.27	-0.82	$[M+K-2H]^-$	PA(42:2)	$C_{45}H_{85}O_8P$		
	37	771.62783	-	771.62732	0.66	-	$[M-H]^-$	PA(P-42:0)	$C_{45}H_{89}O_7P$		
	38	795.49707	-	795.49703	0.05	-	$[M-H]^-$	PA(44:10)	$C_{47}H_{73}O_8P$		
Phosphoglycerols (PGs)											
	1	483.27263	-	483.27284	-0.43	-	$[M-H]^-$	PG(16:0)	$C_{22}H_{45}O_9P$		153, 255, 392, 410, 483
	2	509.28872	-	509.28849	0.45	-	$[M-H]^-$	PG(18:1)	$C_{24}H_{47}O_9P$		153, 281, 417,435, 509
	3	511.30416	511.30425	511.30414	0.04	0.22	$[M-H]^-$	PG(18:0)	$C_{24}H_{49}O_9P$		79, 153, 227, 283, 419, 437, 511
	4	531.27282	-	531.27284	-0.04	-	$[M-H]^-$	PG(20:4)	$C_{26}H_{45}O_9P$		153, 259, 303, 439, 457, 531
	5	555.27267	-	555.27284	-0.31	-	$[M-H]^-$	PG(22:6)	$C_{28}H_{45}O_9P$		
	6	703.49173	-	703.49194	-0.30	-	$[M-H]^-$	PG(P-32:1)	$C_{38}H_{73}O_9P$		
	7	745.50264	745.50267	745.50251	0.17	0.21	$[M-H]^-$	PG(34:2)	$C_{40}H_{75}O_{10}P$		
	8	747.51834	747.51828	747.51816	0.24	0.16	$[M-H]^-$	PG(34:1)	$C_{40}H_{77}O_{10}P$		
	9	769.50263	-	769.50251	0.16	-	$[M-H]^-$	PG(36:4)	$C_{42}H_{75}O_{10}P$		
	10	771.51878	-	771.51816	0.80	-	$[M-H]^-$	PG(36:3)	$C_{42}H_{77}O_{10}P$		
	11	773.53375	773.53364	773.53381	-0.08	-0.22	$[M-H]^-$	PG(36:2)	$C_{42}H_{79}O_{10}P$		
	12	795.51838	-	795.51816	0.28	-	$[M-H]^-$	PG(38:5)	$C_{44}H_{77}O_{10}P$		
	13	797.53389	797.53343	797.53381	0.10	-0.48	$[M-H]^-$	PG(38:4)	$C_{44}H_{79}O_{10}P$		153, 259, 283, 303, 419, 437, 439, 457, 493, 511, 513, 531, 798
	14	821.53374	-	821.53381	-0.09	-	$[M-H]^-$	PG(40:6)	$C_{46}H_{79}O_{10}P$	21	153, 283, 327, 419, 437, 463, 481, 493, 511, 537, 555, 822

Classification	No.	Measured *m/z*		Calculated *m/z*	Error (ppm)		Assignment			Ref.	Structurally specific CID ions (*m/z*)[a)]
		MCAEF	Common spray		MCAEF	Common spray	Ion form	Compound	Molecular formula		
	15	823.54934	-	823.54946	-0.15	-	$[M-H]^-$	PG(40:5)	$C_{46}H_{81}O_{10}P$		
	16	815.61708	-	815.61715	-0.09	-	$[M-H]^-$	PG(P-40:1)	$C_{46}H_{89}O_9P$		
	17	871.52662	871.52612	871.52608	0.62	0.05	$[M+K-2H]^-$	PG(P-42:6)	$C_{48}H_{83}O_9P$		
	18	865.63092	-	865.63039	0.61	-	$[M+Na-2H]^-$	PG(P-42:1)	$C_{48}H_{93}O_9P$		
	19	881.62792	-	881.62771	0.24	-	$[M-H]^-$	PG(44:4)	$C_{50}H_{91}O_{10}P$		
	20	909.65653	909.65650	909.65660	-0.07	-0.11	$[M+Na-2H]^-$	PG(44:1)	$C_{50}H_{97}O_{10}P$		
Phosphatidylserines (PSs)											
	1	496.26825	496.26794	496.26809	0.32	-0.30	$[M-H]^-$	PS(16:0)	$C_{22}H_{44}NO_9P$		79, 153, 255, 409, 496
	2	518.25263	-	518.25244	0.37	-	$[M-H]^-$	PS(18:3)	$C_{24}H_{42}NO_9P$		
	3	544.26813	544.26824	544.26809	0.07	0.28	$[M-H]^-$	PS(20:4)	$C_{26}H_{44}NO_9P$		79, 153, 259, 303, 457, 544
	4	568.26784	568.26783	568.26809	-0.44	-0.46	$[M-H]^-$	PS(22:6)	$C_{28}H_{44}NO_9P$		
	5	570.28325	-	570.28374	-0.86	-	$[M-H]^-$	PS(22:5)	$C_{28}H_{46}NO_9P$		
	6	698.40341	-	698.40386	-0.64	-	$[M-H]^-$	PS(30:4)	$C_{36}H_{62}NO_{10}P$		
	7	762.52911	762.52924	762.52906	0.07	0.24	$[M-H]^-$	PS(34:0)	C40H78NO10P	[21, 38]	
	8	778.46657	-	778.46646	0.14	-	$[M-H]^-$	PS(36:6)	$C_{42}H_{70}NO_{10}P$		
	9	780.48239	-	780.48211	0.36	-	$[M-H]^-$	PS(36:5)	$C_{42}H_{72}NO_{10}P$		
	10	786.52970	786.52938	786.52906	0.81	0.41	$[M-H]^-$	PS(36:2)	$C_{42}H_{78}NO_{10}P$	[21, 38]	79, 153, 281, 417, 435, 700, 786
	11	788.54424	788.54489	788.54471	-0.60	0.23	$[M-H]^-$	PS(36:1)	$C_{42}H_{80}NO_{10}P$	[21, 38]	153, 281, 283, 417, 419, 435, 437, 701, 789
	12	810.52971	810.52910	810.52906	0.80	0.05	$[M-H]^-$	PS(38:4)	$C_{44}H_{78}NO_{10}P$	[21, 38]	79, 283, 303, 419, 437, 457, 723, 810
	13	812.54430	-	812.54471	-0.50	-	$[M-H]^-$	PS(38:3)	$C_{44}H_{80}NO_{10}P$		
	14	814.56046	-	814.56036	0.12	-	$[M-H]^-$	PS(38:2)	$C_{44}H_{82}NO_{10}P$		
	15	816.57622	816.57642	816.57601	0.26	0.50	$[M-H]^-$	PS(38:1)	$C_{44}H_{84}NO_{10}P$	[38]	

Classification	No.	Measured m/z		Calculated m/z	Error (ppm)		Assignment			Ref.	Structurally specific CID ions (m/z)[a)]
		MCAEF	Common spray		MCAEF	Common spray	Ion form	Compound	Molecular formula		
	16	818.59126	818.59148	818.59166	-0.49	-0.22	$[M-H]^-$	PS(38:0)	$C_{44}H_{86}NO_{10}P$		
	17	826.46675	-	826.46646	0.35	-	$[M-H]^-$	PS(40:10)	$C_{46}H_{70}NO_{10}P$		
	18	828.48247	-	828.48211	0.43	-	$[M-H]^-$	PS(40:9)	$C_{46}H_{72}NO_{10}P$		
	19	830.49767	-	830.49776	-0.11	-	$[M-H]^-$	PS(40:8)	C46H74NO10P		
	20	832.51327	-	832.51341	-0.17	-	$[M-H]^-$	PS(40:7)	$C_{46}H_{76}NO_{10}P$		
	21	834.52890	834.52845	834.52906	-0.19	-0.73	$[M-H]^-$	PS(40:6)	$C_{46}H_{78}NO_{10}P$	21, 38	79, 153, 283, 419,437, 463, 748, 834
		872.48488	872.48425	872.48494	-0.07	-0.79	$[M+K-2H]^-$				
	22	836.54529	-	836.54471	0.69	-	$[M-H]^-$	PS(40:5)	$C_{46}H_{80}NO_{10}P$		
	23	838.56020	-	838.56036	-0.19	-	$[M-H]^-$	PS(40:4)	$C_{46}H_{82}NO_{10}P$		
	24	840.57574	-	840.57601	-0.32	-	$[M-H]^-$	PS(40:3)	$C_{46}H_{84}NO_{10}P$		
	25	842.59140	842.59178	842.59166	-0.31	0.14	$[M-H]^-$	PS(40:2)	$C_{46}H_{86}NO_{10}P$		
	26	844.60758	844.60735	844.60731	0.32	0.05	$[M-H]^-$	PS(40:1)	$C_{46}H_{88}NO_{10}P$		
	27	854.49754	-	854.49776	-0.26	-	$[M-H]^-$	PS(42:10)	$C_{48}H_{74}NO_{10}P$		
	28	856.51357	-	856.51341	0.19	-	$[M-H]^-$	PS(42:9)	$C_{48}H_{76}NO_{10}P$		
	29	858.52904	-	858.52906	-0.02	-	$[M-H]^-$	PS(42:8)	$C_{48}H_{78}NO_{10}P$		
	30	864.57560	-	864.57601	-0.47	-	$[M-H]^-$	PS(42:5)	$C_{48}H_{84}NO_{10}P$		
	31	892.60793	892.60812	892.60731	0.69	0.91	$[M-H]^-$	PS(44:5)	$C_{50}H_{88}NO_{10}P$		
	32	894.62284	894.62261	894.62296	-0.13	-0.39	$[M-H]^-$	PS(44:4)	$C_{50}H_{90}NO_{10}P$		
	33	896.63850	-	896.63861	-0.12	-	$[M-H]^-$	PS(44:3)	$C_{50}H_{92}NO_{10}P$		
	34	898.65431	898.65400	898.65426	0.06	-0.29	$[M-H]^-$	PS(44:2)	$C_{50}H_{94}NO_{10}P$		
Phosphatidylinositols (PIs)											
	1	571.28854	-	571.28889	-0.61	-	$[M-H]^-$	PI(16:0)	$C_{25}H_{49}O_{12}P$		79, 153, 223, 241, 255, 315, 333, 391, 409, 571
	2	595.28874	-	595.28889	-0.27	-	$[M-H]^-$	PI(18:2)	$C_{27}H_{49}O_{12}P$		
	3	597.30455	-	597.30454	0.02	-	$[M-H]^-$	PI(18:1)	$C_{27}H_{51}O_{12}P$		153, 223, 241, 281, 315, 333, 417, 435,

Classification	No.	Measured m/z		Calculated m/z	Error (ppm)		Assignment			Ref.	Structurally specific CID ions (m/z)[a]
		MCAEF	Common spray		MCAEF	Common spray	Ion form	Compound	Molecular formula		
											597
	4	599.32029	599.32015	599.32019	0.17	-0.07	$[M-H]^-$	PI(18:0)	$C_{27}H_{53}O_{12}P$		
	5	617.27320	-	617.27324	-0.06	-	$[M-H]^-$	PI(20:5)	$C_{29}H_{47}O_{12}P$		
	6	619.28890	-	619.28889	0.02	-	$[M-H]^-$	PI(20:4)	$C_{29}H_{49}O_{12}P$		153,223, 241, 259, 303, 315, 333, 439, 457, 619
	7	651.35191	-	651.35149	0.64	-	$[M-H]^-$	PI(22:2)	$C_{31}H_{57}O_{12}P$		
	8	653.36717	-	653.36714	0.05	-	$[M-H]^-$	PI(22:1)	$C_{31}H_{59}O_{12}P$		
	9	655.38241	655.38284	655.38279	-0.58	0.08	$[M-H]^-$	PI(22:0)	$C_{31}H_{61}O_{12}P$		
	10	803.47170	-	803.47160	0.12	-	$[M-H]^-$	PI(32:3)	$C_{41}H_{73}O_{13}P$		
	11	805.48788	-	805.48725	0.78	-	$[M-H]^-$	PI(32:2)	$C_{41}H_{75}O_{13}P$		
	12	807.50291	807.50299	807.50290	0.01	0.11	$[M-H]^-$	PI(32:1)	$C_{41}H_{77}O_{13}P$		
	13	833.51862	-	833.51855	0.08	-	$[M-H]^-$	PI(34:2)	$C_{43}H_{79}O_{13}P$		
	14	835.53490	835.53463	835.53420	0.84	0.51	$[M-H]^-$	PI(34:1)	$C_{43}H_{81}O_{13}P$		
	15	837.54993	837.54966	837.54985	0.10	-0.23	$[M-H]^-$	PI(34:0)	$C_{43}H_{83}O_{13}P$		
	16	823.57069	823.57020	823.57059	0.12	-0.47	$[M-H]^-$	PI(O-34:0)	$C_{43}H_{85}O_{12}P$		
	17	855.50296	-	855.50290	0.07	-	$[M-H]^-$	PI(36:5)	$C_{45}H_{77}O_{13}P$		
	18	857.51825	857.51806	857.51855	-0.35	-0.57	$[M-H]^-$	PI(36:4)	$C_{45}H_{79}O_{13}P$	21, 38, 39	
	19	861.54973	861.54949	861.54985	-0.14	-0.42	$[M-H]^-$	PI(36:2)	$C_{45}H_{83}O_{13}P$		153, 223, 241, 281, 417, 435, 579, 597, 700, 861
	20	863.56513	863.56592	863.56550	-0.43	0.49	$[M-H]^-$	PI(36:1)	$C_{45}H_{85}O_{13}P$		
		899.54236	-	899.54218	0.20	-	$[M+Cl]^-$				
	21	865.58191	-	865.58115	0.88	-	$[M-H]^-$	PI(36:0)	$C_{45}H_{87}O_{13}P$		
		901.55756	-	901.55783	-0.30	-	$[M+Cl]^-$				
	22	881.51829	881.51895	881.51855	-0.29	0.45	$[M-H]^-$	PI(38:6)	$C_{47}H_{79}O_{13}P$		
	23	867.53918	-	867.53929	-0.13	-	$[M-H]^-$	PI(O-38:6)	$C_{47}H_{81}O_{12}P$		
	24	883.53417	883.53402	883.53420	-0.03	-0.20	$[M-H]^-$	PI(38:5)	$C_{47}H_{81}O_{13}P$	39	
	25	885.54974	885.54976	885.54985	-0.12	-0.10	$[M-H]^-$	PI(38:4)	$C_{47}H_{83}O_{13}P$	21, 26, 38, 39	240, 259, 283, 303, 419, 437, 439, 457, 581, 599, 601, 619, 886

Classification	No.	Measured m/z		Calculated m/z	Error (ppm)		Assignment			Ref.	Structurally specific CID ions (m/z)$^{a)}$
		MCAEF	Common spray		MCAEF	Common spray	Ion form	Compound	Molecular formula		
	26	887.56534	887.56587	887.56550	-0.18	0.42	[M-H]$^-$	PI(38:3)	$C_{47}H_{85}O_{13}P$		
	27	889.58118	889.58147	889.58115	0.03	0.36	[M-H]$^-$	PI(38:2)	$C_{47}H_{87}O_{13}P$		
	28	891.59648	891.59658	891.59680	-0.36	-0.25	[M-H]$^-$	PI(38:1)	$C_{47}H_{89}O_{13}P$		
	29	901.48751	-	901.48725	0.29	-	[M-H]$^-$	PI(40:10)	$C_{49}H_{75}O_{13}P$		
	30	903.50314	-	903.50290	0.27	-	[M-H]$^-$	PI(40:9)	$C_{49}H_{77}O_{13}P$		
	31	905.51874	905.51861	905.51855	0.21	0.07	[M-H]$^-$	PI(40:8)	$C_{49}H_{79}O_{13}P$		
	32	907.53428	907.53442	907.53420	0.09	0.24	[M-H]$^-$	PI(40:7)	$C_{49}H_{81}O_{13}P$		
	33	893.55443	-	893.55494	-0.57	-	[M-H]$^-$	PI(P-40:6)	$C_{49}H_{83}O_{12}P$		
	34	909.55026	90955024	909.54985	0.43	0.45	[M-H]$^-$	PI(40:6)	$C_{49}H_{83}O_{13}P$		
	35	911.56555	911.56521	911.56550	0.05	-0.32	[M-H]$^-$	PI(40:5)	$C_{49}H_{85}O_{13}P$		
	36	913.58147	913.58133	913.58115	0.35	0.20	[M-H]$^-$	PI(40:4)	$C_{49}H_{87}O_{13}P$		
	37	915.59657	-	915.59680	-0.25	-	[M-H]$^-$	PI(40:3)	$C_{49}H_{89}O_{13}P$		
	38	917.61244	-	917.61245	-0.01	-	[M-H]$^-$	PI(40:2)	$C_{49}H_{91}O_{13}P$		
	39	919.62872	919.62880	919.62810	0.67	0.76	[M-H]$^-$	PI(40:1)	$C_{49}H_{93}O_{13}P$		
	40	921.64380	921.64369	921.64375	0.05	-0.07	[M-H]$^-$	PI(40:0)	$C_{49}H_{95}O_{13}P$		
	41	927.50281	-	927.50290	-0.10	-	[M-H]$^-$	PI(42:11)	$C_{51}H_{77}O_{13}P$		
	42	929.51859	-	929.51855	0.04	-	[M-H]$^-$	PI(42:10)	$C_{51}H_{79}O_{13}P$		
	43	931.53434	-	931.53420	0.15	-	[M-H]$^-$	PI(42:9)	$C_{51}H_{81}O_{13}P$		
	44	933.54992	-	933.54985	0.07	-	[M-H]$^-$	PI(42:8)	$C_{51}H_{83}O_{13}P$		
	45	935.56522	935.56513	935.56550	-0.30	-0.40	[M-H]$^-$	PI(42:7)	$C_{51}H_{85}O_{13}P$		
	46	973.55758	-	973.55783	-0.26	-	[M+Cl]$^-$	PI(42:6)	$C_{51}H_{87}O_{13}P$		
		975.53752	-	975.53704	0.49	-	[M+K-2H]$^-$				
	47	939.59623	-	939.59680	-0.61	-	[M-H]$^-$	PI(42:5)	$C_{51}H_{89}O_{13}P$		
		975.57352	975.57320	975.57348	0.04	-0.29	[M+Cl]$^-$				
	48	943.62831	-	943.62810	0.22	-	[M-H]$^-$	PI(42:3)	$C_{51}H_{93}O_{13}P$		
	49	957.54990	-	957.54985	0.05	-	[M-H]$^-$	PI(44:10)	$C_{53}H_{83}O_{13}P$		
	50	959.56571	-	959.56550	0.21	-	[M-H]$^-$	PI(44:9)	$C_{53}H_{85}O_{13}P$		
	51	961.58170	961.58134	961.58115	0.57	0.20	[M-H]$^-$	PI(44:8)	$C_{53}H_{87}O_{13}P$		
	52	1001.55290	-	1001.55269	0.21	-	[M+K-2H]$^-$	PI(44:7)	$C_{53}H_{89}O_{13}P$		

Classification	No.	Measured *m/z*		Calculated *m/z*	Error (ppm)		Assignment			Ref.	Structurally specific CID ions (*m/z*)[a)]
		MCAEF	Common spray		MCAEF	Common spray	Ion form	Compound	Molecular formula		
	53	965.61223	-	965.61245	-0.23	-	$[M-H]^-$	PI(44:6)	$C_{53}H_{91}O_{13}P$		
		1003.56866	-	1003.56834	0.32	-	$[M+K-2H]^-$				
	54	971.65902	-	971.65940	-0.39	-	$[M-H]^-$	PI(44:3)	$C_{53}H_{97}O_{13}P$		
Phosphatidylinositol monophosphates (PIPs)											
	1	915.50046	915.50076	915.50053	-0.08	0.25	$[M-H]^-$	PIP(34:1)	$C_{43}H_{82}O_{16}P_2$		
	2	917.51644	-	917.51618	0.28	-	$[M-H]^-$	PIP(34:0)	$C_{43}H_{84}O_{16} P_2$		
	3	937.48494	-	937.48488	0.06	-	$[M-H]^-$	PIP(36:4)	$C_{45}H_{80}O_{16}P_2$		
	4	939.50046	939.50074	939.50053	-0.07	0.22	$[M-H]^-$	PIP(36:3)	$C_{45}H_{82}O_{16} P_2$		
	5	941.51634	941.51624	941.51618	0.17	0.06	$[M-H]^-$	PIP(36:2)	$C_{45}H_{84}O_{16}P_2$		
	6	943.53178	-	943.53183	-0.05	-	$[M-H]^-$	PIP(36:1)	$C_{45}H_{86}O_{16}P_2$		
	7	915.50029	915.50068	915.50053	-0.26	0.16	$[M-H]^-$	PIP(34:1)	$C_{47}H_{82}O_{16} P_2$		
	8	963.50084	-	963.50053	0.32	-	$[M-H]^-$	PIP(38:5)	$C_{47}H_{82}O_{16} P_2$		
	9	965.51622	-	965.51618	0.04	-	$[M-H]^-$	PIP(38:4)	$C_{47}H_{84}O_{16}P_2$	21	
		987.49832	-	987.49813	0.19	-	$[M+Na-2H]^-$				
		1003.47243	1003.47275	1003.47207	0.36	0.68	$[M+K-2H]^-$				
	10	967.53174	-	967.53183	-0.09	-	$[M-H]^-$	PIP(38:3)	$C_{47}H_{86}O_{16}P_2$		
	11	969.54758	969.54726	969.54748	0.10	-0.23	$[M-H]^-$	PIP(38:2)	$C_{47}H_{88}O_{16}P_2$		
Phosphatidylinositol bisphosphates (PIP2s)											
	1	965.42003	965.41987	965.41991	0.12	-0.04	$[M-H]^-$	PIP2(32:2)	$C_{41}H_{77}O_{19}P_3$		
	2	967.43631	-	967.43556	0.78	-	$[M-H]^-$	PIP2(32:1)	$C_{41}H_{79}O_{19}P_3$		
	3	969.45124	969.45151	969.45121	0.03	0.31	$[M-H]^-$	PIP2(32:0)	$C_{41}H_{81}O_{19}P_3$		
	4	993.45151	-	993.45121	0.30	-	$[M-H]^-$	PIP2(34:2)	$C_{43}H_{81}O_{19}P_3$		
	5	995.46657	-	995.46686	-0.29	-	$[M-H]^-$	PIP2(34:1)	$C_{43}H_{83}O_{19}P_3$		
	6	997.48219	997.48264	997.48251	-0.32	0.13	$[M-H]^-$	PIP2(34:0)	$C_{43}H_{85}O_{19}P_3$		

Classification	No.	Measured *m/z*		Calculated *m/z*	Error (ppm)		Assignment			Ref.	Structurally specific CID ions (*m/z*)[a)]
		MCAEF	Common spray		MCAEF	Common spray	Ion form	Compound	Molecular formula		
	7	1019.46662	-	1019.46686	-0.23	-	$[M-H]^-$	PIP2(36:3)	$C_{45}H_{83}O_{19}P_3$		
	8	1021.48293	1021.48262	1021.48251	0.41	0.11	$[M-H]^-$	PIP2(36:2)	$C_{45}H_{85}O_{19}P_3$		
	9	1023.49821	1023.49804	1023.49816	0.05	-0.11	$[M-H]^-$	PIP2(36:1)	$C_{45}H_{87}O_{19}P_3$		
	10	1043.46681	-	1043.46686	-0.05	0.55	$[M-H]^-$	PIP2(38:5)	$C_{47}H_{83}O_{19}P_3$		
	11	1045.48265	-	1045.48251		-	$[M-H]^-$	PIP2(38:4)	$C_{47}H_{85}O_{19}P_3$		
	12	1047.49870	1047.49874	1047.49816	0.52	0.55	$[M-H]^-$	PIP2(38:3)	$C_{47}H_{87}O_{19}P_3$		
	13	1049.51333	-	1049.51381	-0.46	-	$[M-H]^-$	PIP2(38:2)	$C_{47}H_{89}O_{19}P_3$		
Glycerophosphoglycero-phosphoglycerols (Cardiolipins)											
	1	1185.73530	1185.73531	1185.73528	0.02	0.03	$[M-H]^-$	CL(1'-[18:2/18:2],3'-[18:2/0:0]) or its isomers	$C_{63}H_{112}O_{16}P_2$		
		1221.71192	-	1221.71196	-0.03	-	$[M+Cl]^-$				
	2	1419.93339	1419.93374	1419.93365	-0.18	0.06	$[M-H]^-$	CL(18:2/18:2/18:2/16:2) or its isomers	$C_{79}H_{138}O_{17}P_2$	39	
	3	1421.94951	-	1421.94930	0.15	-	$[M-H]^-$	CL(18:2/18:2/18:2/16:1) or its isomers	$C_{79}H_{140}O_{17}P_2$	39, 40	
	4	1423.96512	-	1423.96495	0.12	-	$[M-H]^-$	CL(18:2/18:2/18:1/16:1) or its isomers	$C_{79}H_{142}O_{17}P_2$	39-41	
	5	1425.98078	-	1425.98060	0.13	-	$[M-H]^-$	CL(18:2/18:1/18:1/16:1) or its isomers	$C_{79}H_{144}O_{17}P_2$	39-41	
	6	1427.99615	1427.99646	1427.99625	-0.07	0.15	$[M-H]^-$	CL(18:1/18:1/18:1/16:1) or its isomers	$C_{79}H_{146}O_{17}P_2$	39-41	
	7	1430.01174	1430.01204	1430.01190	-0.11	0.10	$[M-H]^-$	CL(18:1/18:1/18:1/16:0) or its isomers	$C_{79}H_{148}O_{17}P_2$	39	
	8	1432.02767	-	1432.02755	0.08	-	[M-H]	CL(18:1/18:1/18:0/16:0) or its isomers	$C_{79}H_{150}O_{17}P_2$	39, 40	
	9	1445.94947	-	1445.94930	0.11	-	$[M-H]^-$	CL(18:2/18:2/18:2/18:3) or its isomers	$C_{81}H_{140}O_{17}P_2$	39, 40	
	10	1447.96481	1447.96510	1447.96495	-0.10	0.10	$[M-H]^-$	CL(18:2/18:2/18:2/18:2) or its isomers	$C_{81}H_{142}O_{17}P_2$	39, 41	
	11	1485.95704	-	1485.95728	-0.16	-	$[M+Cl]^-$	CL(18:2/18:2/18:2/18:1) or its isomers	$C_{81}H_{144}O_{17}P_2$	39, 41	
		1487.93684	-	1487.93648	0.25	-	$[M+K-2H]^-$				
	12	1489.98837	-	1489.98858	-0.14	-	$[M+Cl]^-$	CL(18:2/18:1/18:1/18:1) or its isomers	$C_{81}H_{148}O_{17}P_2$	39, 40	
	13	1493.98318	-	1493.98343	-0.17	-	$[M+K-2H]^-$	CL(18:1/18:1/18:1/18:1) or its isomers	$C_{81}H_{150}O_{17}P_2$	39, 40	
	14	1458.04347	-	1458.04320	0.19	-	$[M-H]^-$	CL(18:1/18:1/18:1/18:0) or its isomers	$C_{81}H_{152}O_{17}P_2$	39	
	15	1460.05887	1460.05849	1460.05885	0.01	-0.25	$[M-H]^-$	CL(18:1/18:1/18:0/18:0) or its isomers	$C_{81}H_{154}O_{17}P_2$	39	
	16	1467.93332	-	1467.93365	-0.22	-	$[M-H]^-$	CL(22:6/18:2/18:2/16:2) or its isomers	$C_{83}H_{138}O_{17}P_2$	39	
	17	1469.949369	-	1469.94930	0.05	-	$[M-H]^-$	CL(22:6/18:2/18:2/16:1) or its isomers	$C_{83}H_{140}O_{17}P_2$	39	
	18	1471.96440	1471.96474	1471.96495	-0.37	-0.14	$[M-H]^-$	CL(22:6/18:2/18:1/16:1) or its isomers	$C_{83}H_{142}O_{17}P_2$	39	

Classification	No.	Measured *m/z*		Calculated *m/z*	Error (ppm)		Assignment			Ref.	Structurally specific CID ions (*m/z*)$^{a)}$
		MCAEF	Common spray		MCAEF	Common spray	Ion form	Compound	Molecular formula		
	19	1473.98050	1473.98071	1473.98060	-0.07	0.07	$[M-H]^-$	CL(22:6/18:2/18:1/16:0) or its isomers	$C_{83}H_{144}O_{17}P_2$	39, 40	
		1511.93698	-	1511.93648	0.33	-	$[M+K-2H]^-$				
	20	1475.99691	-	1475.99625	0.45	-	$[M-H]^-$	CL(22:6/18:1/18:1/16:0) or its isomers	$C_{83}H_{146}O_{17}P_2$	39-41	
	21	1478.01117	-	1478.01190	-0.49	-	$[M-H]^-$	CL(20:4/18:1/18:1/18:1) or its isomers	$C_{83}H_{148}O_{17}P_2$	39, 40	
		1513.98828	-	1513.98858	-0.20	-	$[M+Cl]^-$				
	22	1515.96791	1515.96754	1515.96778	0.09	-0.16	$[M+K-2H]^-$				
	23	1480.02764	-	1480.02755	0.06	-	[M-H]	CL(20:4/18:1/18:1/18:0) or its isomers	$C_{83}H_{150}O_{17}P_2$	39, 40	
	24	1482.04324	-	1482.04320	0.03	-	[M-H]	CL(20:4/18:1/18:0/18:0) or its isomers	$C_{83}H_{152}O_{17}P_2$	39	
	25	1484.05957	-	1484.05885	0.49	-	[M-H]	CL(20:4/18:0/18:0/18:0) or its isomers	$C_{83}H_{154}O_{17}P_2$	39	
	26	1491.93331	1491.93345	1491.93365	-0.23	-0.13	$[M-H]^-$	CL(22:6/20:4/18:2/16:2) or its isomers	$C_{85}H_{138}O_{17}P_2$	39	
	27	1493.94951	-	1493.94930	0.14	-	$[M-H]^-$	CL(22:6/20:4/18:2/16:1) or its isomers	$C_{85}H_{140}O_{17}P_2$	39, 40	
	28	1495.96465	1495.96485	1495.96495	-0.20	-0.07	$[M-H]^-$	CL(22:6/20:3/18:2/16:1) or its isomers	$C_{85}H_{142}O_{17}P_2$	39, 40	
	29	1497.98067	-	1497.98060	0.05	-	$[M-H]^-$	CL(22:6/18:2/18:2/18:1) or its isomers	$C_{85}H_{144}O_{17}P_2$	39-41	
	30	1499.99671	-	1499.99625	0.31	-	$[M-H]^-$	CL(22:6/18:2/18:2/18:0) or its isomers	$C_{85}H_{146}O_{17}P_2$	39-41	
	31	1502.01112	-	1502.01190	-0.52	-	$[M-H]^-$	CL(22:6/18:2/18:1/18:0) or its isomers	$C_{85}H_{148}O_{17}P_2$	39-41	
	32	1565.94644	-	1565.94689	-0.29	-	$[M+Na-2H]^-$	CL(22:6/22:6/18:2/18:2) or its isomers	$C_{89}H_{142}O_{17}P_2$		
Cyclic phosphatidic acids (cPAs)											
	1	391.22561	391.22569	391.22550	0.28	0.49	$[M-H]^-$	CPA(16:0)	$C_{19}H_{37}O_6P$		
	2	415.22553	415.22531	415.22550	0.07	-0.46	$[M-H]^-$	CPA(18:2)	$C_{21}H_{37}O_6P$		
	3	417.24139	417.24118	417.24115	0.58	0.07	$[M-H]^-$	CPA(18:1)	$C_{21}H_{39}O_6P$		
	4	419.25698	419.25684	419.25680	0.43	0.10	$[M-H]^-$	CPA(18:0)	$C_{21}H_{41}O_6P$		
CDP-Glycerols											
	1	952.50714	-	952.50702	0.13	-	$[M-H]^-$	CDP-DG(32:0)	$C_{44}H_{81}N_3O_{15}P_2$		
	2	1004.53841	-	1004.53832	0.09	-	$[M-H]^-$	CDP-DG(36:2)	$C_{48}H_{85}N_3O_{15}P_2$		159, 215, 273, 281, 304, 322, 384, 417, 479, 497, 700, 722, 762, 780, 894, 962, 1005

Classification	No.	Measured m/z		Calculated m/z	Error (ppm)		Assignment			Ref.	Structurally specific CID ions (m/z)[a)]
		MCAEF	Common spray		MCAEF	Common spray	Ion form	Compound	Molecular formula		
	3	1024.50762	1024.50739	1024.50702	0.59	0.36	$[M-H]^-$	CDP-DG(38:6)	$C_{50}H_{81}N_3O_{15}P_2$		
	4	1026.52250	-	1026.52267	-0.17	-	$[M-H]^-$	CDP-DG(38:5)	$C_{50}H_{83}N_3O_{15}P_2$		
	5	1028.53828	1028.53890	1028.53832	-0.04	0.56	$[M-H]^-$	CDP-DG(38:4)	$C_{50}H_{85}N_3O_{15}P_2$		
	6	1066.53094	-	1066.53064	0.28	-	$[M+Cl]^-$	CDP-DG(38:3)	$C_{50}H_{87}N_3O_{15}P_2$		
	7	1050.52254	1050.52273	1050.52267	-0.12	0.06	$[M-H]^-$	CDP-DG(40:7)	$C_{52}H_{83}N_3O_{15}P_2$		
	8	1052.53873	-	1052.53832	0.39	-	$[M-H]^-$	CDP-DG(40:6)	$C_{52}H_{85}N_3O_{15}P_2$		
		1088.51433	-	1088.51499	-0.61	-	$[M+Cl]^-$				
	9	1056.56972	-	1056.56962	0.09	-	$[M-H]^-$	CDP-DG(40:4)	$C_{52}H_{89}N_3O_{15}P_2$		
Glycerophosphate											
	1	423.25179	-	423.25171	0.19	-	$[M-H]^-$	1-heptadecanoyl-glycero-3-phosphate or its isomers	$C_{20}H_{41}O_7P$		
**Sphingolipids**											
Ceramides (Cers)											
	1	500.40825	-	500.40851	-0.52	-	$[M+Na-2H]^-$	Cer(d30:2)	$C_{30}H_{57}NO_3$		
	2	616.47139	-	616.47115	0.39	-	$[M-H]^-$	CerP(d34:1)	$C_{34}H_{68}NO_6P$		
	3	644.50271	644.50191	644.50245	0.40	-0.84	$[M-H]^-$	CerP(d36:1)	$C_{36}H_{72}NO_6P$		
	4	726.58063	-	726.58070	-0.10	-	$[M-H]^-$	CerP(d42:2)	$C_{42}H_{82}NO_6P$		264, 749, 767
Sphingomyelins (SMs)											
	1	793.56321	-	793.56313	0.10	-	$[M+K-2H]^-$	SM(d38:2)	$C_{43}H_{85}N_2O_6P$		
	2	785.65433	-	785.65420	0.17	-	$[M-H]^-$	SM(d40:1)	$C_{45}H_{91}N2O_6P$		
Ceramide phosphoinositols (PI-Cers)											
	1	808.57074	-	808.57092	-0.22	-	$[M-H]^-$	PI-Cer(d36:0)	$C_{42}H_{84}NO_{11}P$		
	2	852.59715	-	852.59714	0.01	-	$[M-H]^-$	PI-Cer(d38:0(2OH))	$C_{44}H_{88}NO_{12}P$		
	3	958.58752	-	958.58736	0.17	-	$[M-H]^-$	MIPC(d34:0(2OH))	$C_{46}H_{90}NO_{17}P$		

Classification	No.	Measured *m/z*		Calculated *m/z*	Error (ppm)		Assignment			Ref.	Structurally specific CID ions (*m/z*)[a)]
		MCAEF	Common spray		MCAEF	Common spray	Ion form	Compound	Molecular formula		
	4	986.61803	-	986.61866	-0.64	-	$[M-H]^-$	MIPC(d36:0(2OH))	$C_{48}H_{94}NO_{17}P$		
	5	1002.61363	1002.61320	1002.61358	0.05	-0.38	$[M-H]^-$	MIPC(t36:0(2OH))	$C_{48}H_{94}NO_{18}P$		
	6	952.68575	-	952.68595	-0.21	-	$[M-H]^-$	PI-Cer(t44:0(2OH))	$C_{50}H_{100}NO_{13}P$		
	7	1052.60586	1052.60556	1052.60584	0.02	-0.27	$[M+K-2H]^-$	MIPC(d38:0(2OH))	$C_{50}H_{98}NO_{17}P$		
	8	1068.60103	-	1068.60076	0.25	-	$[M+K-2H]^-$	MIPC(t38:0(2OH))	$C_{50}H_{98}NO_{18}P$		
	9	1080.63744	-	1080.63714	0.28	-	$[M+K-2H]^-$	MIPC(d40:0(2OH))	$C_{52}H_{102}NO_{17}P$		
	10	1096.63206	-	1096.63206	0.00	-	$[M+K-2H]^-$	MIPC(t40:0(2OH))	$C_{52}H_{102}NO_{18}P$		
	11	980.71714	-	980.71725	-0.11	-	$[M-H]^-$	PI-Cer(t46:0(2OH))	$C_{52}H_{104}NO_{13}P$		
	12	1212.64271	-	1212.64290	-0.16	-	$[M-H]^-$	M(IP)2C(d36:0)	$C_{54}H_{105}NO_{24}P_2$		
	13	1228.63757	-	1228.63781	-0.20	-	$[M-H]^-$	M(IP)2C(t36:0)	$C_{54}H_{105}NO_{25}P_2$		
	14	1244.63212	-	1244.63273	-0.49	-	$[M-H]^-$	M(IP)2C(t36:0(2OH))	$C_{54}H_{105}NO_{26}P_2$		
	15	1108.66841	-	1108.66844	-0.03	-	$[M+K-2H]^-$	MIPC(d42:0(2OH))	$C_{54}H_{106}NO_{17}P$		
	16	1108.68968	-	1108.68942	0.23	-	$[M+Na-2H]^-$	MIPC(t42:0(2OH))	$C_{54}H_{106}NO_{18}P$		
	17	1136.69941	1136.69950	1136.69974	-0.29	-0.21	$[M+K-2H]^-$	MIPC(d44:0(2OH))	$C_{56}H_{110}NO_{17}P$		
Neutral glycosphingolipids											
	1	642.49509	-	642.49504	0.08	-	$[M-H]^-$	GlcCer(d30:1)	$C_{36}H_{69}NO_8$		
	2	670.52631	670.52676	670.52634	-0.04	0.63	$[M-H]^-$	GlcCer(d32:1)	$C_{36}H_{73}NO_8$		
	3	698.55721	-	698.55764	-0.62	-	$[M-H]^-$	GlcCer(d34:1)	$C_{40}H_{77}NO_8$		
	4	740.56835	-	740.56821	0.19	-	$[M-H]^-$	GlcCer(d38:2-OH)	$C_{42}H_{79}NO_9$		
	5	868.55522	868.55576	868.55584	-0.71	-0.09	$[M+Cl]^-$	LacCer(d32:1)	$C_{44}H_{83}NO_{13}$		
	6	754.62023	-	754.62024	-0.01	-	$[M-H]^-$	GlcCer(d38:1)	$C_{44}H_{85}NO_8$		
	7	936.64182	-	936.64177	0.05	-	$[M-H]^-$	FMC-5(d36:1)	$C_{52}H_{91}NO_{13}$		
	8	998.75156	998.75161	998.75132	0.24	0.29	$[M-H]^-$	LacCer(d44:2)	$C_{56}H_{105}NO_{13}$		
	9	1058.63951	-	1058.63997	-0.43	-	$[M+Cl]^-$	Galα1-4Galβ1-4Glcβ-Cer(d18:1/16:0) or its isomers	$C_{52}H_{97}NO_{18}$		

Classification	No.	Measured *m/z*		Calculated *m/z*	Error (ppm)		Assignment			Ref.	Structurally specific CID ions (*m/z*)[a)]
		MCAEF	Common spray		MCAEF	Common spray	Ion form	Compound	Molecular formula		
	10	1108.68937	-	1108.68942	-0.05	-	$[M+Na-2H]^-$	Galα1-4Galβ1-4Glcβ-Cer(d18:1/22:0) or its isomers	$C_{54}H_{106}NO_{18}P$		
	11	1100.70789	-	1100.70783	0.05	-	$[M+Na-2H]^-$	Galα1-4Galβ1-4Glcβ-Cer(d18:1/20:0) or its isomers	$C_{56}H_{105}NO_{18}$		
	12	1190.68278	-	1190.68216	0.52	-	$[M+K-2H]^-$	Fucα1-2Galα1-3Galβ1-4Glcβ-Cer(d18:1/16:0) or its isomers	$C_{58}H_{107}NO_{21}$		
	13	1206.67716	-	1206.67708	0.07	-	$[M+K-2H]^-$	Galα1-3(Fucα1-2)Galβ1-4Glcβ-Cer(d18:1/16:0) or its isomers	$C_{58}H_{107}NO_{22}$		
	14	1225.74260	1225.74275	1225.74266	-0.05	0.07	$[M-H]^-$	GalNAcβ1-3Galα1-4Galβ1-4Glcβ-Cer(d18:1/16:0) or its isomers	$C_{60}H_{110}N_2O_{23}$		
	15	1218.71364	-	1218.71347	0.14	-	$[M+K-2H]^-$	Fucα1-2Galα1-3Galβ1-4Glcβ-Cer(d18:1/18:0) or its isomers	$C_{60}H_{111}NO_{21}$		
	16	1253.77410	-	1253.77396	0.11	-	$[M-H]^-$	Galβ1-4GlcNAcβ1-3Galβ1-4Glcβ-Cer(d18:1/18:0) or its isomers	$C_{62}H_{114}N_2O_{23}$		
	17	1281.80543	-	1281.80526	0.13	-	$[M-H]^-$	GalNAcβ1-3Galα1-4Galβ1-4Glcβ-Cer(d18:1/20:0) or its isomers	$C_{64}H_{118}N_2O_{23}$		
	18	1387.79556	1387.79541	1387.79549	0.05	-0.06	$[M-H]^-$	Galβ1-3GalNAcβ1-3Galα1-4Galβ1-4Glcβ-Cer(d18:1/16:0) or its isomers	$C_{66}H_{120}N_2O_{28}$		
	19	1278.83064	-	1278.83075	-0.09	-	$[M-H]^-$	Galα1-3(Fucα1-2)Galβ1-4Glcβ-Cer(d18:1/24:1) or its isomers	$C_{66}H_{121}NO_{22}$		
	20	1309.83673	-	1309.83656	0.13	-	$[M-H]^-$	Galβ1-4GlcNAcβ1-3Galβ1-4Glcβ-Cer(d18:1/22:0) or its isomers	$C_{66}H_{122}N_2O_{23}$		
	21	1280.84637	1280.84651	1280.84640	-0.02	0.09	$[M-H]^-$	Galα1-3(Fucα1-2)Galβ1-4Glcβ-Cer(d18:1/24:0) or its isomers	$C_{66}H_{123}NO_{22}$		
	22	1296.84146	1296.84127	1296.84131	0.12	-0.03	$[M-H]^-$	Galα1-3Galα1-4Galβ1-4Glcβ-Cer(d18:1/24:0) or its isomers	$C_{66}H_{123}NO_{23}$		
	23	1306.86207	-	1306.86205	0.15	-	$[M-H]^-$	Galα1-3(Fucα1-2)Galβ1-4Glcβ-Cer(d18:1/26:1) or its isomers	$C_{68}H_{125}NO_{22}$		
	24	1386.83650	-	1386.83662	-0.09	-	$[M-H]^-$	Fucα1-2Galα1-3Galα1-4Galβ1-4Glcβ-Cer(d18:1/20:0) or its isomers	$C_{68}H_{125}NO_{27}$		
	25	1533.85348	-	1533.85339	0.06	-	$[M-H]^-$	Fucα1-2Galβ1-3GalNAcβ1-3Galα1-4Galβ1-4Glcβ-Cer(d18:1/16:0) or its isomers	$C_{72}H_{130}N_2O_{32}$		
	26	1482.90535	-	1482.90537	-0.01	-	$[M-H]^-$	GalNAcβ1-4(NeuGcα2-3)Galβ1-4Glcβ-Cer(d18:1/24:0) or its isomers	$C_{73}H_{133}N_3O_{27}$		
	27	1574.88002	-	1574.87994	0.05	-	$[M-H]^-$	Fucα2-3GlcNAcβ1-6GalNAcβ1-3Galα1-	$C_{74}H_{133}N_3O_{32}$		

Classification	No.	Measured *m/z*		Calculated *m/z*	Error (ppm)		Assignment			Ref.	Structurally specific CID ions (*m/z*)[a)]
		MCAEF	Common spray		MCAEF	Common spray	Ion form	Compound	Molecular formula		
								4Galβ1-4Glcβ-Cer(d18:1/16:0) or its isomers			
	28	1590.87456	1590.87464	1590.87486	-0.19	-0.14	$[M-H]^-$	Galβ1-3(GlcNAcβ16)GalNAcβ1-3Galα1-4Galβ1-4Glcβ-Cer(d18:1/16:0) or its isomers	$C_{74}H_{133}N_3O_{33}$		
	29	1581.86649	-	1581.86646	0.19	-	$[M+Cl]^-$	Fucα1-2Galβ1-3(Fucα1-4)GlcNAcβ1-3Galβ1-4Glcβ-Cer(d18:1/18:0) or its isomers	$C_{74}H_{134}N_2O_{31}$		
	30	1577.87971	1577.87941	1577.87961	0.06	-0.13	$[M-H]^-$	GalNAcβ1-3Galα1-3Galα1-3Galα1-4Galβ1-4Glcβ-Cer(d18:1/18:0) or its isomers	$C_{74}H_{134}N_2O_{33}$		
	31	1512.91623	-	1512.91593	0.20	-	$[M-H]^-$	GalNAcα1-3GalNAcβ1-3Galα1-4Galβ1-4Glcβ-Cer(d18:1/22:0) or its isomers	$C_{74}H_{135}N_3O_{28}$		
	32	1483.92575	-	1483.92577	-0.01	-	$[M-H]^-$	Fucα1-2Galβ1-3GalNAcβ1-4Galβ1-4Glcβ-Cer(d18:1/24:0) or its isomers	$C_{74}H_{136}N_2O_{27}$		
	33	1470.93049	-	1470.93052	-0.02	-	$[M-H]^-$	Fucα1-2Galα1-3Galα1-4Galβ1-4Glcβ-Cer(d18:1/26:0) or its isomers	C74H137NO27		
	34	1486.92525	-	1486.92544	-0.13	-	$[M-H]^-$	Galα1-3Galα1-3Galα1-4Galβ1-4Glcβ-Cer(d18:1/26:0) or its isomers	$C_{74}H_{137}NO_{28}$		
	35	1540.94732	-	1540.94723	0.06	-	$[M-H]^-$	GalNAcα1-3GalNAcβ1-3Galα1-4Galβ1-4Glcβ-Cer(d18:1/24:0) or its isomers	$C_{76}H_{139}N_3O_{28}$		
	36	1527.95192	-	1527.95199	-0.05	-	$[M-H]^-$	Galβ1-3GalNAcβ1-3Galα1-4Galβ1-4Glcβ-Cer(d18:1/26:0) or its isomers	$C_{76}H_{140}N2O_{28}$		
	37	1633.94217	-	1633.94220	-0.02	-	$[M-H]^-$	GalNAcβ1-3Galα1-3Galα1-3Galα1-4Galβ1-4Glcβ-Cer(d18:1/22:0) or its isomers	$C_{78}H_{142}N_2O_{33}$		
	38	1805.91939	-	1805.91961	-0.12	-	$[M+K-2H]^-$	GalNAcβ1-3Galα1-3Galα1-3Galα1-3Galα1-4Galβ1-4Glcβ-Cer(d18:1/20:0) or its isomers	$C_{82}H_{148}N_2O_{38}$		
Acidic glycosphingolipids											
	1	778.51458	778.51499	778.51446	0.15	0.68	$[M-H]^-$	ST(d18:1/16:0)	$C_{40}H_{77}NO_{11}S$		
	2	806.54593	806.54616	806.54576	0.21	0.50	$[M-H]^-$	ST(d18:1/18:0)	$C_{42}H_{81}NO_{11}S$	[38, 39]	
	3	834.57754	834.57728	834.57706	0.58	0.26	$[M-H]^-$	ST(d18:1/20:0)	$C_{44}H_{85}NO_{11}S$	[39]	
	4	850.57232	850.57144	850.57197	0.41	-0.62	$[M-H]^-$	ST(d18:1/h20:0)	$C_{44}H_{85}NO_{12}S$	[21]	
	5	862.60869	862.60839	862.60836	0.38	0.03	$[M-H]^-$	ST(d18:1/22:0)	$C_{46}H_{89}NO_{11}S$	[21, 38,]	

Classification	No.	Measured *m/z* MCAEF	Measured *m/z* Common spray	Calculated *m/z*	Error (ppm) MCAEF	Error (ppm) Common spray	Assignment Ion form	Assignment Compound	Assignment Molecular formula	Ref.	Structurally specific CID ions (*m/z*)[a]
										39	
	6	878.60340	878.60305	878.60327	0.15	-0.25	$[M-H]^-$	ST(d18:1/h22:0)	$C_{46}H_{89}NO_{12}S$	21	
	7	888.62408	888.62383	888.62401	0.08	-0.20	$[M-H]^-$	ST(d18:1/24:1)	$C_{48}H_{91}NO_{11}S$	21, 38, 39, 42-44	241, 257, 259, 390, 774, 888
	8	904.61894	904.61895	904.61892	0.02	0.03	$[M-H]^-$	ST(d18:1/h24:1)	$C_{48}H_{91}NO_{12}S$	21, 38, 39, 45	241, 257, 259, 540, 568, 778, 904
	9	890.63970	890.63916	890.63966	0.04	-0.56	$[M-H]^-$	ST(d18:1/24:0)	$C_{48}H_{93}NO_{11}S$	21, 38	
	10	906.63461	906.63431	906.63457	0.04	-0.29	$[M-H]^-$	ST(d18:1/h24:0)	$C_{48}H_{93}NO_{12}S$	21, 26, 38	241, 522, 540, 568, 906
	11	1151.70585	1151.70546	1151.70588	-0.03	-0.36	$[M-H]^-$	NeuAcα2-3Galβ1-4Glcβ-Cer(d18:1/16:0) or its isomers	C57H104N2O21		
	12	1179.73728	1179.73678	1179.73718	0.08	-0.34	$[M-H]^-$	NeuAcα2-3Galβ1-4Glcβ-Cer(d18:1/18:0) or its isomers	$C_{59}H_{108}N2O_{21}$		
	13	1204.69761	-	1204.69782	-0.17	-	$[M+K-2H]^-$	KDNα2-3Galβ1-4Glcβ-Cer(d18:1/20:0) or its isomers	$C_{59}H_{109}NO_{21}$		
	14	1235.79970	-	1235.79978	-0.06	-	$[M-H]^-$	NeuAcα2-3Galβ1-4Glcβ-Cer(d18:1/22:0) or its isomers	$C_{63}H_{116}N_2O_{21}$		
	15	1354.78521	-	1354.78525	-0.03	-	$[M-H]^-$	NeuAcα2-6GalNAcβ1-4Galβ1-4Glcβ-Cer(d18:1/16:0) or its isomers	$C_{65}H_{117}N_3O_{26}$		
	16	1261.81550	1261.81565	1261.81543	0.06	0.17	$[M-H]^-$	NeuAcα2-3Galβ1-4Glcβ-Cer(d18:1/24:1) or its isomers	C65H118N2O21		
	17	1377.76688	-	1377.76668	0.15	-	$[M+Cl]^-$	Galβ1-4(NeuAcα2-3)Galβ1-4Glcβ-Cer(d18:1/18:0) or its isomers	$C_{65}H_{118}N_2O_{26}$		
	18	1263.83114	-	1263.83108	0.05	-	$[M-H]^-$	NeuAcα2-3Galβ1-4Glcβ-Cer(d18:1/24:0) or its isomers	$C_{65}H_{120}N_2O_{21}$		
	19	1382.81619	-	1382.81656	-0.27	-	$[M-H]^-$	GalNAcβ1-4(NeuAcα2-3)Galβ1-4Glcβ-Cer(d18:1/18:0) or its isomers	$C_{67}H_{121}N_3O_{26}$		
	20	1552.81489	-	1552.81476	0.08	-	$[M+Cl]^-$	NeuAcα2-3GalNAcβ1-3Galα1-4Galβ1-4Glcβ-Cer(d18:1/16:0) or its isomers	$C_{71}H_{127}N_3O_{31}$		
	21	1568.80974	-	1568.80967	0.04	-	$[M+Cl]^-$	NeuGcα2-3Galβ1-3GalNAcβ1-4Galβ1-4Glcβ-Cer(d18:1/16:0) or its isomers	$C_{71}H_{127}N_3O_{32}$		
	22	1544.87051<br>1580.84612	1544.86993<br>-	1544.86938<br>1580.84606	0.73<br>0.04	0.36<br>-	$[M-H]^-$<br>$[M+Cl]^-$	NeuAcα2-3GalNAcβ1-3Galα1-4Galβ1-4Glcβ-Cer(d18:1/18:0) or its isomers	$C_{73}H_{131}N_3O_{31}$		

Classification	No.	Measured m/z		Calculated m/z	Error (ppm)		Assignment			Ref.	Structurally specific CID ions (m/z)[a)]
		MCAEF	Common spray		MCAEF	Common spray	Ion form	Compound	Molecular formula		
	23	1492.92625	-	1492.92611	0.09	-	[M-H]⁻	NeuAcα2-6GalNAcβ1-4Galβ1-4Glcβ-Cer(d18:1/26:1) or its isomers	$C_{75}H_{135}N_3O_{26}$		
	24	1572.90095	1572.90067	1572.90068	0.17	-0.01	[M-H]⁻	NeuAcα2-3GalNAcβ1-3Galα1-4Galβ1-4Glcβ-Cer(d18:1/20:0) or its isomers	$C_{75}H_{135}N_3O_{31}$		
	25	1588.89546	-	1588.89559	-0.08	-	[M-H]⁻	NeuGcα2-3Galβ1-3GalNAcβ1-4Galβ1-4Glcβ-Cer(d18:1/20:0) or its isomers	$C_{75}H_{135}N_3O_{32}$		
	26	1494.94152	-	1494.94176	-0.16	-	[M-H]⁻	NeuAcα2-6GalNAcβ1-4Galβ1-4Glcβ-Cer(d18:1/26:0) or its isomers	$C_{75}H_{137}N_3O_{26}$		
	27	1510.93672	-	1510.93667	0.03	-	[M-H]⁻	GalNAcβ1-4(NeuGcα2-3)Galβ1-4Glcβ-Cer(d18:1/26:0) or its isomers	$C_{75}H_{137}N_3O_{27}$		
	28	1554.92665	-	1554.92650	0.10	-	[M-H]⁻	NeuAcα2-8NeuAcα2-3Galβ1-4Glcβ-Cer(d18:1/24:0) or its isomers	$C_{76}H_{137}N_3O_{29}$		
	29	1635.93417	-	1635.93432	-0.09	-	[M+Na-2H]⁻	KDNα2-3Galβ1-3GalNAcβ1-4Galβ1-4Glcβ-Cer(d18:1/26:1) or its isomers	$C_{79}H_{142}N_2O_{31}$		
	30	1789.95910	-	1789.95931	-0.12	-	[M-H]⁻	NeuAcα2-8NeuAcα2-8NeuAcα2-3Galβ1-4Glcβ-Cer(d18:1/20:0) or its isomers	$C_{83}H_{146}N_4O_{37}$		
	31	1873.92059	-	1873.92068	-0.05s	-	[M+K-2H]⁻	Galβ1-3GalNAcβ1-4(NeuAcα2-8NeuAcα2-3) Galβ1-4Glcβ-Cer(d18:1/18:0) or its isomers	$C_{84}H_{148}N_4O_{39}$		
	32	1847.93015	-	1847.93018	-0.02	-	[M+K-2H]⁻	KDNα2-3Galβ1-3GalNAcβ1-4(KDNα2-3)Galβ1-4Glcβ-Cer(d18:1/22:0) or its isomers	$C_{84}H_{150}N_2O_{39}$		
	33	1857.96214	-	1857.96214	0.00	-	[M+K-2H]⁻	GalNAcβ1-4(NeuGcα2-3)Galβ1-3GalNAcβ1-4Galβ1-4Glcβ-Cer(d18:1/22:0) or its isomers	$C_{85}H_{152}N_4O_{37}$		
Other glycosphingolipids (Gangliosides)											
	1	1207.76873	1207.76858	1207.76848	0.21	0.08	[M-H]⁻	Ganglioside GM3 (d38:1)	$C_{61}H_{112}N_2O_{21}$	46	
	2	1209.78461	-	1209.78413	0.40	-	[M-H]⁻	Ganglioside GM3 (d38:0)	$C_{61}H_{114}N_2O_{21}$		
	3	1251.75846	-	1251.75831	0.12	-	[M-H]⁻	Ganglioside GA1 (d36:2)	$C_{62}H_{112}N_2O_{23}$		
	4	1233.78435	1233.78413	1233.78413	0.18	0.00	[M-H]⁻	Ganglioside GM3 (d40:2)	$C_{63}H_{114}N_2O_{21}$		
	5	1235.79959	-	1235.79978	-0.15	-	[M-H]⁻	Ganglioside GM3 (d40:1)	$C_{63}H_{116}N_3O_{21}$	46	
	6	1265.84657	-	1265.84673	-0.13	-	[M-H]⁻	Ganglioside GM3 (d42:0)	$C_{65}H_{122}N_2O_{21}$		
	7	1414.77016	-	1414.77000	0.11	-	[M-H]⁻	Ganglioside GD3 (d32:1)	$C_{66}H_{117}N_3O_{29}$		

Classification	No.	Measured *m/z*		Calculated *m/z*	Error (ppm)		Assignment			Ref.	Structurally specific CID ions (*m/z*)[a)]
		MCAEF	Common spray		MCAEF	Common spray	Ion form	Compound	Molecular formula		
	8	1480.79347	-	1480.79363	-0.11	-	$[M+Cl]^-$	Ganglioside GD3 (d34:0)	$C_{68}H_{123}N_3O_{29}$		
	9	1542.85387	1542.85352	1542.85373	0.09	-0.14	$[M-H]^-$	Ganglioside GM1 (36:2)	$C_{73}H_{129}N_3O_{31}$	39	
	10	1529.92327	-	1529.92318	0.06	-	$[M+Cl]^-$	Ganglioside GM2 (d44:1)	$C_{76}H_{138}N_2O_{26}$		
	11	1902.97241	-	1902.97238	0.02	-	$[M+K-2H]^-$	Ganglioside GD1b (d38:0)	$C_{87}H_{155}N_3O_{39}$		
**Neutral Lipids**											
**Glycerolipids**											
Monoacylglycerols (MAGs)											
	1	357.30124	-	357.30103	0.59	-	$[M-H]^-$	MG(18:0)	$C_{21}H_{42}O_4$		
	2	405.30110	-	405.30103	0.17	-	$[M-H]^-$	MG(22:4)	$C_{25}H_{42}O_4$		
Diacylglycerols (DAGs)											
	1	559.43680	-	559.43680	0.00	-	$[M-H]^-$	DG(32:4)	$C_{35}H_{60}O_5$		
	2	661.48384	-	661.48375	0.14	-	$[M-H]^-$	DG(40:9)	$C_{43}H_{66}O_5$		
	3	663.49948	-	663.49940	0.12	-	$[M-H]^-$	DG(40:8)	$C_{43}H_{68}O_5$		
	4	685.48376	-	685.48375	0.01	-	$[M-H]^-$	DG(42:11)	$C_{45}H_{66}O_5$		
	5	687.49979	687.49956	687.49940	0.57	0.23	$[M-H]^-$	DG(42:10)	$C_{45}H_{68}O_5$		
	6	713.51541	713.51505	713.51505	0.50	0.00	$[M-H]^-$	DG(44:11)	$C_{47}H_{70}O_5$		
Triacylglycerols (TAGs)											
	1	715.58838	715.58886	715.58821	0.24	0.91	$[M-H]^-$	TG(42:3)	$C_{45}H_{80}O_6$		
	2	797.66658	-	797.66646	0.15	-	$[M-H]^-$	TG(48:4)	$C_{51}H_{90}O_6$		
	3	799.68206	-	799.68211	-0.06	-	$[M-H]^-$	TG(48:3)	$C_{51}H_{92}O_6$		
	4	893.66448	-	893.66406	0.47	-	$[M+Na-2H]^-$	TG(54:9)	$C_{57}H_{92}O_6$		
Glycosyldiradylglycerols											
	1	793.51402	793.51380	793.51412	-0.12	-0.40	$[M-H]^-$	SQDG(32:0)	$C_{41}H_{78}O_{12}S$		
	2	759.46847	-	759.46889	-0.55	-	$[M-H]^-$	MGDG-O(16:3)	$C_{43}H_{68}O_{11}$		

Classification	No.	Measured *m/z*		Calculated *m/z*	Error (ppm)		Assignment			Ref.	Structurally specific CID ions (*m/z*)[a)]
		MCAEF	Common spray		MCAEF	Common spray	Ion form	Compound	Molecular formula		
**Sterol Lipids**											
	1	411.18463	411.18473	411.18468	-0.12	0.12	$[M-H]^-$	17-Hydroxypregnenolone sulfate or its isomers	$C_{21}H_{32}O_6S$		
	2	397.20212	-	397.20205	0.18	-	$[M-H]^-$	3,7,12-Trioxochola-1,4-dien-24-oic Acidor its isomers	$C_{24}H_{30}O_5$		
	3	445.18682	-	445.18679	0.07	-	$[M-H]^-$	Estrone 3-glucuronide or its isomers	$C_{24}H_{30}O_8$		
	4	403.24913	403.24921	403.24900	0.32	0.52	$[M-H]^-$	3α-Hydroxy-7,12-dioxo-5β-cholan-24-oic Acid or its isomers	$C_{24}H_{36}O_5$		
	5	375.29056	-	375.29047	0.24	-	$[M-H]^-$	Lithocholic acid or its isomers	$C_{24}H_{40}O_3$		
	6	473.25766	-	473.25785	-0.40	-	$[M-H]^-$	3α,7α,12α-trihydroxy-5α-cholan-24-ylsulfate or its isomers	$C_{24}H_{42}O_7S$		
	7	498.28965	498.28951	498.28948	0.34	0.06	$[M-H]^-$	Taurochenodeoxycholic acid or its isomers	$C_{26}H_{45}NO_6S$		
	8	1051.54578	1051.54530	1051.54591	-0.12	-0.58	$[M+Na-2H]^-$	Parrisaponin or its isomers	$C_{52}H_{86}O_{20}$		
	9	1209.59089	-	1209.59097	-0.07	-	$[M-H]^-$	3-O-(Rhaa1-3Glcb1-2(Xylb1-3)Glcb1-4Galb)-(25R)-12-oxo-5α-spirostan-3β-ol or its isomers	$C_{57}H_{94}O_{27}$		
**Fatty acyls (FAs)**											
Fatty acids											
	1	255.23294	255.23285	255.23295	-0.04	-0.39	$[M-H]^-$	FA(16:0)	$C_{16}H_{32}O_2$		
	2	279.23289	279.23299	279.23295	-0.21	0.14	$[M-H]^-$	FA(18:2)	$C_{18}H_{32}O_2$		
	3	281.24863	281.24853	281.24860	0.11	-0.25	$[M-H]^-$	FA(18:1)	$C_{18}H_{34}O_2$		
	4	283.26417	283.26409	283.26425	-0.28	-0.56	$[M-H]^-$	FA(18:0)	$C_{18}H_{36}O_2$		
	5	303.23275	303.23310	303.23295	-0.66	0.49	$[M-H]^-$	FA(20:4)	$C_{20}H_{32}O_2$		
	6	311.29556	-	311.29555	0.03	-	$[M-H]^-$	FA(20:0)	$C_{20}H_{40}O_2$		
	7	327.23286	327.23292	327.23295	-0.28	-0.09	$[M-H]^-$	FA(22:6)	$C_{22}H_{32}O_2$		
Other fatty acyls											
	1	179.14427	-	179.14414	0.73	-	$[M-H]^-$	Fatty alcohols or its isomers	$C_{12}H_{20}O$		

Classification	No.	Measured *m/z*		Calculated *m/z*	Error (ppm)		Assignment			Ref.	Structurally specific CID ions (*m/z*)[a)]
		MCAEF	Common spray		MCAEF	Common spray	Ion form	Compound	Molecular formula		
**Others lipids**											
	1	182.05876	-	182.05877	-0.05	-	$[M-H]^-$	Phosphorylcholine	$C_5H_{14}NO_4P$		
**Number of Lipids**							**MCAEF: 421 *vs.* common spray: 180**				
**Other compounds**											
	1	346.05553	-	346.05581	-0.81	-	$[M-H]^-$	Adenosine monophosphate (AMP)	$C_{10}H_{14}N_5O_7P$		
	2	912.17863	-	912.17869	-0.07	-	$[M+Na-2H]^-$	Octenoyl CoA	$C_{29}H_{48}N_7O_{17}P_3S$		
	3	1114.44726	-	1114.44715	0.10	-	$[M-H]^-$	CoA(24:1)	$C_{45}H_{80}N_7O_{17}P_3S$		
	4	1130.44239	-	1130.44206	0.29	-	$[M-H]^-$	CoA(24:0)	$C_{45}H_{80}N_7O_{18}P_3S$		
	5	243.02749	243.02733	243.02753	-0.16	-0.82	$[M-H]^-$	β-L-Fucose 1-phosphate	$C_6H_{13}O_8P$		
	6	245.04317	245.04326	245.04318	-0.04	0.33	$[M-H]^-$	Phosphatidyl glycerol	$C_6H_{15}O_8P$		
	7	399.04689	399.04632	399.04629	0.10	0.08	$[M-H]^-$	Bis(glycerophospho)-glycerol	$C_9H_{22}O_{13}P_2$		
**Number of other compounds**							**MCAEF: 7 *vs.* common spray: 3**				

a), Structurally specific CID ions of extracted lipids were detected by LC-MS/MS using CID. Red fragment ions were detected in the positive ion mode, and blue fragment ions were detected in the negative ion mode.

Supplementary Information Table S4. Putative protein detection on rat brain tissue sections by MALDI-TOF/TOF MS in the positive-ion mode with and without MCAEF, using sinapinic acid as the matrix.

Protein ion signals (m/z)	MCAEF	Common spray method
3538	✓	✗
3574	✓	✗
3675	✓	✗
3722	✓	✗
3738	✓	✗
3751	✓	✗
3793	✓	✗
3856	✓	✗
3891	✓	✗
4380	✓	✗
4437	✓	✓
4565	✓	✗
4615	✓	✗
4742	✓	✓
4820	✓	✗
4850	✓	✗
4866	✓	✗
4958	✓	✗
4977	✓	✓
4999	✓	✓
5013	✓	✗
5036	✓	✗
5130	✓	✗
5290	✓	✓
5300	✓	✗
5340	✓	✗
5400	✓	✗
5461	✓	✗
5481	✓	✓
5520	✓	✗
5545	✓	✓
5562	✓	✓
5601	✓	✓
5618	✓	✗
5631	✓	✗

Protein ion signals (m/z)	MCAEF	Common spray method
5900	✓	✓
5924	✓	✗
5979	✓	✗
6061	✓	✗
6075	✓	✗
6128	✓	✗
6271	✓	✓
6334	✓	✓
6418	✓	✗
6540	✓	✗
6575	✓	✓
6588	✓	✗
6644	✓	✗
6715	✓	✗
6786	✓	✗
6908	✓	✓
6979	✓	✓
6986	✓	✗
6997	✓	✓
7018	✓	✗
7034	✓	✓
7050	✓	✗
7057	✓	✓
7075	✓	✓
7083	✓	✗
7097	✓	✗
7104	✓	✗
7136	✓	✗
7147	✓	✗
7282	✓	✗
7378	✓	✗
7531	✓	✗
7541	✓	✓
7558	✓	✓
7573	✓	✓
7595	✓	✓
7700	✓	✗
7707	✓	✗
7720	✓	✓
7736	✓	✗

Protein ion signals (m/z)	MCAEF	Common spray method
7759	✓	✗
7803	✓	✓
7840	✓	✓
7856	✓	✓
7927	✓	✓
7978	✓	✓
8016	✓	✓
8034	✓	✗
8073	✓	✓
8096	✓	✗
8120	✓	✓
8259	✓	✗
8339	✓	✗
8417	✓	✗
8450	✓	✓
8492	✓	✗
8562	✓	✓
8597	✓	✓
8664	✓	✓
8685	✓	✓
8713	✓	✗
8779	✓	✗
8810	✓	✗
8910	✓	✓
8924	✓	✓
8956	✓	✓
8967	✓	✓
9119	✓	✗
9132	✓	✓
9147	✓	✗
9176	✓	✓
9197	✓	✓
9203	✓	✓
9212	✓	✗
9243	✓	✓
9300	✓	✓
9503	✓	✓
9559	✓	✓
9663	✓	✓
9935	✓	✓

Protein ion signals (m/z)	MCAEF	Common spray method
9976	✓	✓
10013	✓	✗
10198	✓	✗
10253	✓	✓
10370	✓	✗
10590	✓	✗
10607	✓	✓
10652	✓	✓
11078	✓	✗
11537	✓	✓
11963	✓	✓
12062	✓	✗
12130	✓	✓
12146	✓	✓
12163	✓	✗
12260	✓	✓
12291	✓	✗
12308	✓	✓
12327	✓	✓
12351	✓	✗
12367	✓	✗
12410	✓	✓
12434	✓	✗
13421	✓	✗
13466	✓	✗
13575	✓	✓
13789	✓	✗
13810	✓	✓
13820	✓	✓
13965	✓	✓
14003	✓	✓
14045	✓	✗
14121	✓	✓
14200	✓	✓
14235	✓	✗
14281	✓	✓
14328	✓	✗
14344	✓	✓
14393	✓	✗
14405	✓	✗

Protein ion signals (m/z)	MCAEF	Common spray method
14973	✓	✗
15110	✓	✓
15152	✓	✓
15176	✓	✓
15195	✓	✓
15234	✓	✓
15268	✓	✗
15357	✓	✗
15399	✓	✓
15404	✓	✗
15418	✓	✗
15432	✓	✗
15820	✓	✓
15824	✓	✓
15852	✓	✓
15856	✓	✓
15875	✓	✗
15896	✓	✗
15900	✓	✗
15954	✓	✓
15967	✓	✓
16050	✓	✗
16107	✓	✗
16152	✓	✓
16190	✓	✗
16234	✓	✓
16253	✓	✓
16263	✓	✗
17089	✓	✓
17115	✓	✗
17144	✓	✓
17164	✓	✗
17207	✓	✗
17222	✓	✗
17259	✓	✗
17274	✓	✓
17334	✓	✓
17351	✓	✓
17371	✓	✓
17390	✓	✓

Protein ion signals (m/z)	MCAEF	Common spray method
17412	✓	✓
17424	✓	✓
17452	✓	✓
18061	✓	✓
18083	✓	✓
18164	✓	✓
18185	✓	✗
18207	✓	✓
18237	✓	✓
18261	✓	✓
18319	✓	✓
18342	✓	✓
18400	✓	✓
18477	✓	✓
18489	✓	✓
18521	✓	✓
18604	✓	✓
19825	✓	✗
21415	✓	✓
21491	✓	✓
21641	✓	✓
21802	✓	✗
21891	✓	✓
23365	✓	✓
24607	✓	✗
24755	✓	✓
25520	✓	✗
26154	✓	✗
28246	✓	✓
28408	✓	✓
28735	✓	✗
29216	✓	✗
30355	✓	✗
31243	✓	✗
32493	✓	✓
35507	✓	✗
36731	✓	✗
**Total number of proteins/peptides**	**232**	**119**

"✓" means the protein/peptide molecule could be detected; "✗" means the protein/peptide molecule could not be detected.

References

1. R. Casadonte and R. M. Caprioli, *Nat. Protoc.*, 2011, 6, 1695-1709.
2. X. Wang, J. Han, A. Chou, J. Yang, J. Pan and C. H. Borchers, *Anal. Chem.*, 2013, 85, 7566-7573.
3. X. Wang, J. Han, J. Pan and C. H. Borchers, *Anal. Chem.*, 2014, 86, 638-646.
4. J. L. Norris, D. S. Cornett, J. A. Mobley, M. Andersson, E. H. Seeley, P. Chaurand and R. M. Caprioli, *Int. J. Mass Spectrom.*, 2007, 260, 212-221.
5. J. Han, R. M. Danell, J. R. Patel, D. R. Gumerov, C. O. Scarlett, J. P. Speir, C. E. Parker, I. Rusyn, S. Zeisel and C. H. Borchers, *Metabolomics*, 2008, 4, 128-140.
6. R. Tautenhahn, K. Cho, W. Uritboonthai, Z. Zhu, G. J. Patti and G. Siuzdak, *Nature Biotech.*, 2012, 30, 826-828.
7. S. Subramaniam and G. Hsiao, *Nature Immunol.*, 2012, 13, 199-203.
8. N. J. Spann, L. X. Garmire, J. G. McDonald, D. S. Myers, S. B. Milne, N. Shibata, D. Reichart, J. N. Fox, I. Shaked, D. Heudobler, C. R. Raetz, E. W. Wang, S. L. Kelly, M. C. Sullards, R. C. Murphy, A. H. Merrill, Jr., H. A. Brown, E. A. Dennis, A. C. Li, K. Ley, S. Tsimikas, E. Fahy, S. Subramaniam, O. Quehenberger, D. W. Russell and C. K. Glass, *Cell*, 2012, 151, 138-152.
9. M. Malavolta, F. Bocci, E. Boselli and N. G. Frega, *J. Chromatog. B. Analyt. Technol.Biomed. Life Sci.*, 2004, 810, 173-186.
10. T. Houjou, K. Yamatani, M. Imagawa, T. Shimizu and R. Taguchi, *Rapid Comm. Mass Spectrom.*, 2005, 19, 654-666.
11. R. Taguchi, M. Nishijima and T. Shimizu, *Meth. Enzymol.*, 2007, 432, 185-211.

12. C. A. Smith, G. O'Maille, E. J. Want, C. Qin, S. A. Trauger, T. R. Brandon, D. E. Custodio, R. Abagyan and G. Siuzdak, *Ther. Drug Monitor.*, 2005, 27, 747-751.

13. D. S. Wishart, C. Knox, A. C. Guo, R. Eisner, N. Young, B. Gautam, D. D. Hau, N. Psychogios, E. Dong, S. Bouatra, R. Mandal, I. Sinelnikov, J. Xia, L. Jia, J. A. Cruz, E. Lim, C. A. Sobsey, S. Shrivastava, P. Huang, P. Liu, L. Fang, J. Peng, R. Fradette, D. Cheng, D. Tzur, M. Clements, A. Lewis, A. De Souza, A. Zuniga, M. Dawe, Y. Xiong, D. Clive, R. Greiner, A. Nazyrova, R. Shaykhutdinov, L. Li, H. J. Vogel and I. Forsythe, *Nucl. Acids Res.*, 2009, 37, D603-610.

14. D. S. Wishart, T. Jewison, A. C. Guo, M. Wilson, C. Knox, Y. Liu, Y. Djoumbou, R. Mandal, F. Aziat, E. Dong, S. Bouatra, I. Sinelnikov, D. Arndt, J. Xia, P. Liu, F. Yallou, T. Bjorndahl, R. Perez-Pineiro, R. Eisner, F. Allen, V. Neveu, R. Greiner and A. Scalbert, *Nucl. Acids Res.*, 2013, 41, D801-807.

15. E. Fahy, M. Sud, D. Cotter and S. Subramaniam, *Nucl. Acids Res.*, 2007, 35, W606-612.

16. M. Sud, E. Fahy, D. Cotter, A. Brown, E. A. Dennis, C. K. Glass, A. H. Merrill, Jr., R. C. Murphy, C. R. Raetz, D. W. Russell and S. Subramaniam, *Nucl. Acids Res.*, 2007, 35, D527-532.

17. C. H. Le, J. Han and C. H. Borchers, *Anal. Chem.*, 2012, 84, 8391-8398.

18. E. Astigarraga, G. Barreda-Gomez, L. Lombardero, O. Fresnedo, F. Castano, M. T. Giralt, B. Ochoa, R. Rodriguez-Puertas and J. A. Fernandez, *Anal. Chem.*, 2008, 80, 9105-9114.

19. K. A. Berry, J. A. Hankin, R. M. Barkley, J. M. Spraggins, R. M. Caprioli and R. C. Murphy, *Chem. Rev.*, 2011, 111, 6491-6512.

20. S. N. Jackson, H. Y. Wang and A. S. Woods, *J. Am. Soc. Mass Spectrom.*, 2005, 16, 2052-2056.

21. C. D. Cerruti, F. Benabdellah, O. Laprevote, D. Touboul and A. Brunelle, *J. Am. Soc. Mass Spectrom.*, 2012, 84, 2164-2171.

22. S. Cha and E. S. Yeung, *Anal. Chem.*, 2007, 79, 2373-2385.

23. A. M. Delvolve, B. Colsch and A. S. Woods, *Anal. Methods*, 2011, 3, 1729-1736.

24. T. Hayasaka, N. Goto-Inoue, Y. Sugiura, N. Zaima, H. Nakanishi, K. Ohishi, S. Nakanishi, T. Naito, R. Taguchi and M. Setou, *Rapid Comm. Mass Spectrom.*, 2008, 22, 3415-3426.

25. R. R. Landgraf, M. C. Prieto Conaway, T. J. Garrett, P. W. Stacpoole and R. A. Yost, *Anal. Chem.*, 2009, 81, 8488-8495.

26. S. R. Shanta, L. H. Zhou, Y. S. Park, Y. H. Kim, Y. Kim and K. P. Kim, *Anal. Chem.*, 2011, 83, 1252-1259.

27. Y. Sugiura and M. Setou, *Rapid Comm. Mass Spectrom.*, 2009, 23, 3269-3278.

28. H. Enomoto, Y. Sugiura, M. Setou and N. Zaima, *Anal. Bioanal. Chem.*, 2011, 400, 1913-1921.

29. T. Hayasaka, N. Goto-Inoue, N. Zaima, Y. Kimura and M. Setou, *Lipids*, 2009, 44, 837-848.

30. T. J. Garrett and R. A. Yost, *Anal. Chem.*, 2006, 78, 2465-2469.

31. J. A. Hankin, R. M. Barkley and R. C. Murphy, *J. Am. Soc. Mass Spectrom.*, 2007, 18, 1646-1652.

32. J. Matsumoto, Y. Sugiura, D. Yuki, T. Hayasaka, N. Goto-Inoue, N. Zaima, Y. Kunii, A. Wada, Q. Yang, K. Nishiura, H. Akatsu, A. Hori, Y. Hashizume, T. Yamamoto, K. Ikemoto, M. Setou and S. Niwa, *Anal. Bioanal. Chem.*, 2011, 400, 1933-1943.

33. R. J. Goodwin, A. R. Pitt, D. Harrison, S. K. Weidt, P. R. Langridge-Smith, M. P. Barrett and C. Logan Mackay, *Rapid Comm. Mass Spectrom.*, 2011, 25, 969-972.

34. J. E. Vance and D. E. Vance, *Biochem. Cell Biol.*, 2004, 82, 113-128.

35. N. Zaima, T. Sasaki, H. Tanaka, X. W. Cheng, K. Onoue, T. Hayasaka, N. Goto-Inoue, H. Enomoto, N. Unno, M. Kuzuya and M. Setou, *Atherosclerosis*, 2011, 217, 427-432.

36. Y. Kobayashi, T. Hayasaka, M. Setou, H. Itoh and N. Kanayama, *Placenta*, 2010, 31, 245-248.

37. S. N. Jackson, H. Y. Wang, A. S. Woods, M. Ugarov, T. Egan and J. A. Schultz, *J. Am. Soc. Mass Spectrom.*, 2005, 16, 133-138.

38. S. N. Jackson, H. Y. Wang and A. S. Woods, *J. Am. Soc. Mass Spectrom.*, 2007, 18, 17-26.

39. L. J. Sparvero, A. A. Amoscato, C. E. Dixon, J. B. Long, P. M. Kochanek, B. R. Pitt, H. Bayir and V. E. Kagan, *Chem. Phys. Lipids*, 2012, 165, 545-562.

40. H. Bayir, V. A. Tyurin, Y. Y. Tyurina, R. Viner, V. Ritov, A. A. Amoscato, Q. Zhao, X. J. Zhang, K. L. Janesko-Feldman, H. Alexander, L. V. Basova, R. S. Clark, P. M. Kochanek and V. E. Kagan, *Ann. Neurol.*, 2007, 62, 154-169.

41. V. A. Tyurin, Y. Y. Tyurina, W. Feng, A. Mnuskin, J. Jiang, M. Tang, X. Zhang, Q. Zhao, P. M. Kochanek, R. S. Clark, H. Bayir and V. E. Kagan, *J. Neurochem.*, 2008, 107, 1614-1633.

42. T. J. Garrett, M. C. Prieto-Conaway, V. Kovtoun, H. Bui, N. Izgarian, G. Stafford and R. A. Yost, *Int. J. Mass Spectrom.*, 2007, 260, 166-176.

43. R. C. Murphy, J. A. Hankin and R. M. Barkley, *J. Lipid Res.*, 2009, 50 Suppl, S317-322.

44. Y. Chen, J. Allegood, Y. Liu, E. Wang, B. Cachon-Gonzalez, T. M. Cox, A. H. Merrill, Jr. and M. C. Sullards, *Anal. Chem.*, 2008, 80, 2780-2788.

45. S. Shimma, Y. Sugiura, T. Hayasaka, N. Zaima, M. Matsumoto and M. Setou, *Anal. Chem.*, 2008, 80, 878-885.

46. K. Ikeda, T. Shimizu and R. Taguchi, *J. Lipid Res.*, 2008, 49, 2678-2689.
